# Supplementary material for: A Neuronal Relay Mediates a Nutrient Responsive Gut/Fat Body Axis Regulating Energy Homeostasis in Adult Drosophila
Source: Cell Metab. 2019 Feb 5;29(2):269–284.e10. doi: 10.1016/j.cmet.2018.09.021 (PMC6370946; doi:10.1016/j.cmet.2018.09.021)
Supplement: Document S1. Figures S1–S6 and Table S1 [file mmc1.pdf]

Cell Metabolism, Volume *29*

Supplemental Information

A Neuronal Relay Mediates a Nutrient Responsive Gut/Fat Body Axis Regulating Energy Homeostasis in Adult *Drosophila*

Alessandro Scopelliti, Christin Bauer, Yachuan Yu, Tong Zhang, Björn Kruspig, Daniel J. Murphy, Marcos Vidal, Oliver D.K. Maddocks, and Julia B. Cordero

Supplementary Figure Legends

Figure S1. Related to Figure 1: Bursα is not detectable in adult CNS. (A) Bursα immunofluorescence (green/grey) in the CNS of dark pupae and 10-14d old adult fed and 24h starved wild type flies. Nc82 staining labels the neuropil (red). Scalebars = 200 µm. (B) Relative *bursα* mRNA levels in midguts only and gut-less wild type flies (10-14d old) fully fed or following 24h starvation. (C) Relative *Glut1* mRNA levels in midguts of flies following 14d of transgene activation. Data represents the average of at least 3 biological replicates. Statistical analysis was done by unpaired t-test. Bars represent mean ± SEM. (D) Immunostaining of adult posterior midguts from fed and starved flies bearing ee-specific overexpression of *bursα* for 10-14d. Bursα (red/white). Unless otherwise noted DAPI (blue) was used to stain all cell nuclei. (E) Relative TAG levels in 14d old animals of indicated genotypes. 5 females were collected for each biological replicate. Data for each genotype represents the mean of 3 biological replicates ± SEM. Statistical analysis was done by one-way ANOVA followed by Turkey’s multiple comparisons test.

Figure S2. Related to Figure 2: Disruption of Bursα/DLgr2 signalling does not affect food intake, absorption and excretion or locomotor activity. (A, B) Food intake assay between Zeitgeber (ZT) 22-24 in 3d old adults (A) and adults following 14d transgene activation (B) by measuring Allura Red absorbance in whole fly extracts. 25 animals were analysed per genotype per biological replicate. Data represents the average of 3 biological replicates ± SEM. Statistical analysis was done through unpaired t-test. (C, D) Fluorescent intensity of 2-NBDG accumulation in whole fly extracts from animals of ages and genotypes as in A, B. 5 animals were analysed

per genotype per biological replicate. Data represents the average of a minimum of 3 biological replicates ± SEM. Statistical analysis was done through unpaired t-test. (E-G) Glucose (E), TAG (F) and FFA levels (G) levels in excrements of adult animals following 14d of transgene activation. 5 animals were analysed per genotype per biological replicate. Data represents the average of at least 3 biological replicates ± SEM. Statistical analysis was done through unpaired t-test. (H) Relative circulating glucose levels in 3d old animals. Significances are shown compared to their own control. Data for each genotype represents the mean of 3 biological replicates ± SEM. Statistical analysis was done through unpaired t-test. (I, J) Relative circulating TAG (I) and FFA (J) in flies following 14d of transgene activation. 5 females were collected for each biological replicate. Data for each genotype represents the mean of 3 biological replicates ± SEM. Statistical analysis was done by unpaired t-test. (K, L) Results from video-tracking analysis to assess locomotor activity of animals following 14d transgene activation (K) and 3d old whole mutant animals (L). We assessed the locomotion of a 4 or 5 individual flies per genotype. Each experimental genotype was compared to its own control. Data for each genotype represents mean values ± SEM. Statistical analysis was done through unpaired t-test (K) and one-way ANOVA followed by Turkey's multiple comparisons test.

Figure S3. Related to Figure 3: Bursα signalling via VM, fat body or tracheal DLgr2 is dispensable for metabolic regulation. (A) Immunofluorescent staining of adult posterior midguts (R4-R5 transition) expressing *CD8-GFP* (green) under the control of a *dlgr2*-Gal4 driver. (B, I, K, M) Starvation survival analysis in animals started 10d after transgene activation. Total number of animals assessed (n) is indicated within the graphs. Log-rank (Mantel-Cox) test was used to analyse statistical

significance. (C) Relative circulating glucose levels in animals following 14d of transgene activation. Data for each genotype represents the mean of 3 biological replicates ± SEM. Statistical analysis was done through unpaired t-test. (D, E, H, L, N, O) Relative TAG levels from adult animals following 14d of transgene activation (D, H, L, N) or from 7d old whole mutant animals with or without concomitant expression of the indicated transgenes (E, O). 5 females were collected for each biological replicate. Data for each genotype represents the mean of 3 biological replicates ± SEM. Statistical analysis was done by unpaired t-test (D, H, L, N) and one-way ANOVA followed by Turkey's multiple comparisons test (E, O). (F, G) Quantifications from Western Blots of haemolymph Bursα. Data represents the mean from 3 independent experiments of the type shown in Fig. 3A, B ± SEM. Statistical analysis was done through unpaired t-test. (J) Immunofluorescent staining in fat bodies from adults expressing nuclear *Red stinger* (red) under the control of a *dlgr2*-Gal4 driver.

Figure S4. Related to Figures 3 and 4: Loss of lipid storage upon neuronal knockdown of *dlgr2* is independent from food intake, absorption and excretion. (A) Food intake assessment within ZT 22-24 in flies of the indicated genotypes analysed by measuring Allura Red absorbance in whole fly extracts. 25 animals were analysed per genotype per biological replicate. Data represents the average of 3 biological replicates ± SEM. Statistical analysis was done through unpaired t-test. (B, C) Relative glucose (B) and FFA levels (C) in the excrement in flies of indicated genotypes following 14d of transgene activation. 5 animals were analysed per genotype per biological replicate. Data represents the average of 3 biological replicates ± SEM. Statistical analysis was done through unpaired t-test. (D) Intestinal glucose absorption in flies of genotypes as in (A-C) measured by analysing 2-NBDG

fluorescent intensity in whole fly extracts. 5 animals were analysed per genotype per biological replicate. Data represents the average of at least 3 biological replicates ± SEM. Statistical analysis was done through unpaired t-test. (E) Chill Coma Recovery Time was recorded in animals following 3d adult specific transgene activation. Total number of animals assessed (n) is indicated within the graphs. Log-rank (Mantel-Cox) test was used to analyse statistical significance. (F) Relative oxygen consumption of whole fly mitochondrial extracts from animals following 3d adult specific transgene activation. 4 whole animals where used per biological replicate. Data for each genotype represents the mean of 4 biological replicates ± SEM. Statistical analysis was done through paired t-test.

Figure S5. Related to Figures 3 and 4: Insulin signalling is not affected by Bursα/ neuronal DLgr2. (A, B) qRT-PCRs for *inr* and *thor* relative to *sdha* in cuticles (A) and for *dilp2*, *3* and *5,* relative to *actin5c* in heads (B). Significances are shown relative to their individual control. Data represents the average of at least 3 biological replicates. Statistical analysis was done by unpaired t-test. Bars represent mean ± SEM. (C) Immunofluorescence staining in fat bodies of adults expressing the Insulin sensor tGPH (green). All experiments were carried out 14d following activation of the indicated transgenes.

Figure S6. Related to Figure 5: Indirect regulation of AKH/AKHR signalling by DLgr2. (A) Immunofluorescence staining of adult Corpora Cardiaca neurons expressing *Myr-GFP* under the control of a *dlgr2*-Gal4 driver (green) and co-stained with anti-AKH antibody (red). DAPI (blue). Scalebars= 100 μm (left) and 20 μm (right). (B) Starvation survival test in animals of indicated genotypes following transgene

activation for 10d. Total number of animals assessed (n) is indicated within the graphs. Log-rank (Mantel-Cox) test was used to analyse statistical significance. (C) Relative TAG levels in whole flies of the indicated genotypes following 14d transgene activation (C). 5 females were collected for each biological replicate. Data for each genotype represents the mean of 3 biological replicates ± SEM. Statistical analysis was done by unpaired t-test. (D) Immunofluorescent staining in Corpora Cardiaca neurons expressing *CD8-RFP* under the *nSyb*-Gal4 driver (red) and co-stained with anti-AKH antibody (green). DAPI (blue). (E) *akh* transcript levels relative to *rpl32* in whole animals following 14d transgene activation. Data represents the average of 3 biological replicates. Statistical analysis was done by unpaired t-test. Bars represent mean ± SEM. (F-I) Relative TAG levels in 14d old animals of the indicated genotypes (UAS-RNAi-only controls for Fig. 5F) (F) or in 7d old animals of indicated genotypes (UAS-RNAi-only controls for Fig. 5M) (G-I). 5 females were collected for each biological replicate. Data for each genotype represents the mean of 3 biological replicates ± SEM. Statistical analysis was done by one-way ANOVA followed by Turkey's multiple comparisons test. (J) pH3 staining to visualize ISC proliferation in posterior midguts of animals of indicated genotypes following 14d of transgene activation. Animals were either fully fed (black) or fed followed by 24h starvation prior to pH3 assessment (red). A minimum of 9 midguts were analysed per genotype per condition. Each dot in the graph (n) represents an individual posterior midgut. n values from left to right = 16 (+, Fed and Starved); 13 (*Glut1 IR*, Fed); 14 (*Glut1 IR*, Starved); 9 (*bursα IR*, Fed); 21 (*bursα IR*, Starved); 10 (+, Fed); 20 (+, Starved); 10 (*osbp*, Fed); 11 (*osbp*, Starved). Statistical analysis was done by unpaired t-test. Bars represent mean ± SEM.

Table S1: Primer sequences used in this study. Related to STAR Methods.

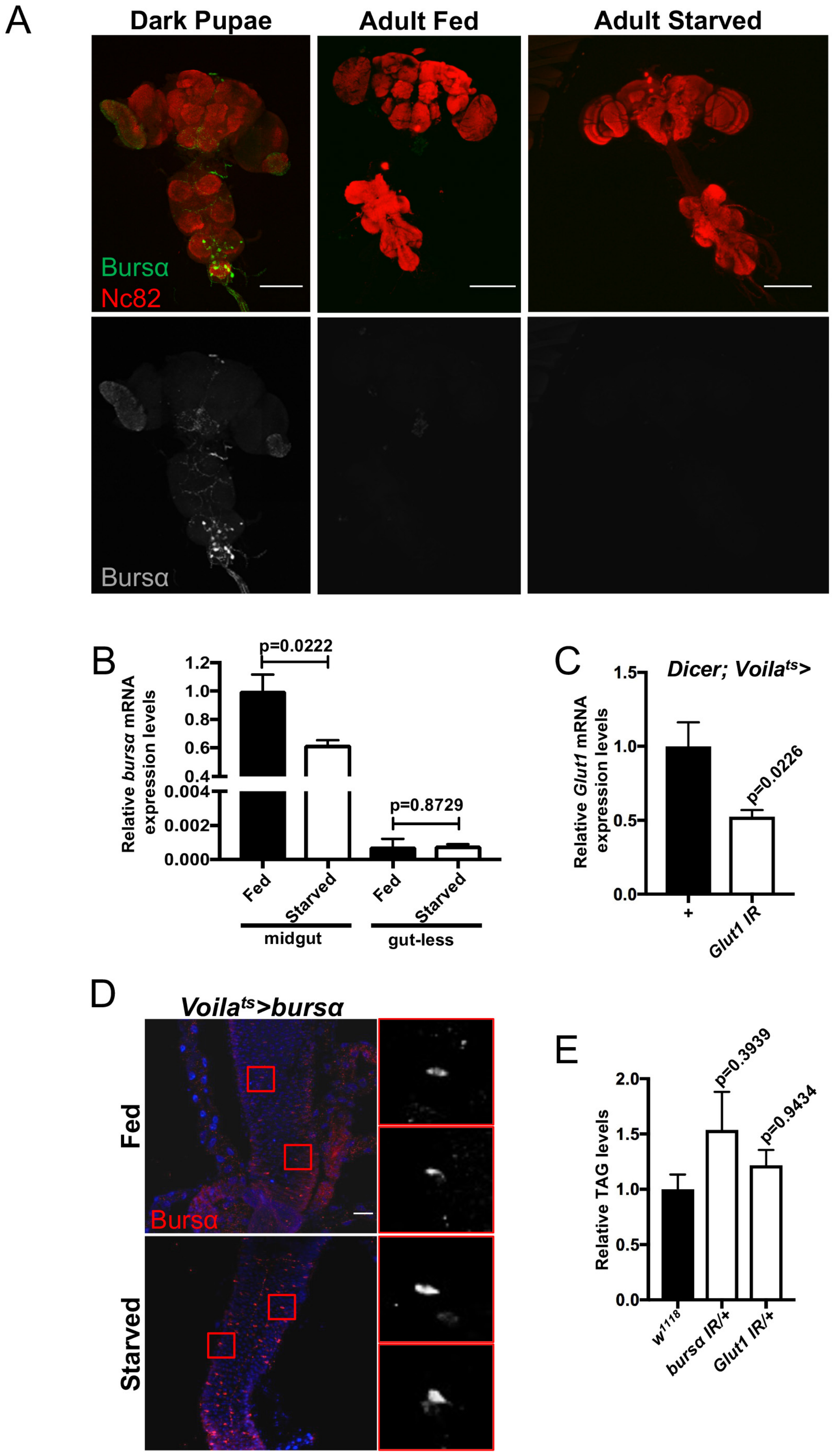
A
Dark Pupae
Adult Fed
Adult Starved
Bursα
Nc82
Bursα
B
p=0.0222
p=0.8729
Relative bursα mRNA expression levels
1.2
1.0
0.8
0.6
0.4
0.004
0.002
0.000
Fed
Starved
Fed
Starved
midgut
gut-less
C
Dicer; Voila^ts>
Relative Glut1 mRNA expression levels
1.5
1.0
0.5
0.0
p=0.0226
+
Glut1 IR
D
Voila^ts>bursα
Fed
Starved
Bursα
E
Relative TAG levels
2.0
1.5
1.0
0.5
0.0
p=0.3939
p=0.9434
w1118
bursα IR/+
Glut1 IR/+

Figure S1

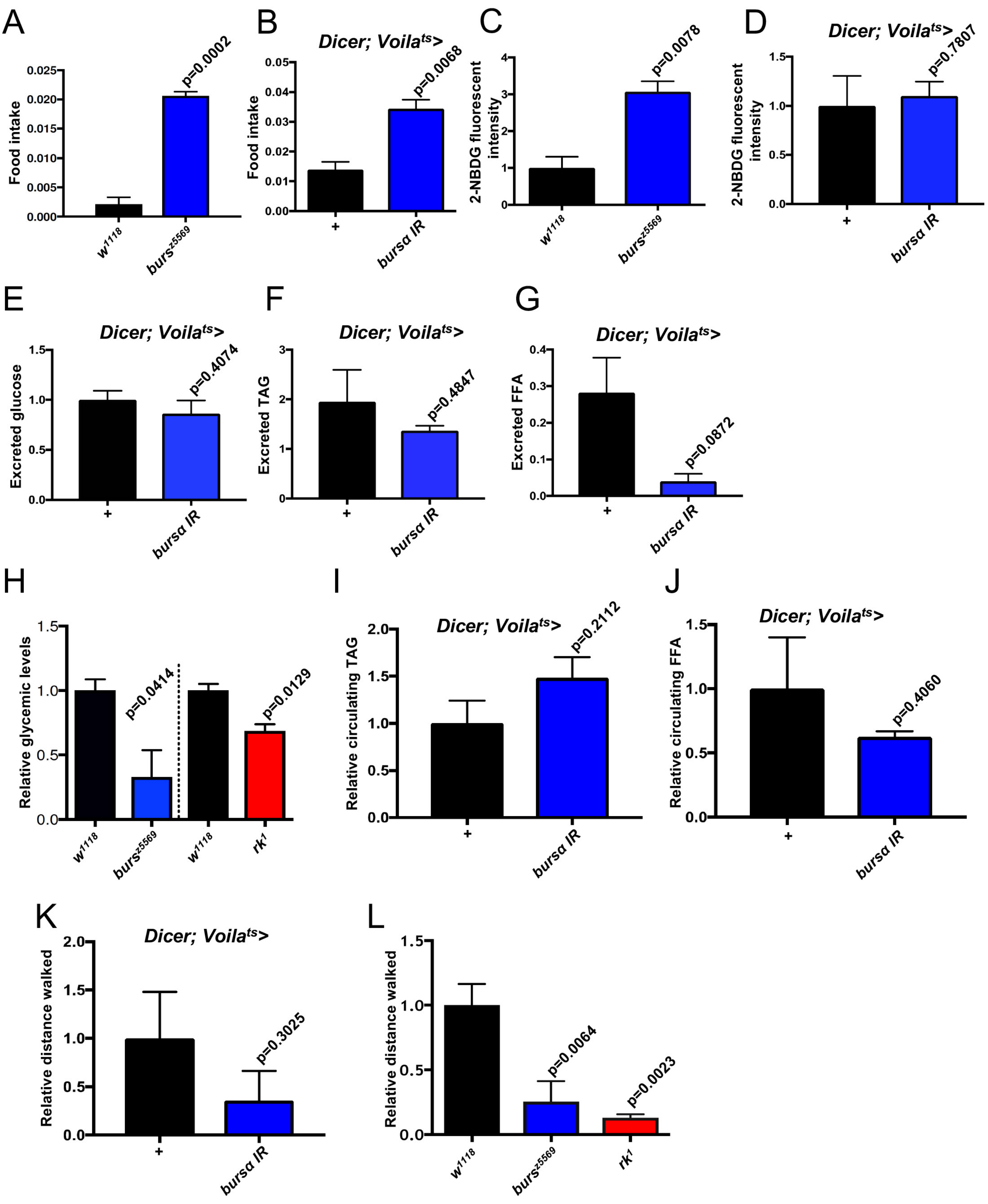
A
Food intake
0.025
0.020
0.015
0.010
0.005
0.000
p=0.0002
w1118
bursz5569
B
Dicer; Voilats>
Food intake
0.05
0.04
0.03
0.02
0.01
0.00
p=0.0068
+
bursα IR
C
2-NBDG fluorescent intensity
4
3
2
1
0
p=0.0078
w1118
bursz5569
D
Dicer; Voilats>
2-NBDG fluorescent intensity
1.5
1.0
0.5
0.0
p=0.7807
+
bursα IR
E
Dicer; Voilats>
Excreted glucose
1.5
1.0
0.5
0.0
p=0.4074
+
bursα IR
F
Dicer; Voilats>
Excreted TAG
3
2
1
0
p=0.4847
+
bursα IR
G
Dicer; Voilats>
Excreted FFA
0.4
0.3
0.2
0.1
0.0
p=0.0872
+
bursα IR
H
Relative glycemic levels
1.5
1.0
0.5
0.0
p=0.0414
p=0.0129
w1118
bursz5569
w1118
rk1
I
Dicer; Voilats>
Relative circulating TAG
2.0
1.5
1.0
0.5
0.0
p=0.2112
+
bursα IR
J
Dicer; Voilats>
Relative circulating FFA
1.5
1.0
0.5
0.0
p=0.4060
+
bursα IR
K
Dicer; Voilats>
Relative distance walked
2.0
1.5
1.0
0.5
0.0
p=0.3025
+
bursα IR
L
Relative distance walked
1.5
1.0
0.5
0.0
p=0.0064
p=0.0023
w1118
bursz5569
rk1

Figure S2

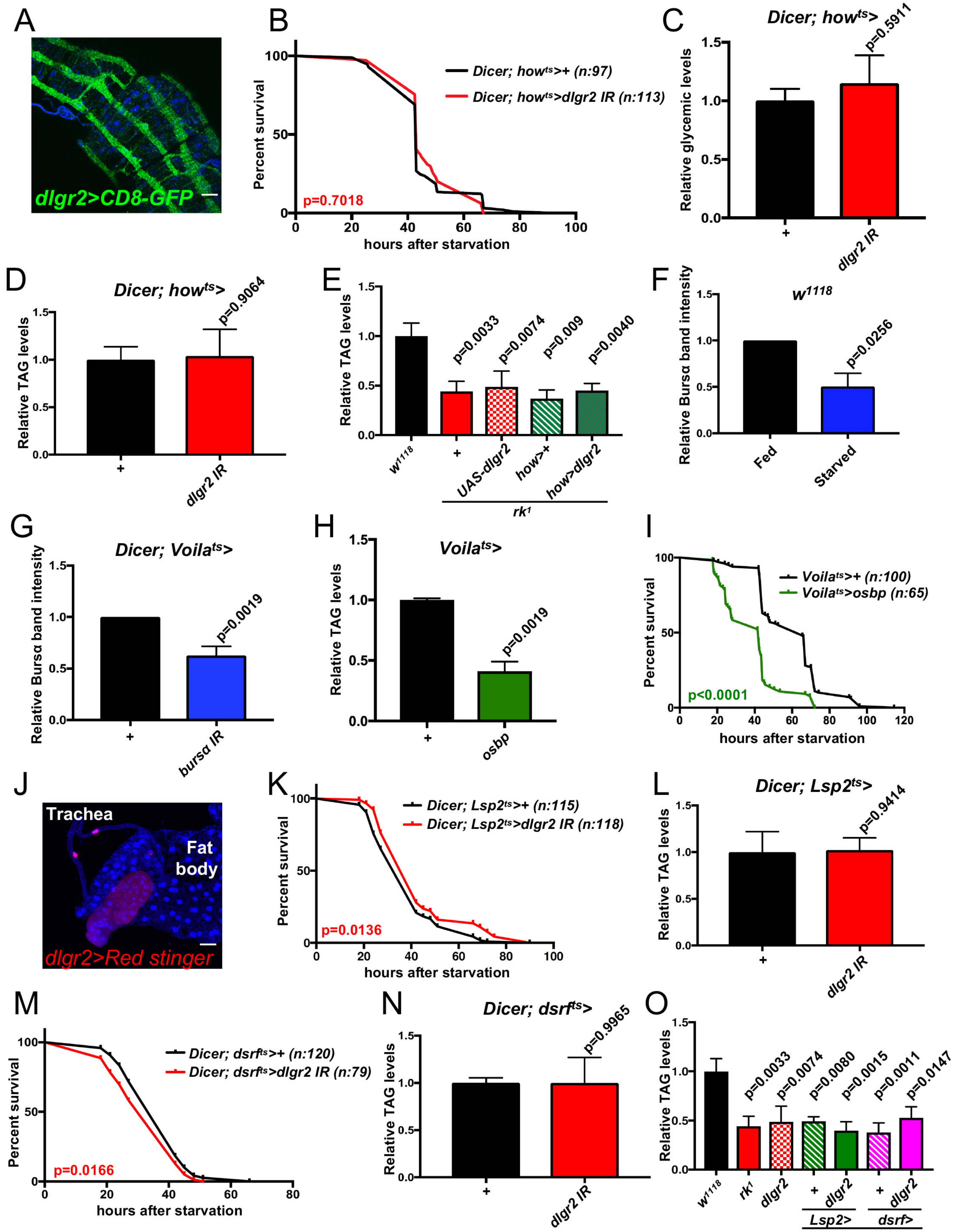
A
dlgr2>CD8-GFP
B
Dicer; howts>+ (n:97)
Dicer; howts>dlgr2 IR (n:113)
Percent survival
hours after starvation
p=0.7018
C
Dicer; howts>
p=0.5911
Relative glycemic levels
+
dlgr2 IR
D
Dicer; howts>
p=0.9064
Relative TAG levels
E
p=0.0033
p=0.0074
p=0.009
p=0.0040
w1118
UAS-dlgr2
how>+
how>dlgr2
rk1
F
w1118
p=0.0256
Relative Bursα band intensity
Fed
Starved
G
Dicer; Voilats>
p=0.0019
bursα IR
H
Voilats>
osbp
I
Voilats>+ (n:100)
Voilats>osbp (n:65)
p<0.0001
J
Trachea
Fat body
dlgr2>Red stinger
K
Dicer; Lsp2ts>+ (n:115)
Dicer; Lsp2ts>dlgr2 IR (n:118)
p=0.0136
L
Dicer; Lsp2ts>
p=0.9414
M
Dicer; dsrfts>+ (n:120)
Dicer; dsrfts>dlgr2 IR (n:79)
p=0.0166
N
Dicer; dsrfts>
p=0.9965
O
p=0.0033
p=0.0074
p=0.0080
p=0.0015
p=0.0011
p=0.0147
rk1
dlgr2
Lsp2>
dsrf>

Figure S3

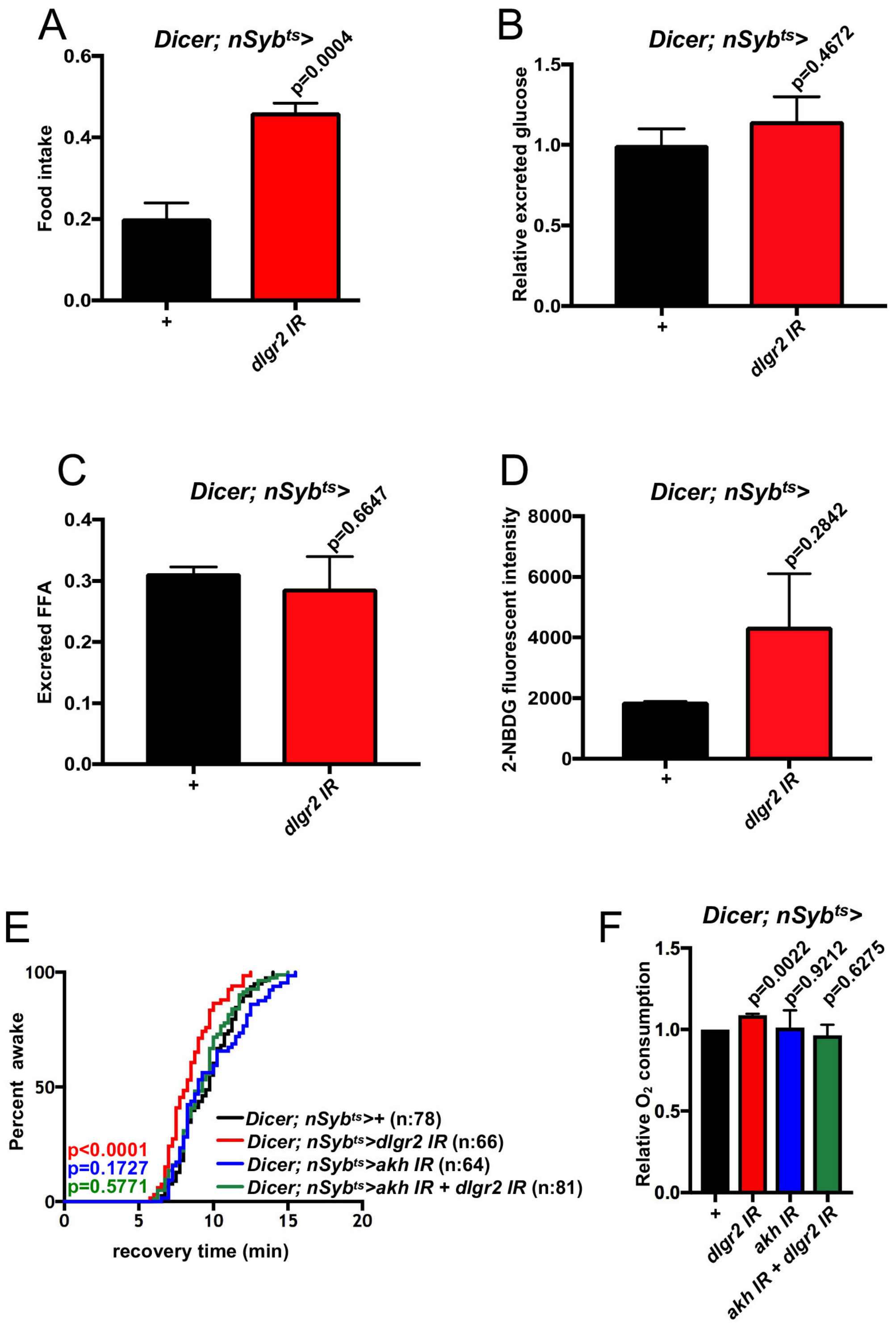
A
Dicer; nSybts>
p=0.0004
Food intake
0.6
0.4
0.2
0.0
+
dlgr2 IR
B
Dicer; nSybts>
p=0.4672
Relative excreted glucose
1.5
1.0
0.5
0.0
+
dlgr2 IR
C
Dicer; nSybts>
p=0.6647
Excreted FFA
0.4
0.3
0.2
0.1
0.0
+
dlgr2 IR
D
Dicer; nSybts>
p=0.2842
2-NBDG fluorescent intensity
8000
6000
4000
2000
0
+
dlgr2 IR
E
Percent awake
100
50
0
p<0.0001
p=0.1727
p=0.5771
Dicer; nSybts>+ (n:78)
Dicer; nSybts>dlgr2 IR (n:66)
Dicer; nSybts>akh IR (n:64)
Dicer; nSybts>akh IR + dlgr2 IR (n:81)
0
5
10
15
20
recovery time (min)
F
Dicer; nSybts>
p=0.0022
p=0.9212
p=0.6275
Relative O2 consumption
1.5
1.0
0.5
0.0
+
dlgr2 IR
akh IR
akh IR + dlgr2 IR

Figure S4

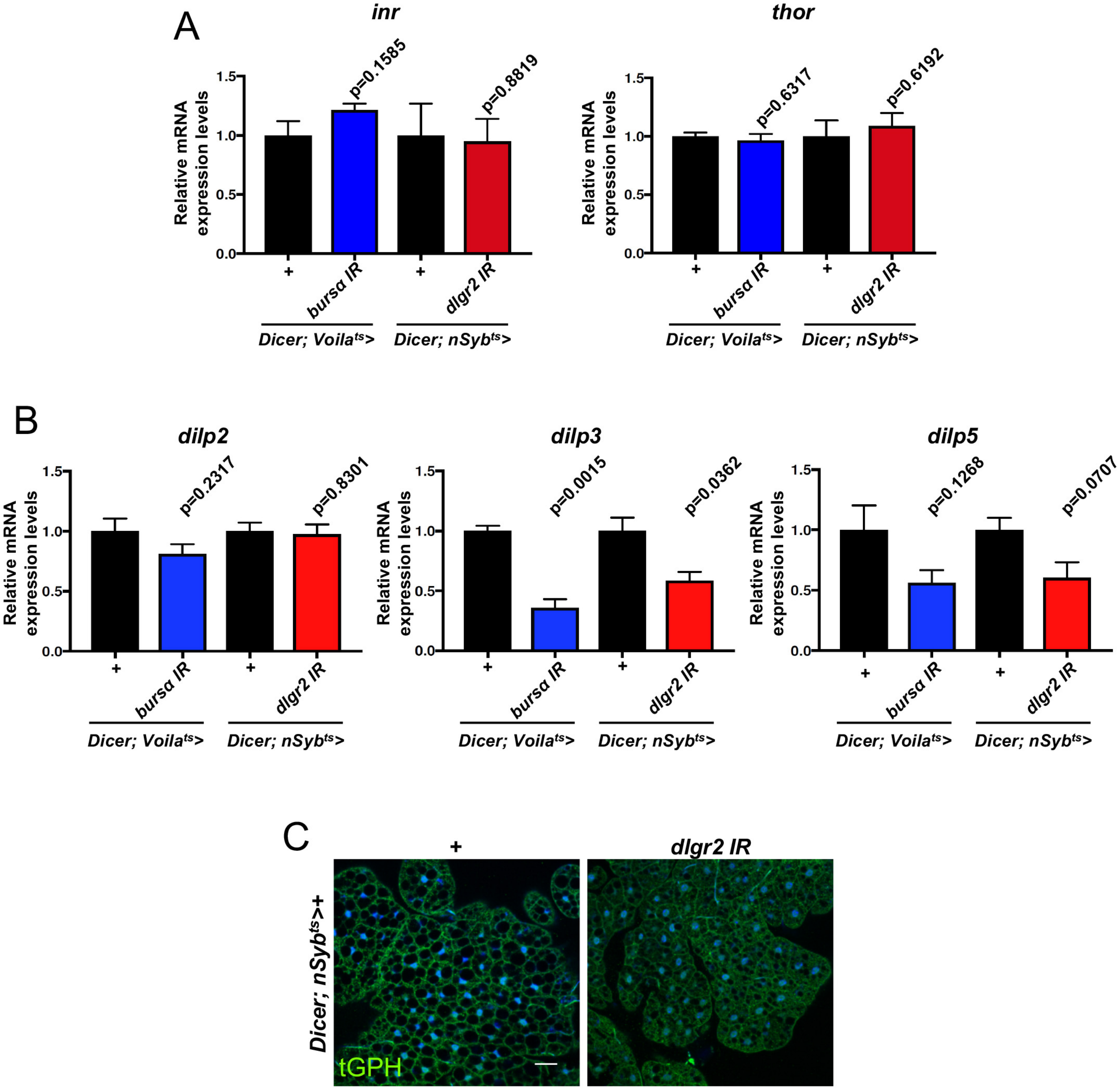
A
inr
thor
Relative mRNA expression levels
p=0.1585
p=0.8819
p=0.6317
p=0.6192
+
bursα IR
dlgr2 IR
Dicer; Voila^ts>
Dicer; nSyb^ts>
B
dilp2
dilp3
dilp5
p=0.2317
p=0.8301
p=0.0015
p=0.0362
p=0.1268
p=0.0707
C
dlgr2 IR
Dicer; nSyb^ts>+
tGPH

Figure S5

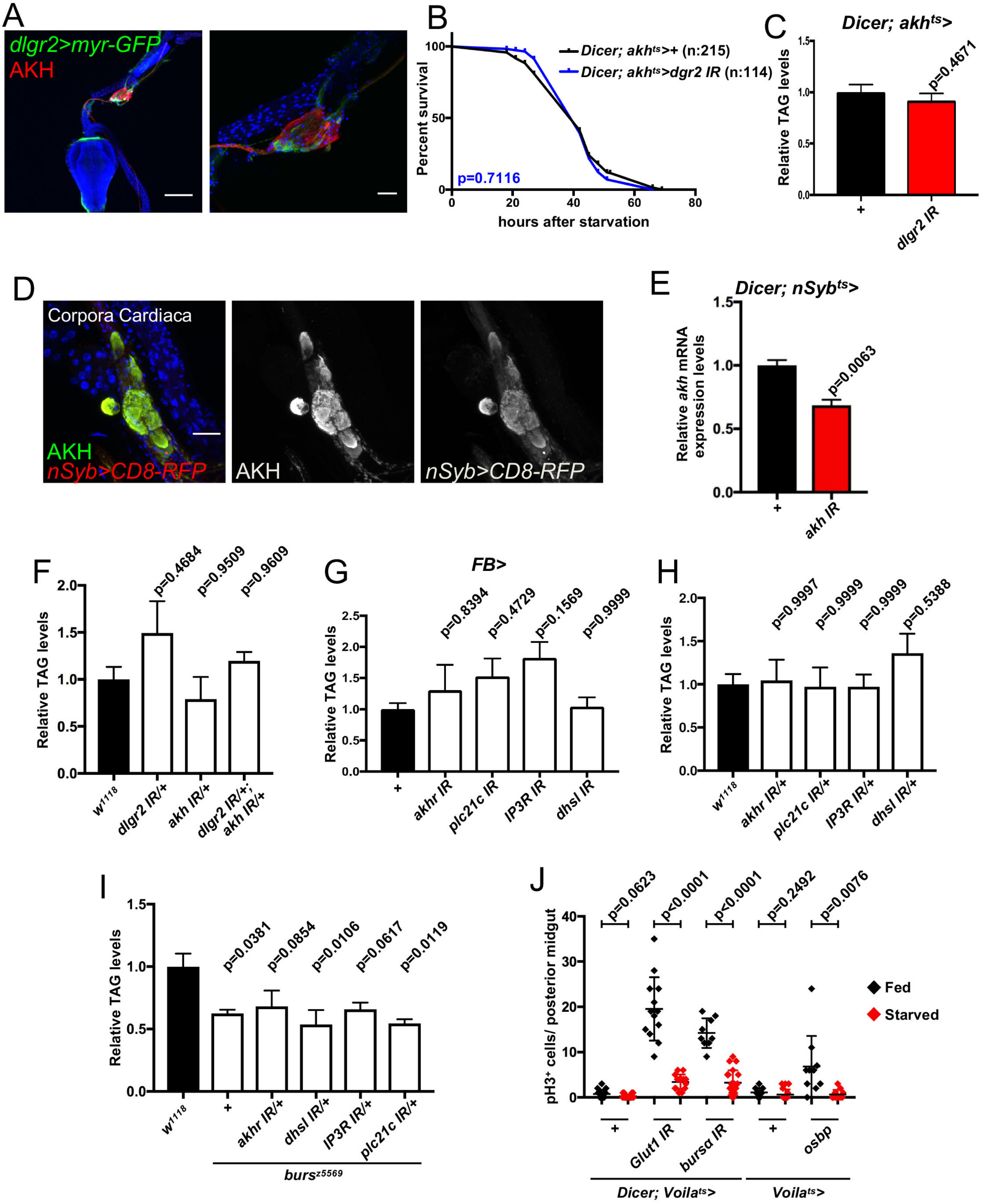
A
dlgr2>myr-GFP
AKH
B
Percent survival
hours after starvation
Dicer; akhts>+ (n:215)
Dicer; akhts>dgr2 IR (n:114)
p=0.7116
C
Dicer; akhts>
Relative TAG levels
p=0.4671
+
dlgr2 IR
D
Corpora Cardiaca
AKH
nSyb>CD8-RFP
E
Dicer; nSybts>
Relative akh mRNA expression levels
p=0.0063
akh IR
F
Relative TAG levels
p=0.4684
p=0.9509
p=0.9609
w1118
dlgr2 IR/+
akh IR/+
dlgr2 IR/+; akh IR/+
G
FB>
p=0.8394
p=0.4729
p=0.1569
p=0.9999
akhr IR
plc21c IR
IP3R IR
dhsl IR
H
p=0.9997
p=0.9999
p=0.9999
p=0.5388
akhr IR/+
plc21c IR/+
IP3R IR/+
dhsl IR/+
I
p=0.0381
p=0.0854
p=0.0106
p=0.0617
p=0.0119
dhsl IR/+
IP3R IR/+
plc21c IR/+
bursz5569
J
pH3+ cells/ posterior midgut
p=0.0623
p<0.0001
p<0.0001
p=0.2492
p=0.0076
Fed
Starved
Glut1 IR
bursα IR
osbp
Dicer; Voilats>
Voilats>

Figure S6

Target	Primer name	Sequence 5' → 3'
rpl32	rpl32 F1	AGGCCCAAGATCGTGAAGAA
	rpl32 R1	TGTTGCACCAGGAACTTCTTGAA
actin5c	actin5c F1	GAGCGCGGTTACTCTTTCAC
	actin5c R1	CCATCTCCTGCTCAAAGTCG
sdha	sdha F1	AATGCCCAGATGACTATTGTGAG
	sdha R1	GCTTGCTGAAATCGTATTCATCC
bursα	burs F1	CATCCATGTGCTCCAGTATCC
	burs R1	GGCTTCACTTTGGGACAGAA
dilp2	dilp2 F1	CCTGCAGTTTGTCCAGGAGT
	dilp2 R1	AGCCAGGGAATTGAGTACACC
dilp3	dilp3 F1	GTATGGCTTCAACGCAATGAC
	dilp3 R1	GAGCATCTGAACCCAACTATCAC
dilp5	dilp5 F1	CGTGATCCCAGTTCTCCTGT
	dilp5 R1	ACCCTCAGCATGTCCATCAA
thor	thor F1	CCAGGAAGGTTGTCATCTCG
	thor R1	TGAAAGCCCGCTCGTAGATA
inr	inr F1	GGTGCTGGCATCATAGGTCT
	inr R1	CCTGCCTCTGAGTGATAGAAGG
akh	akh F1	GAAGTGGACCAGCATAGAACTC
	akh R1	GCGAGAAGGTCAATTGACAC

Table S1: Primer sequences used in this study. Related to STAR Methods.
