# Supplementary material for: A Neuronal Relay Mediates a Nutrient Responsive Gut/Fat Body Axis Regulating Energy Homeostasis in Adult Drosophila
Source: Cell Metab. 2019 Feb 5;29(2):269–284.e10. doi: 10.1016/j.cmet.2018.09.021 (PMC6370946; doi:10.1016/j.cmet.2018.09.021)
Supplement: Document S2. Article plus Supplemental Information [file mmc2.pdf]

Cell Metabolism

Article

A Neuronal Relay Mediates a Nutrient Responsive Gut/Fat Body Axis Regulating Energy Homeostasis in Adult *Drosophila*

Graphical Abstract

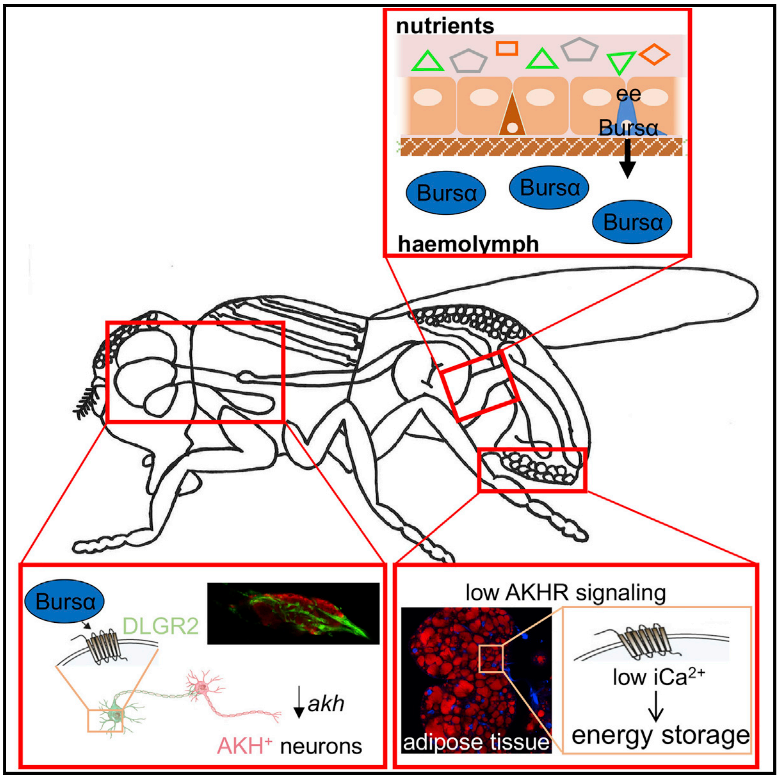

Authors

Alessandro Scopelliti, Christin Bauer, Yachuan Yu, ..., Marcos Vidal, Oliver D.K. Maddocks, Julia B. Cordero

Correspondence

a.scopelliti@beatson.gla.ac.uk (A.S.), julia.cordero@glasgow.ac.uk (J.B.C.)

In Brief

Scopelliti et al. identified a novel intestinal/neuronal/adipose tissue communication network in *Drosophila* with implications for endocrine-regulated metabolic homeostasis. In response to nutrients, enteroendocrine cells (EE) secrete the neuroendocrine peptide Bursicon α, which binds to its neuronal receptor DLgr2 to regulate organismal resistance to metabolic challenges through adipose glucagon-like AKHR signaling.

Highlights

- Enteroendocrine cells sense dietary sugars
- Enteroendocrine Bursicon α is secreted systemically in response to nutrients
- Bursα signals to its neuronal receptor DLgr2, triggering a relay with AKH neurons
- Bursα/DLgr2 preserve energetic homeostasis through fat body AKHR signaling

Scopelliti et al., 2019, Cell Metabolism *29*, 269–284
February 5, 2019 © 2018 The Author(s). Published by Elsevier Inc.
https://doi.org/10.1016/j.cmet.2018.09.021

A Neuronal Relay Mediates a Nutrient Responsive Gut/Fat Body Axis Regulating Energy Homeostasis in Adult *Drosophila*

Alessandro Scopelliti,[1,3,*] Christin Bauer,[1,3] Yachuan Yu,[1] Tong Zhang,[2] Björn Kruspig,[2] Daniel J. Murphy,[1,2] Marcos Vidal,[1] Oliver D.K. Maddocks,[2] and Julia B. Cordero[1,2,4,*]
[1]CRUK Beatson Institute, Garscube Estate, Switchback Road, Glasgow G61 1BD, UK
[2]Institute of Cancer Sciences, University of Glasgow, Garscube Estate, Switchback Road, Glasgow G61 1QH, UK
[3]These authors contributed equally
[4]Lead Contact
*Correspondence: a.scopelliti@beatson.gla.ac.uk (A.S.), julia.cordero@glasgow.ac.uk (J.B.C.)
https://doi.org/10.1016/j.cmet.2018.09.021

SUMMARY

The control of systemic metabolic homeostasis involves complex inter-tissue programs that coordinate energy production, storage, and consumption, to maintain organismal fitness upon environmental challenges. The mechanisms driving such programs are largely unknown. Here, we show that enteroendocrine cells in the adult *Drosophila* intestine respond to nutrients by secreting the hormone Bursicon α, which signals via its neuronal receptor DLgr2. Bursicon α/DLgr2 regulate energy metabolism through a neuronal relay leading to the restriction of glucagon-like, adipokinetic hormone (AKH) production by the corpora cardiaca and subsequent modulation of AKH receptor signaling within the adipose tissue. Impaired Bursicon α/DLgr2 signaling leads to exacerbated glucose oxidation and depletion of energy stores with consequent reduced organismal resistance to nutrient restrictive conditions. Altogether, our work reveals an intestinal/neuronal/adipose tissue inter-organ communication network that is essential to restrict the use of energy and that may provide insights into the physiopathology of endocrine-regulated metabolic homeostasis.

INTRODUCTION

Maintaining systemic energy homeostasis is crucial for the physiology of all living organisms. A balanced equilibrium between anabolism and catabolism involves tightly coordinated signaling networks and the communication between multiple organs (Gautron et al., 2015; Wang et al., 2014). Excess nutrients are stored in the liver and adipose tissue as glycogen and lipids, respectively. In times of high energy demand or low nutrient availability, nutrients are mobilized from storage tissues (Mattila and Hietakangas, 2017). Understanding how organs communicate to maintain systemic energy homeostasis is of critical importance, as its failure can result in severe metabolic disorders with life-threatening consequences.

The intestine is a key endocrine tissue and central regulator of systemic energy homeostasis. Enteroendocrine (ee) cells secrete multiple hormones in response to the nutritional status of the organism and orchestrate systemic metabolic adaptation across tissues. Recent work reveals greater than expected diversity (Haber et al., 2017), plasticity (Yan et al., 2017), and sensing functions of ee cells (Lebrun et al., 2017). Nevertheless, how ee cells respond to different environmental challenges and how they coordinate systemic responses is unclear. A better understanding of ee cell biology will directly impact our understanding of intestinal physiopathology, the regulation of systemic metabolism, and metabolic disorders.

Functional studies on inter-organ communication are often challenging in mammalian systems, due to their complex genetics and physiology. The adult *Drosophila* midgut has emerged as an invaluable model system to address key aspects of systemic physiology, host-pathogen interactions, stem cell biology and metabolism, among other things (Lemaitre and Miguel-Aliaga, 2013). As in its mammalian counterpart, the *Drosophila* adult intestinal epithelium displays multiple subtypes of ee cells (Miguel-Aliaga, 2012; Song et al., 2014) with largely unknown functions. Recent work has demonstrated nutrient-sensing roles of ee cells (Song et al., 2014, 2017).

The role of Bursicon/DLgr2 signaling has long been restricted to insect development, where the heterodimeric form of the hormone Bursicon, made by α and β subunits, is produced by a subset of neurons within the CNS during the late pupal stage and released systemically to activate its receptor DLgr2 in peripheral tissues to drive post-molting sclerotization of the cuticle and wing expansion (Baker and Truman, 2002; Luo et al., 2005; Mendive et al., 2005). We recently demonstrated a post-developmental activity for the α subunit of Bursicon (Bursα), which is produced by a subpopulation of ee cells in the posterior midgut, where it paracrinally activates DLgr2 in the visceral muscle (VM) to maintain homeostatic intestinal stem cell (ISC) quiescence (Scopelliti et al., 2014, 2016).

Here, we report an unprecedented systemic role for Bursα regulating adult energy homeostasis. Our work identifies a novel gut/fat body axis, where ee cells orchestrate organismal metabolic homeostasis. Bursα is systemically secreted by ee cells

© 2018 The Author(s). Published by Elsevier Inc.
This is an open access article under the CC BY license (http://creativecommons.org/licenses/by/4.0/).

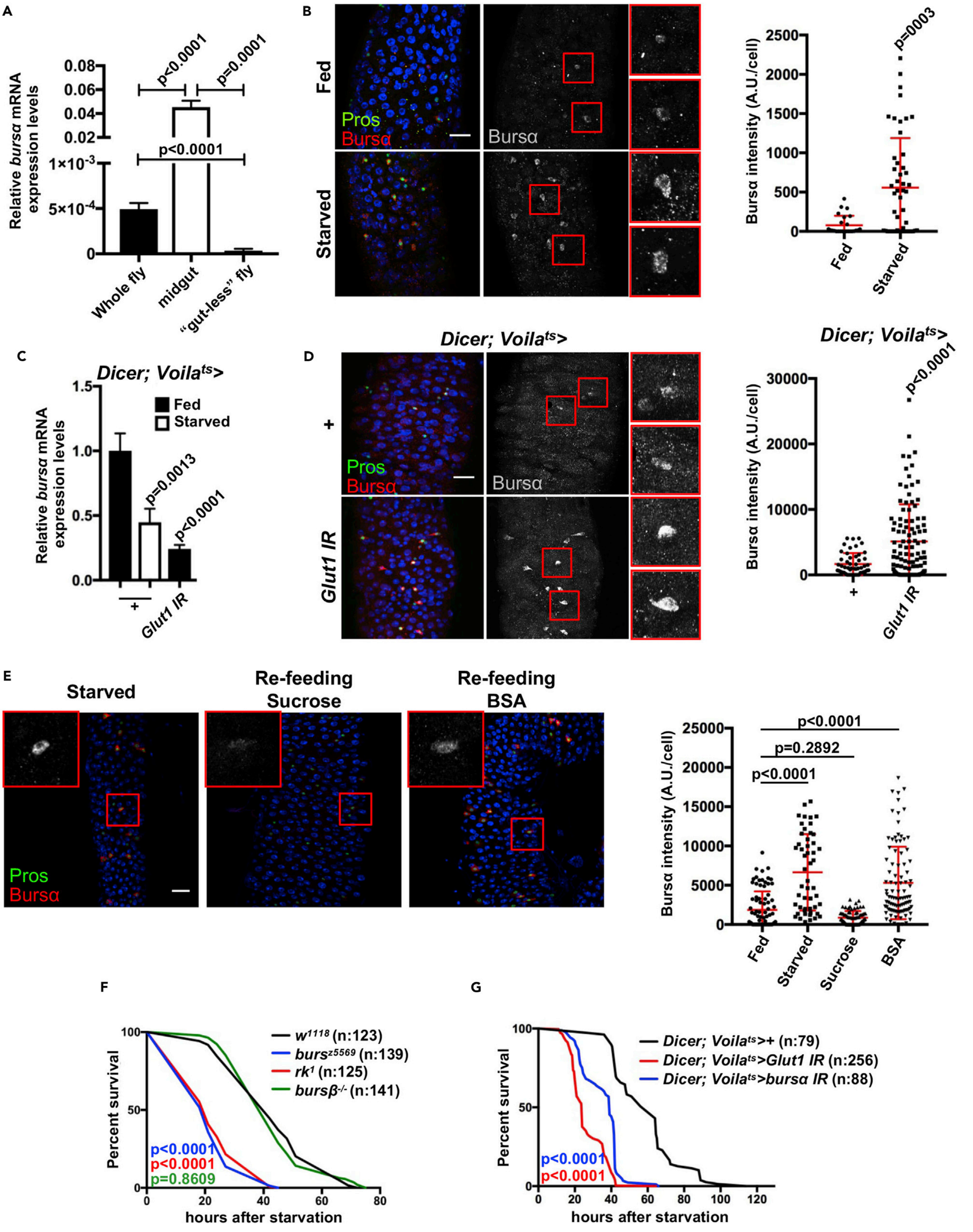

(legend on next page)

in response to nutrient availability and acts through DLgr2^{+} neurons to repress adipokinetic hormone (AKH)/AKH receptor (AKHR) signaling within the fat body/adipose tissue to restrict the use of energy stores. Impairment of systemic Bursα/DLgr2 signaling results in exacerbated oxidative metabolism, strong lipodystrophy, and organismal hypersensitivity to nutrient deprivation. Our work reveals a central role for ee cells in sensing organismal nutritional status and maintaining systemic metabolic homeostasis through coordination of an intestinal/neuronal/adipose tissue-signaling network.

RESULTS

Enteroendocrine Bursα Is Regulated in Response to Nutrient Availability

Ee cells are major sensors of luminal content (Engelstoft et al., 2008; Moran-Ramos et al., 2012) and coordinate gastrointestinal and systemic responses through secretory programs (Steinert and Beglinger, 2011) that affect gut motility, digestion, appetite, glucose homeostasis, and energy expenditure (Campbell and Drucker, 2013; Field et al., 2010; Gribble and Reimann, 2016; Park et al., 2016; Worthington et al., 2017; Zietek and Daniel, 2015). Our previous work revealed a local role for ee-derived Bursα in the adult midgut, which was necessary and sufficient to prevent ISC proliferation (Scopelliti et al., 2014, 2016). Knocking down *bursα* from ee cells resulted in ISC hyperproliferation in the normally quiescent homeostatic adult midgut, while *bursα* overexpression suppressed the characteristic proliferative response of ISCs following damage and upon aging (Scopelliti et al., 2014, 2016). In such a context, Bursα appears to have a permissive role in the maintenance of ISC quiescence. We next sought to identify conditions leading to an inducible function of Bursα.

First, to unambiguously assess the main source of Bursα production during adulthood, we compared mRNA expression levels in mature whole adults, adult midguts, and adult animals from which the gut was removed prior to RNA extraction ("gutless"). Confirming our previous results (Scopelliti et al., 2014, 2016) and subsequent independent reports (Chen et al., 2016; Dutta et al., 2015), we observed strong enrichment of *bursα* transcripts in adult midguts (Figure 1A).

Given our previous reports showing a local role of Bursα in controlling ISC proliferation (Scopelliti et al., 2014, 2016) and nutrients being key regulators of the proliferative state of the adult *Drosophila* midgut (O'Brien et al., 2011; Obata et al., 2018; Shim et al., 2013), we next explored the possibility that Bursα might be regulated by nutrients. We performed immunofluorescence staining on posterior midguts from animals fed *ad libitum* and following caloric deprivation. After 24 hr of complete, non-dehydrating starvation, ee cells showed increased immunoreactivity for Bursα compared with *ad libitum*-fed animals (Figure 1B). Interestingly, increased Bursα immunoreactivity in ee cells inversely correlated with transcript levels in the midgut (Figure 1C).

Consistent with previous reports indicating the death of Bursicon-producing neurons after adult eclosion (Honegger et al., 2011), Bursα immunostaining was undetectable in the CNS of adult animals independently of their feeding status (Figure S1A) and no effect was observed on adult *bursα* transcript levels beyond the midgut (Figure S1B). Therefore, our results suggest that ee-derived Bursα is responsive to nutrient availability.

Ee cells sense luminal content by expressing several chemoreceptors and transporters on their apical membrane. The mammalian low-affinity glucose transporter solute carrier family 2 member 2 (SLC2A2) is highly expressed on the surface of K and L ee cells, where it regulates the post-prandial secretion of the gastric inhibitory polypeptide (Cani et al., 2007). We next suppressed the expression of *Glut1*, the closest *Drosophila* homolog of SLC2A2, within adult ee cells of fully fed animals using a temperature-controlled *Voila*-Gal4 driver and RNA interference (IR) (*Voila^{ts}*>*Glut1* IR) (Figure S1C). Similarly to what we observed upon starvation, Bursα immunoreactivity was significantly increased within ee cells subjected to *Glut1* knockdown (Figure 1D), while *bursα* mRNA expression was downregulated (Figure 1C). These results suggest that sugars may be some of the key nutrients sensed by ee cells leading to regulation of Bursα in the midgut.

To further identify specific dietary factors that may affect Bursα levels within ee cells, we overexpressed *bursα* under the control of the temperature-sensitive *Voila*-Gal4 driver and subjected animals to starvation followed by refeeding with sucrose or BSA, as exclusive sources of sugar and protein, respectively. Importantly, the observed regulation of endogenous Bursα upon starvation (Figure 1B) was preserved with the overexpressed protein (Figure S1D). Interestingly, refeeding with sucrose, but not BSA, reverted Bursα to levels similar to the ones observed in fully fed conditions (Figure 1E), suggesting that ee-derived Bursα is primarily responsive to dietary sugars.

Figure 1. Bursα in ee Cells Is Regulated by Nutrients

(A and C) Transcript levels of *bursα* relative to *rpl32* in indicated tissue samples from 10- to 14-day-old adult wild-type animals (A) or whole midguts from animals of the indicated genotypes (C). Data represent the average of three biological replicates. Statistical analysis was done by unpaired t test (A) and one-way ANOVA followed by Tukey's multiple comparisons test (C). Bars represent mean ± SEM.

(B, D, and E) Immunostaining for Bursα (red/gray) and quantifications of Bursα fluorescent intensity in adult posterior midguts from 10- to 14-day-old control animals fully fed and upon 24 hr starvation (B), following ee-specific *Glut1* knockdown for 10–14 days (D) and upon *bursα* overexpression for 10–14 days under the indicated conditions (E). Prospero (Pros, green) labels ee cells. DAPI (blue) was used to stain all cell nuclei. Unless otherwise indicated, scale bars represent 20 μm. Red boxes in (B), (D), and (E) indicate areas magnified in far-right panels (B and D) and insets (E). Data are representative of observations made in at least two independent experiments with a minimum of eight midguts per genotype per condition each. Each dot in the graph (n) corresponds to an individual ee cell. n values: (B) = 25 (fed), 50 (starved); (D) = 51 (+), 105 (*Glut1 IR*); (E) = 84 (fed), 51 (starved), 74 (Suc), 96 (BSA). Statistical analysis was done through unpaired t test (B) and one-way ANOVA followed by Tukey's multiple comparisons test (D and E). Bars represent mean ± SEM.

(F and G) Survival upon starvation in flies of the indicated genotypes. Mutant animals were fed *ad libitum* for 7 days before being subjected to starvation (F) and transgenes for adult-specific knockdown animals were induced for 10 days prior to starting the starvation sensitivity test (G). Survival curves were analyzed using curve comparison and Log rank (Mantel-Cox) test. The number of animals assessed (n) is indicated in the graphs.

See also Figure S1.

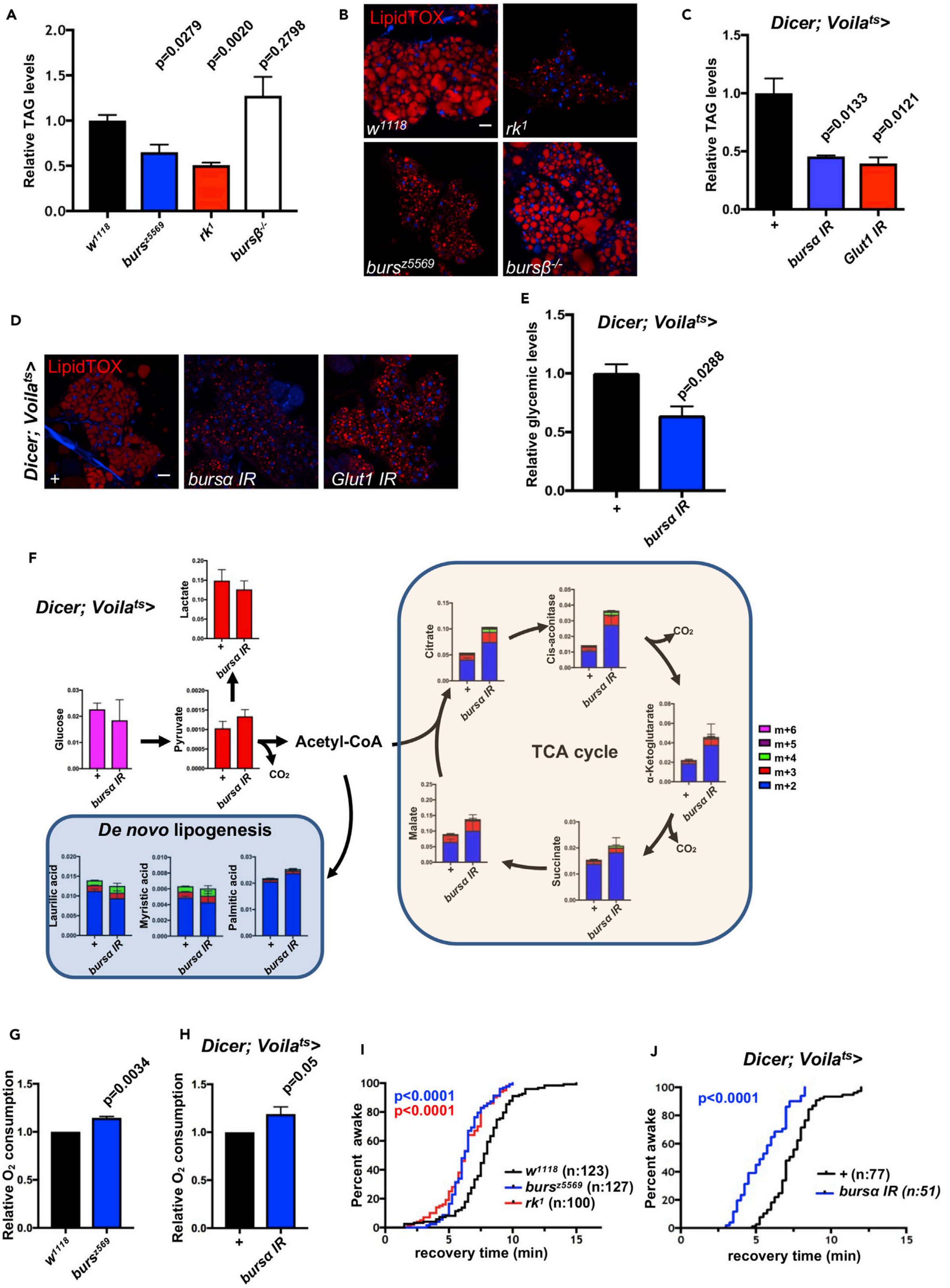

(legend on next page)

Altogether, these results indicate that, as part of their nutrient-sensing role, ee cells regulate Bursα, which is increased upon nutrient restriction.

Adult Bursα/DLgr2 Signaling Regulates Organismal Resistance to Metabolic Challenges

To begin addressing the physiological meaning of nutrient-dependent regulation of Bursα, we next assessed the role of Bursα/DLgr2 signaling in the organismal response to starvation. We performed survival analysis upon complete, non-dehydrating nutrient deprivation on whole mutants for *bursα* or its receptor *dlgr2/rk*. Adults were allowed to feed *ad libitum* for 7 days before being transferred to agar-only medium. Strikingly, *dlgr2* and *bursα* mutants showed a marked hypersensitivity to food deprivation compared with age-matched wild-type controls (w^{1118}), resulting in their reduced overall and median survival (Figure 1F).

We have previously demonstrated that the *bursβ* subunit is not expressed in the adult midgut and that it is dispensable for tissue homeostasis (Scopelliti et al., 2014, 2016). Consistently, null mutant animals for the *bursβ* subunit (*Df(2)110/Df(2)Exel6035*, thereafter $burs\beta^{-/-}$) did not display starvation sensitivity (Figure 1F). Therefore, Bursβ does not play a significant role in post-developmental functions so far revealed for Bursα and DLgr2 (Scopelliti et al., 2014, 2016). Importantly, since $burs\beta^{-/-}$ animals share the developmental defects of *dlgr2* and *bursα* mutants, it is unlikely that the starvation sensitivity observed in the latter two genotypes is a consequence of defective development.

To unambiguously demonstrate a post-developmental role of Bursα/DLgr2 in the response to nutrient deprivation, we suppressed *bursα* expression within adult ee cells by RNAi ($Voila^{ts}$>*bursα* IR). Similar to what we observed in whole mutant animals, targeted *bursα* knockdown induced a clear reduction in both median and overall survival following total caloric deprivation (Figure 1G). Consistently, animals bearing adult ee-restricted *Glut1* knockdown were also hypersensitive to starvation (Figure 1G).

Altogether, these results support an adult-specific, nutrient responsive role of Bursα/DLgr2 signaling that is necessary to sustain organismal survival upon nutrient deprivation.

Bursα/DLgr2 Regulates Adult Metabolic Homeostasis

The capacity of animals to withstand periods of scarce nutrients directly correlates with their accessibility to energy resources mainly stored as triacylglycerides (TAGs) in the fat body. Animals with excess fat body TAG are resistant to starvation (Bharucha et al., 2008), while reduced fat body TAG content results in hypersensitivity to starvation (Zhao and Karpac, 2017).

We therefore extended our investigation into the metabolic status of Bursα/DLgr2-deficient animals by assessing their energy stores. Consistently, we detected a significant overall reduction in the content of stored lipids, as indicated by decreased whole-body TAG levels in *bursα* and *dlgr2* mutants but not in $burs\beta^{-/-}$ animals (Figure 2A). Fat body staining with the lipophilic dye LipidTOX, to directly visualize TAG content (Junger et al., 2003), also revealed significant reduction of lipid droplet size exclusively in *bursα* and *dlgr2* mutant animals (Figure 2B). Importantly, adult ee-specific knockdown of *bursα* or *Glut1* resulted in a lipodystrophic phenotype similar to the ones observed in *bursα and dlgr2* mutants (Figures 2C, 2D, and S1E).

Following ingestion and absorption by the intestine, excess nutrients are stored in the fat body as TAG. The drastic reduction in energetic reserves upon defective Bursα/DLgr2 signaling prompted us to assess potential impairments in feeding and nutrient absorption as causative factors of the metabolic defects observed in these animals. Firstly, we quantified food intake in *bursα* mutants as well as in $Voila^{ts}$>*bursα* IR animals. Surprisingly, Bursα impairment resulted in a significant increase in food intake (Figures S2A and S2B). Therefore, the lean phenotype of Bursα-deficient animals is not due to reduced nutrient supply. Hyperphagia, however, can be a compensatory reaction to defects in the ability to efficiently absorb nutrients. To test this hypothesis, we quantified glucose absorption by pulse feeding the non-metabolizable fluorescent glucose analog 2-NBDG and measured its tissue accumulation. Bursα-impaired animals showed no reduction in 2-NBDG fluorescent signal when compared with controls (Figures S2C and S2D), suggesting that glucose absorption is not impaired.

We next quantified the undigested nutrients in the excreta of $Voila^{ts}$>*bursα* IR flies. We observed no significant differences in the levels of excreted glucose (Figure S2E), TAG (Figure S2F), or free fatty acids (FFA) (Figure S2G) in the knockdown animals. Therefore, our data suggest that *bursα* knockdown does not affect the normal digestive and absorptive functions of the gut.

Dietary nutrients are absorbed and processed by enterocytes and released into the hemolymph for uptake into peripheral organs. We therefore assessed the possibility that the defect in lipid storage observed in animals with compromised Bursα/DLgr2 signaling may arise from defective dietary lipid processing and transport by the enterocytes or impaired uptake of nutrients from the circulation by peripheral tissues.

Figure 2. Bursα/DLgr2 Signaling Maintains Metabolic Homeostasis

(A and C) Relative TAG levels from 7-day-old whole mutant animals (A) or animals following 14-day adult-specific transgene activation (C). Five females were collected for each biological replicate. Data for each genotype represent the mean of three biological replicates ±SEM. Statistical analysis was done by unpaired t test.

(B and D) LipidTOX staining (red) of dissected adult fat bodies from animals as in (A) and (C).

(E) Circulating glucose levels of animals subjected to 14-day adult-specific transgene activation. Data for each genotype represent the mean of three biological replicates ±SEM. Statistical analysis was done through unpaired t test.

(F) Whole-animal metabolomic analysis of heavy carbon incorporation after 6-hr $^{13}C_6$-D-glucose feeding following 7-day transgene activation. Three whole animals where used per biological replicate. Data for each genotype represent the mean of four biological replicates ±SEM.

(G and H) Relative O_2consumption of whole-fly mitochondrial extracts from 3-day-old whole mutant animals (G) or animals following 3-day adult-specific transgene activation (H). Data represent the average of three biological replicates. Statistical analysis was done by paired t test.

(I and J) Chill coma recovery time was recorded in 3-day-old mutants (I) or in animals following 3-day adult-specific transgene activation (J). Total number of animals assessed (n) is indicated within the graphs. Log rank (Mantel-Cox) test was used to analyze statistical significance.

See also Figure S2.

Defective lipid absorption or assembly and transport within enterocytes would result in reduced circulating lipid levels (hypolipidemia), while defective uptake of nutrients by peripheral tissues would result in hyperglycemia and hyperlipidemia.

We therefore collected hemolymph from Bursα/DLgr2-impaired adult animals and measured circulating glucose and fatty acid levels. Unexpectedly, we observed a prominent hypoglycemia (Figures 2E and S2H) and no defects in circulating TAG and FFA in these animals (Figures S2I and S2J). Therefore, scenarios of compromised enterocyte function or uptake of circulating nutrients by peripheral tissues are unlikely to be the case in Bursα/DLgr2-compromised animals.

Carbohydrates are the main components of the *Drosophila* diet and appear to be the preferred source of nutrients sensed by Bursα$^+$ ee cells (Figure 1E). Acetyl-coenzyme A (CoA) derived from glucose is metabolized within the mitochondria through the tricarboxylic acid (TCA) cycle and oxidative phosphorylation (OXPHOS) to generate energy in the form of ATP at the expense of O_2 molecules. Alternatively, glucose-derived acetyl-CoA is used as the substrate for *de novo* lipid synthesis, which occurs mainly in the fat body (Lee and Park, 2004; Mattila and Hietakangas, 2017; Musselman et al., 2013; Zhao and Karpac, 2017).

Therefore, we next traced glucose metabolism as a means to achieve a more comprehensive understanding of the metabolic phenotype of Bursα/DLgr2-compromised animals. We fed flies with uniformly heavy labeled $^{13}C_6$-D-glucose for 6 hr and tracked whole-body incorporation of glucose-derived heavy carbons into metabolites by liquid chromatography mass spectrometry. We found that knockdown of *bursα* resulted in overall increased incorporation of glucose-derived ^{13}C into metabolites of the TCA cycle (Figure 2F). Consistently, we observed increased mitochondrial O_2 consumption in *bursα* knockdown and whole mutant animals (Figures 2G and 2H). These data are indicative of increased mitochondrial respiration; i.e., increased utilization of glucose and O_2 to support the TCA cycle and OXPHOS to generate energy. Importantly, analysis of glucose-derived ^{13}C incorporation into fatty acids revealed normal *de novo* lipid synthesis (Figure 2F), suggesting that the reduced TAG levels in *bursα* knockdown animals are a consequence of increased lipolysis rather than defective lipid synthesis.

We next monitored whether increased physical activity of Bursα/DLgr2-deficient animals might explain their higher rate of oxidative metabolism. Video tracking of fully fed *Voila^{ts}>bursα* IR animals and whole mutants for *bursα* and *dlgr2* showed either normal or reduced locomotor activity, respectively (Figures S2K and S2L). The reduced motility of whole mutant animals is not surprising given their severe wing and leg developmental defects (Luo et al., 2005; Peabody et al., 2008). Altogether, our data suggest that the metabolic imbalance observed in Bursα/DLgr2-impaired animals is unlikely to be caused by poor feeding, defective nutrient absorption, or impaired *de novo* lipid synthesis, but it is rather the result of increased oxidative metabolism. Furthermore, increased energy production is not offset by increased locomotion.

Increased metabolic rate is associated with increased thermogenesis (Hulbert et al., 2004; Moraru et al., 2017). Consistently, *bursα* knockdown and whole mutant animals showed quicker recovery after chill coma, an indirect readout of increased body-heat production (Moraru et al., 2017) (Figures 2I and 2J). Therefore, our results identified a crucial role for adult Bursα/DLgr2 signaling regulating systemic metabolic homeostasis through regulation of OXPHOS and maintenance of organismal energy stores.

Bursα Acts as a Systemic Mediator of Metabolic Homeostasis

We have previously demonstrated a local role of Bursα/DLgr2 signaling in the adult midgut in which ee-derived Bursα restrains epidermal growth factor-mediated ISC proliferation through paracrine activation of DLgr2 in the midgut VM (Figure S3A) (Scopelliti et al., 2014). We therefore asked whether the same gut-intrinsic signaling was responsible for the observed metabolic functions of Bursα/DLgr2.

To address this hypothesis, we suppressed midgut Bursα/DLgr2 signaling through targeted knockdown of the receptor in the adult VM using a temperature-sensitive *how*-Gal4 driver. Unexpectedly, targeted knockdown of *dlgr2* from the VM did not recapitulate the increased starvation sensitivity (Figure S3B), hypoglycemia (Figure S3C), or reduction in TAG content (Figure S3D) observed in Bursα/DLgr2-deficient animals. Most importantly, targeted overexpression of a wild-type form of *dlgr2* within the VM failed to rescue the metabolic defects of *dlgr2* mutant animals (Figure S3E). Therefore, the metabolic dysfunction observed in response to Bursα/DLgr2 impairment is independent of the paracrine activation of the signaling in the adult midgut.

Our data also imply that, in addition to its role as an ISC niche factor, ee-derived Bursα may act in an endocrine fashion to exert systemic roles. To formally assess our hypothesis, we performed western blot analysis for Bursα on hemolymph from wild-type animals fed *ad libitum* and following 24-hr starvation. We observed a significant reduction in circulating Bursα levels in animals subjected to starvation, compared with fully fed counterparts (Figures 3A and S3F). Critically, adult ee-targeted knockdown of *bursα* significantly reduced the hormone detectable in the hemolymph, coupling Bursα production within ee cells to its circulating levels (Figures 3B and S3G). Therefore, the regulation of systemic Bursα secretion in response to nutrients is likely to be an essential aspect of the hormone's role in the regulation of adult metabolic homeostasis. Consistently, fully fed animals subjected to direct impairment of Bursα secretion from ee cells by overexpressing oxysterol-binding protein, known to cause hormone retention within the Golgi complex (Ma et al., 2012), mimic the systemic metabolic phenotype of *bursα*- and *dlgr2*-deficient animals (Figures S3H and S3I).

Altogether, these results demonstrate that ee cells secrete Bursα into circulation in response to nutrients and that the increased Bursα immunoreactivity observed in midgut ee cells from starved animals (Figure 1B) reflects hormone retention within the gut upon starvation. Therefore, in addition to its gut-intrinsic role on ISC homeostasis, Bursα exerts endocrine functions that affect systemic metabolic homeostasis in adult animals.

Bursα Mediates a Gut-Neuronal Relay Regulating Metabolic Homeostasis

The *Drosophila* fat body is the functional homolog of the mammalian adipose tissue and liver (Gutierrez et al., 2007) and

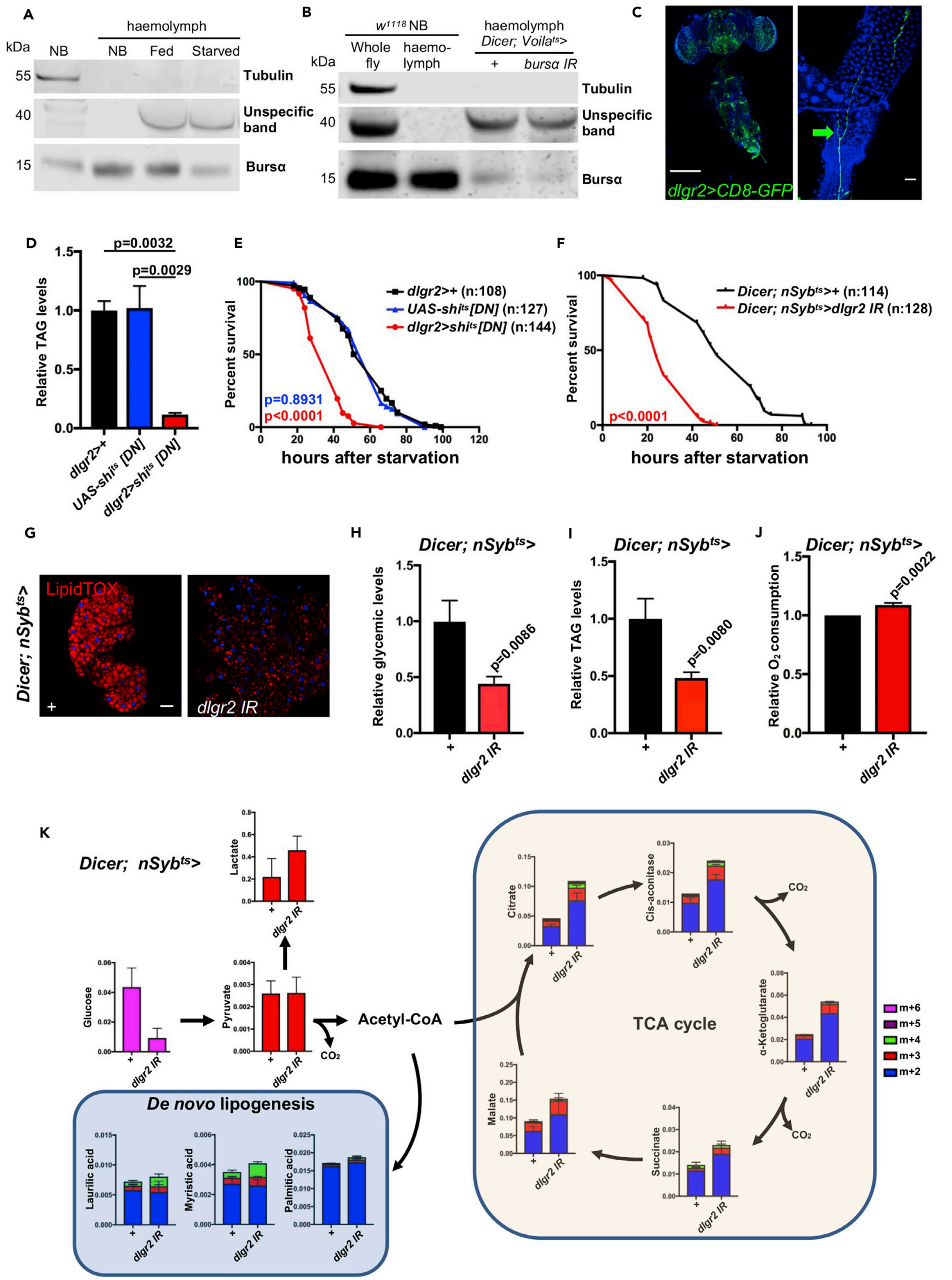

(legend on next page)

the main site of synthesis, storage, and mobilization of lipid reserves upon organismal energetic demands (Musselman et al., 2013; Zhao and Karpac, 2017).

Direct gut to fat body signaling regulates a systemic response to high-caloric diet (Song et al., 2017). We therefore asked whether a similar gut-adipose tissue axis was responsible for the control of metabolic homeostasis by Bursα/DLgr2 signaling.

We first assessed *dlgr2* expression in fat body cells by expressing the nuclear reporter RedStinger under the control of a *dlgr2*-Gal4 driver. Immunofluorescence analysis revealed absence of nuclear signal within fat body cells (Figure S3J). To exclude incomplete endogenous gene expression pattern by our *dlgr2*-Gal4 reporter, we suppressed *dlgr2* expression within adult fat body cells by overexpressing a *dlgr2* IR specifically under the control of the temperature-sensitive fat body driver *Lsp2*-Gal4 and assessed animal survival upon starvation and lipid storage levels. Consistent with the *dlgr2*-Gal4 reporter expression pattern, targeted fat body knockdown of *dlgr2* did not recapitulate the full extent of phenotypes of *bursα*-compromised animals (Figures S3K and S3L), supporting the lack of DLgr2 functionality within fat body cells. Moreover, overexpression of wild-type *dlgr2* within the fat body showed no rescue of the lipid metabolic phenotype of *dlgr2* mutants (Figure S3O). Overall, these data pointed to an indirect action of Bursα on fat body cells.

Interestingly, our immunofluorescence mapping of *dlgr2* expression showed reporter positivity in terminal tracheal cells in close proximity to fat body tissue (Figure S3J). To assess a potential contribution of those cells to the metabolic phenotype of *bursα* and *dlgr2* mutant animals, we suppressed *dlgr2* expression in adult terminal tracheal cells using a temperature-controlled *dsrf*-Gal4 driver. However, we did not observe drastic changes in the ability of the knockdown animals to survive after food deprivation or in their systemic TAG content when fed *ad libitum* (Figures S3M and S3N). Furthermore, terminal-tracheal-specific *dlgr2* re-expression was not able to revert the low-TAG phenotype of *dlgr2* mutants (Figure S3O), suggesting that circulating Bursα is unlikely to exert its systemic metabolic activity by directly binding DLgr2 in fat body or terminal tracheal cells.

We next assessed other adult organs for *dlgr2* expression. We observed strong *dlgr2*-Gal4 reporter activity throughout the CNS and within enteric neurons innervating the R5 region of the posterior midgut (Figure 3C). We therefore hypothesized that Bursα could control systemic metabolism through activation of neuronal DLgr2. Indeed, impairing the synaptic activity of $DLgr2^+$ neurons by overexpressing a dominant-negative, temperature-sensitive form of the dynamin-like protein *shibire* (*shi*) led to a clear reduction of whole-body TAG levels and organismal capacity to survive starvation (Figures 3D and 3E).

Therefore, we next suppressed *dlgr2* expression pan-neuronally in adult flies using a temperature-controlled *nSyb*-Gal4 driver to overexpress *dlgr2* IR. As observed for whole mutants and ee-targeted *bursα* knockdown animals, adult-restricted neuronal suppression of *dlgr2* expression induced a striking reduction in the capacity of these animals to survive starvation (Figure 3F) and recapitulated all the metabolic hallmarks observed in *bursα* and *dlgr2* mutants and $Voila^{ts}$>*bursα* IR animals (Figures 3G–3K), including hyperphagic behavior (Figure S4A), normal nutrient absorption (Figures S4B–S4D), enhanced glucose oxidation (Figures 3J and 3K), and faster recovery time from chill coma (Figure S4E). Concomitant targeted knockdown of neuronal *dlgr2* and ee *bursα* showed no additive effects on the metabolic phenotypes observed in the individual knockdowns (Figures 4A and 4B), indicating that ee-derived ligand and neuronal receptor work within the same pathway to regulate systemic metabolic homeostasis.

Importantly, pan-neuronal overexpression of wild-type *dlgr2* significantly reverted the low TAG levels, fat body lipid droplet defects, and starvation sensitivity of *dlgr2* mutants (Figures 4C–4E). Gut to brain signaling includes the translation of luminal cues sensed by ee cells and involves a neuronal relay including enteric neurons (Mayer, 2011). Although the possibility of a direct communication between ee cells and enteric neurons is appealing, we have so far been unable to identify the subpopulation of $DLgr2^+$ neurons responsible for the metabolic functions of this signaling pathway, precluding targeted knockdown experiments within a more restricted neuronal subpopulation.

Figure 3. Systemic Bursα Acts on Neuronal DLgr2 to Maintain Organismal Metabolic Homeostasis

(A and B) Western blot analysis of hemolymph samples from 10- to 14-day-old wild-type animals fully fed or following 24-hr starvation (A) or fully fed animals bearing ee-specific *bursα* knockdown for 14 days (B). Far left lanes in (A) and (B) depict whole-animal samples. NB, newborn. Data are representative of three independent experiments.

(C) Immunofluorescence of CNS (left) and enteric neurons innervating the posterior end of the R5 region of the midgut and hindgut (right) in adult flies expressing a *CD8-GFP* reporter under *dlgr2*-Gal4. Arrow points to enteric neuronal terminals. Scale bar, 200 μm (left) and 20 μm (right).

(D and I) Relative TAG levels in animals following 14-day adult-specific transgene activation. Five females were collected for each biological replicate. Data for each genotype represent the mean of three biological replicates ± SEM. Statistical analysis was done by one-way ANOVA followed by Tukey's multiple comparisons test (D) and unpaired t test (I).

(E and F) Survival upon starvation in flies of the indicated genotypes subjected to 10-day transgene activation prior to the start of the starvation test. Total number of animals assessed (n) is indicated within the graphs. Log rank (Mantel-Cox) test was used to analyze statistical significance.

(G) LipidTOX staining (red) in fat bodies from animals following 14-day adult-specific transgene activation.

(H) Relative circulating glucose levels in animals following 14-day adult-specific transgene activation. Data for each genotype represent the mean of three biological replicates ±SEM. Statistical analysis was done through unpaired t test.

(J) Relative O_2 consumption of whole-fly mitochondrial extracts from animals following 3-day adult-specific transgene activation. Four whole animals where used per biological replicate. Data for each genotype represent the mean of four biological replicates ± SEM. Statistical analysis was done through paired t test.

(K) Whole-animal metabolomic analysis of heavy carbon incorporation after 6-hr $^{13}C_6$-D-glucose feeding following 7-day transgene activation. Three whole animals were used per biological replicate. Data for each genotype represent the mean of four biological replicates ±SEM.

See also Figures S3 and S4.

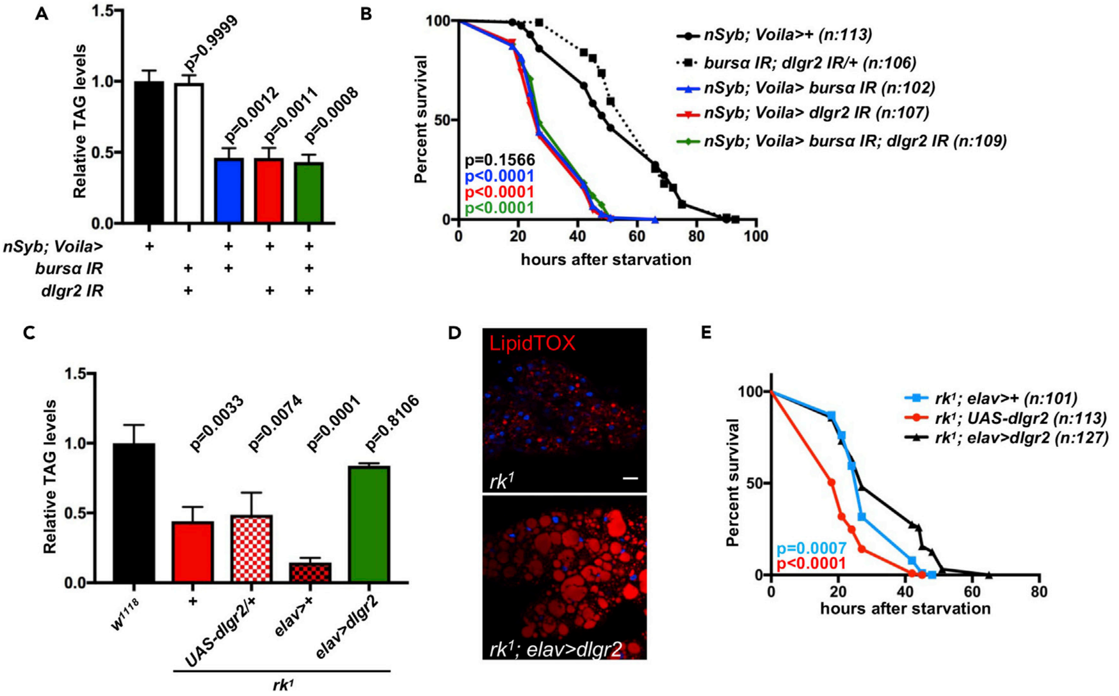

Figure 4. Enteroendocrine Bursα and Neuronal DLgr2 Work within the Same Pathway to Regulate Systemic Metabolic Homeostasis

Relative whole-body TAG levels (A and C), survival upon starvation (B and E), and fat body LipidTOX staining (D) from adults of the indicated genotypes, following 7-day transgene expression.

(A and C) Five females were collected for each biological replicate. Data for each genotype represent the mean of three biological replicates ± SEM. Statistical analysis was done by one-way ANOVA followed by Tukey's multiple comparisons test.

(B and E) Total number of animals assessed (n) is indicated within the graphs. Log rank (Mantel-Cox) test was used to analyze statistical significance.

See also Figure S4.

Overall, our data reveal a novel, nutrient-sensitive ee/neuronal/fat body network controlling systemic energy balance through endocrine Bursα/DLgr2 signaling.

Systemic Bursα Controls AKH Signaling

Metabolic homeostasis is regulated by complex hormonal systems. As in mammals, *Drosophila* insulin and glucagon-like AKH signaling are two counteracting key regulators of the balance between nutrient storage and usage, respectively. *Drosophila* insulin acts as a conserved satiety hormone promoting glucose uptake by peripheral tissues (Saltiel and Kahn, 2001) and sustaining sugar and lipid anabolic processes (Buch et al., 2008; Kim and Rulifson, 2004). AKH signaling, conversely, is activated in response to reduced nutrient availability and promotes mobilization of energy reserves (Bharucha et al., 2008; Kim and Rulifson, 2004; Lee and Park, 2004).

Eight *Drosophila* insulin-like peptides (DILPs 1–8) have been identified (Brogiolo et al., 2001; Gronke et al., 2010), seven of which activate a single insulin-like receptor (InR) to repress the activity of the transcription factor FoxO. DILP2, -3, and -5 are the main regulators of sugar homeostasis. They are produced by the pars intercerebralis of the brain and are responsive to nutritional status (Colombani et al., 2003; Geminard et al., 2009; Ikeya et al., 2002; Rulifson et al., 2002).

To test the involvement of insulin signaling in our system, we first assessed the transcriptional levels of *insulin receptor* (*InR*) and *thor* (*4ebp*), two known targets of the insulin-dependent transcription factor FoxO (Junger et al., 2003; Puig and Tjian, 2005) in the fat body of *Voila*^{ts}>*bursα* IR and *nSyb*^{ts}>*dlgr2* IR animals. Neither genetic context affected the transcriptional levels of those targets (Figure S5A). Furthermore, we did not detect significant variations in the transcript levels of *dilp2* in heads of *Voila*^{ts}>*bursα* IR and *nSyb*^{ts}>*dlgr2* IR animals (Figure S5B). Levels of *dilp3* transcripts were significantly reduced, while a trend of reduction was observed for *dilp5* (Figure S5B), consistent with previous reports showing transcriptional suppression of *dilp5* following reduced circulating sugars (Birse et al., 2011; Ikeya et al., 2002), a feature of Bursα/DLgr2-deficient animals (Figures 2E, 3H, and S2H).

Finally, we used the membrane-associated GFP fluorescent insulin signaling reporter line tGPH (Britton et al., 2002) to assess the levels of insulin signaling within fat body cells. Neuronal suppression of *dlgr2* expression did not induce variations in the levels of the reporter (Figure S5C). Overall, these data indicate that Bursα/DLgr2 signaling is unlikely to control metabolic homeostasis through the regulation of systemic insulin signaling.

Metabolic stresses, such as starvation, evoke systemic hormonal responses to mobilize energy stores. A critical inducer of energy mobilization in *Drosophila* is AKH (Bharucha et al., 2008; Galikova et al., 2015; Gronke et al., 2007), produced by the corpora cardiaca and homolog of the mammalian glucagon- and β-adrenergic signaling. Reduction of AKH signaling leads to

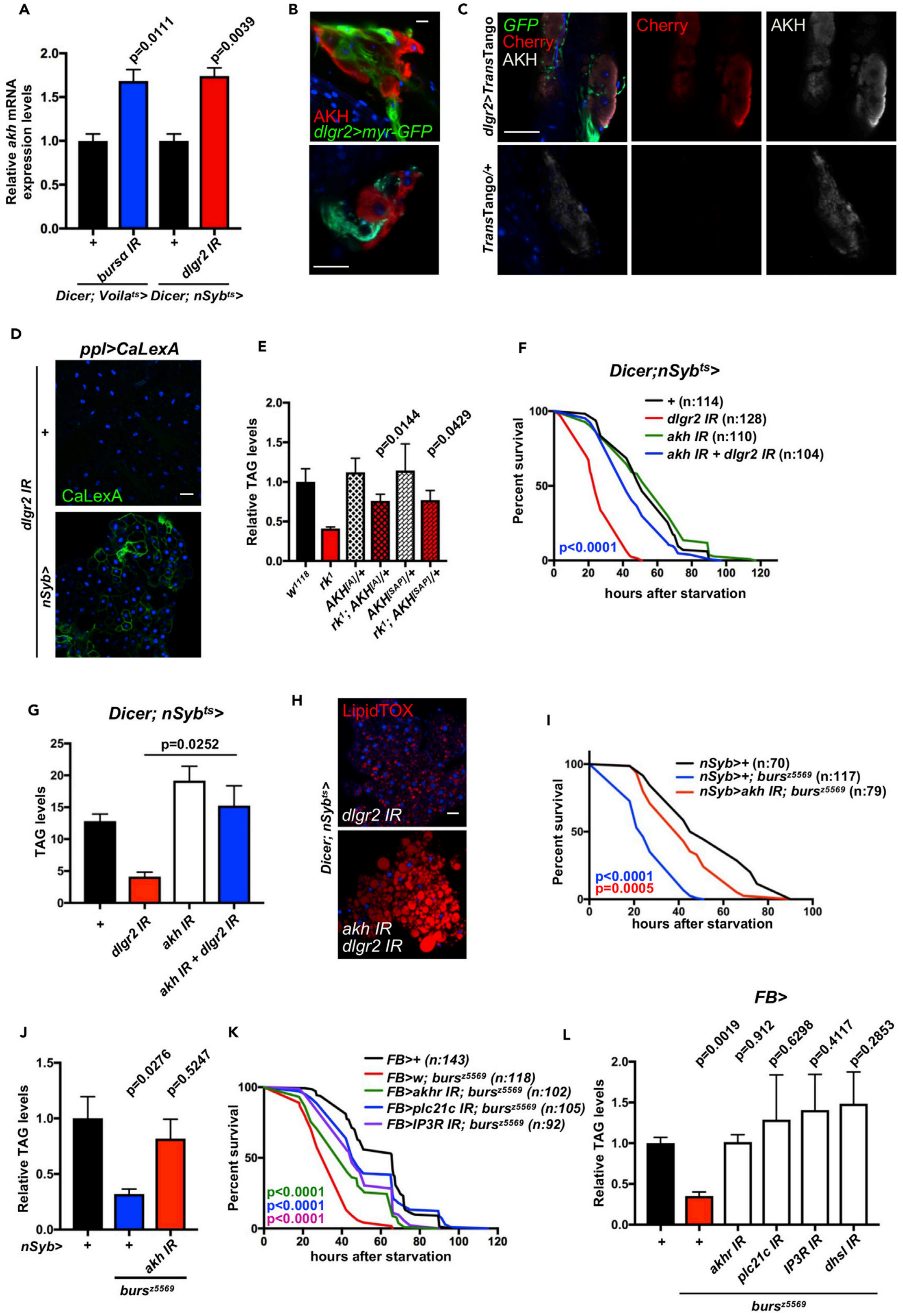

(legend on next page)

increased fat body lipid droplets and whole-body TAG, and organismal resistance to starvation (Bharucha et al., 2008).

Knocking down ee *bursα* or neuronal *dlgr2* results in increased expression of *akh* (Figure 5A), suggesting that Bursα/DLgr2 may exert metabolic functions through modulation of AKH signaling.

To gain further insight into the relationship between AKH/AKHR and Bursα/DLgr2 signaling, we next analyzed the anatomical location of AKH$^+$ and DLgr2$^+$ cells. Expression of the membrane tethered *myr-GFP* under the control of the *dlgr2*-Gal4 driver and immunostaining for AKH clearly shows two distinct and non-overlapping populations of cells (Figures 5B and S6A). Consistently, adult knockdown of *dlgr2* in AKH$^+$ cells does not affect animal survival upon nutrient deprivation (Figure S6B) or TAG levels (Figure S6C). Therefore, our data suggest that the crosstalk between Bursα/DLgr2 and AKH/AKHR signaling is not mediated through a direct action of DLgr2 within AKH-producing cells.

We next assessed whether DLgr2$^+$ neurons were capable of establishing synapses with AKH$^+$ neurons by using the *trans*-Tango unidirectional transsynaptic labeling system (Talay et al., 2017). We used *dlgr2*-Gal4 to drive a synthetic human glucagon ligand in presynaptic neurons. Synapses between two neuronal populations would result in the binding of glucagon to glucagon receptor, leading to the activation of a QF-driven, QUAS-mCherry reporter within post-synaptic neurons. Consistently, we observed *dlgr2-Gal4*-dependent activation of mCherry reporter expression within AKH$^+$ neurons (Figure 5C). Specificity of the signal was confirmed by the absence of mCherry reporter expression in tissues from animals lacking *dlgr2-Gal4* (Figure 5C). Altogether, these results suggest the existence of synaptic connections between DLgr2$^+$ and AKH$^+$ neurons. Overall, the level of post-synaptic reporter activation we observed appears significantly reduced when compared with reports on other neuronal synapses (Talay et al., 2017). This may reflect a low degree of connection between DLgr2 and AKH neurons. Functional interactions between both signaling systems may involve additional and indirect neuronal connections through neurotransmitters.

AKH signals to the fat body through its G-protein-coupled receptor AKHR. Upon ligand binding, AKHR evokes Ca^{2+} release from the ER stores into the cytosol (intracellular Ca^{2+} [iCa^{2+}]) (Bharucha et al., 2008) through a conserved signaling cascade including the membrane-associated phospholipase PLC21c and downstream production of 1,4,5-inositol trisphosphate (IP3). Free cytosolic IP3 increases cytosolic Ca^{2+} levels by activating the Ca^{2+} channel IP3 receptor (IP3R/ITP-R83A) on the ER membrane.

To directly visualize active fat body iCa^{2+} signaling following impairment of Bursα/DLgr2, we used the transcriptional NFAT-based cytoplasmic Ca biosensor CaLexA (Ca-dependent nuclear import of Lex A) (Masuyama et al., 2012). We imaged CaLexA-expressing fat bodies of flies, where neuronal *dlgr2* expression was suppressed. Strikingly, although a weak to undetectable signal was observed in control flies, reporter activity was readily detectable in fat bodies from animals with neuronal knockdown of *dlgr2* (Figure 5D). This result suggests a potential hyperactivation of AKHR signaling within the fat body following impairment of Bursα/DLgr2 activity. Consistently, *akh* heterozygote mutants significantly reverted the lipodystrophic phenotype of *dlgr2* mutant animals (Figure 5E).

We next used RNAi targeted knockdown to fully assess the functional connection between AKH/AKHR and Bursα/DLgr2 signaling in the regulation of systemic metabolic homeostasis. Adult-specific neuronal co-suppression of *dlgr2* and *akh* expression by RNAi rescued the starvation sensitivity and lipodystrophy of *dlgr2* knockdown animals (Figures 5F–5H and S6D–S6F). Furthermore, targeted *akh* knockdown significantly suppressed the metabolic phenotypes of *bursα* mutant animals (Figures 5I and 5J).

Complementary to the results obtained upon neuronal ligand knockdown, RNAi-dependent reduction of *akhr* expression within fat body cells using the fat body driver *FB*-Gal4 in a *bursα* mutant background improved the sensitivity to starvation and adiposity of *bursα* mutant animals (Figures 5K, 5L, and S6G–S6I).

Deregulated intracellular Ca homeostasis in adipocytes has been associated with severe lipodystrophy (Bi et al., 2014). Therefore, we reduced iCa^{2+} levels by suppressing fat body expression of *Plc21c* and *IP3R* in a *bursα* mutant background. Both manipulations were sufficient to improve survival upon starvation (Figure 5K) and suppress the lipodystrophic phenotype (Figures 5L and S6G–S6I) of *bursα* mutant animals.

AKH/AKHR signaling induces phosphorylation and activation of different substrates, resembling the action of β-adrenergic signaling in mammals (Beller et al., 2010; Kuhnlein,

Figure 5. Bursα/DLgr2 Signaling Regulates Metabolic Homeostasis through Attenuation of AKH/AKHR Pathway Activation

(A) *akh* mRNA levels in whole flies of the indicated genotypes following 14 days of transgene activation. Significances are shown relative to their individual controls. Data represent the average of three biological replicates. Statistical analysis was done by unpaired t test. Bars represent mean ± SEM.

(B) Immunofluorescent staining in flies expressing *myr-GFP* under the *dlgr2*-Gal4 driver (green) and co-stained with anti-AKH antibody (red).

(C) Immunofluorescent staining of synaptic connections between DLgr2$^+$ and AKH$^+$ neurons indicated by *dlgr2*-Gal4 (green)-dependent activation of mCherry reporter expression (red) within AKH$^+$ neurons (gray) using the *trans*-Tango unidirectional transsynaptic labeling system.

(D) Immunofluorescent staining of fat bodies following 14-day adult-specific transgene activation, including overexpression of the Ca sensor CaLexA (green).

(E, G, J, and L) TAG levels of 7-day-old animals of indicated genotypes (E; p values indicate comparison against rk^1 mutants), animals following 14-day adult-specific transgene activation (G), and 7-day-old control and whole mutant animals with or without concomitant expression of the indicated transgenes (J and L). Five females were collected for each biological replicate. Data for each genotype represent the mean of three biological replicates ±SEM. Statistical analysis was done by unpaired t test.

(F, I, and K) Survival upon starvation in animals of the indicated genotypes. Starvation sensitivity tests were started in animals following 10-day post-adult-specific transgene activation (F; p value indicates comparison against *dlgr2IR* animals) or 7-day-old control and whole mutant animals with or without concomitant expression of the indicated transgenes (I and K). Total number of animals assessed (n) is indicated within the graphs. Log-rank (Mantel-Cox) test was used to analyze statistical significance.

(H) LipidTOX staining (red) in adult fat bodies following 14-day adult-specific transgene activation.

See also Figures S5 and S6.

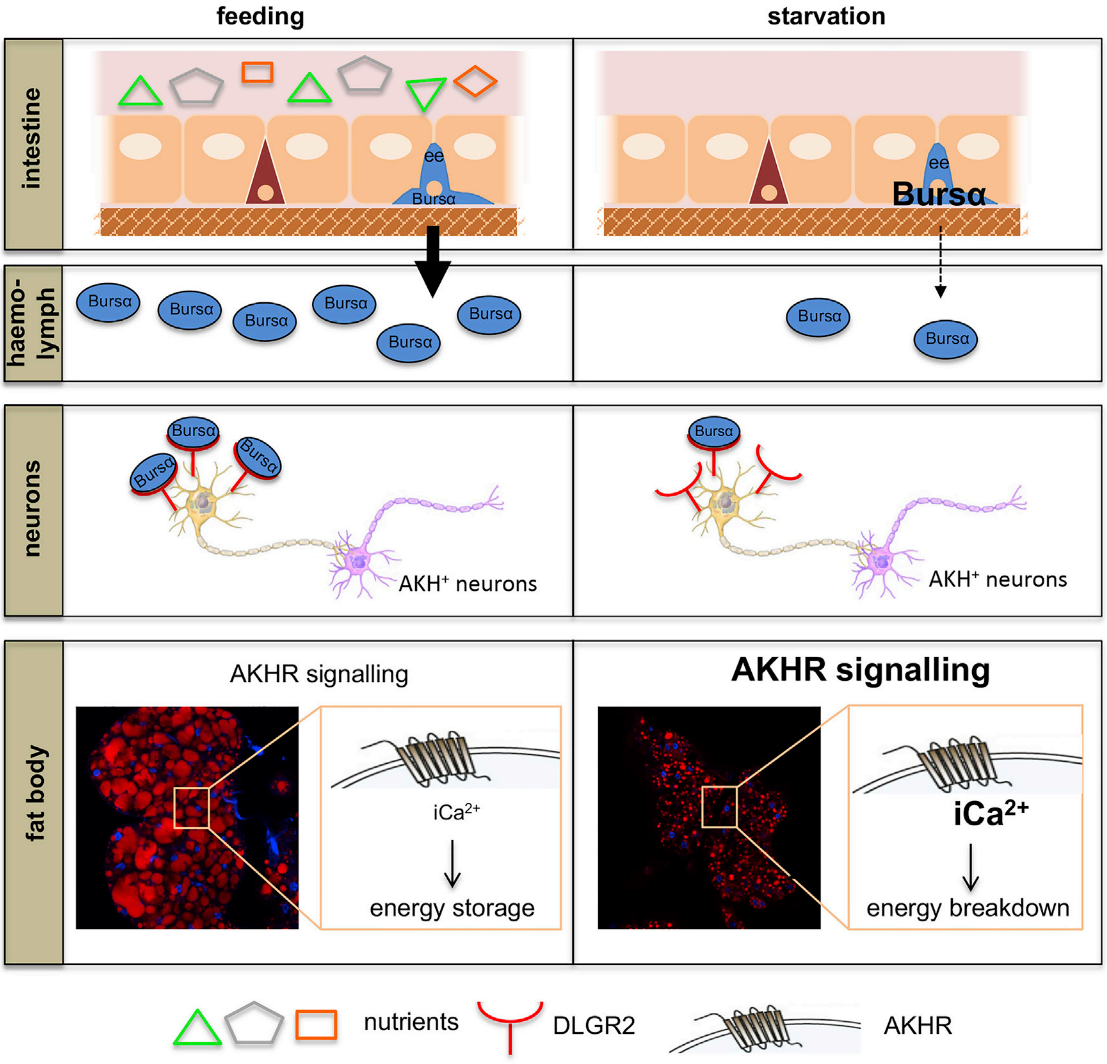

Figure 6. Working Model

Enteroendocrine cells respond to the presence of nutrients by secreting the hormone Bursicon α, which signals via its neuronal receptor DLgr2. Bursicon α/DLgr2 signaling regulates energy catabolism through a neuronal relay leading to the restriction of glucagon-like, AKH production and subsequent modulation of AKHR signaling within the fat body/adipose tissue. Impaired Bursicon α/DLgr2 signaling leads to exacerbated glucose oxidation and depletion of energy stores. Yellow and pink neuronal cells represent DLgr2$^+$ and AKH$^+$ neurons, respectively.

2011). One of such substrates is the hormone sensitive lipase (dHSL), which catalyzes the hydrolysis of both tri- and diacylglycerides along with cholesterol esters upon starvation (Holst et al., 1996).

To assess whether dHSL is an effector of lipolysis downstream of the Bursα/DLgr2/AKH/AKHR pathway, we suppressed *dhsl* expression in fat body cells of *bursα* mutant animals. This genetic manipulation was sufficient to rescue the TAG levels of Bursα/DLgr2-impaired animals (Figures 5L and S6G–S6I), suggesting that the activation of dHSL is at least in part responsible for the reduced adiposity observed in Bursα/DLgr2-compromised flies.

Altogether, our results reveal a novel inter-organ communication program involving a subpopulation of enteroendocrine cells able to respond to the presence of nutrients and signal to neurons in order to restrain catabolic programs and pro-lipolytic signals in the adipose tissue to maintain systemic energetic homeostasis (Figure 6).

DISCUSSION

Work in the mammalian and fly intestine suggest wide phenotypic and functional diversity of ee cells (Haber et al., 2017; Lebrun et al., 2017; Yan et al., 2017). A systematic molecular and functional characterization of ee cells is likely to explain the mechanisms behind the sophisticated functions of the intestine as a central endocrine, immune, and metabolic organ. Our work demonstrates the unique power of *Drosophila* as a paradigm to deconvolute such intricate processes, with long-reaching impact into physiological and pathogenic metabolism.

Nutrient Sensing by ee Cells and Systemic Release of Bursα

Here, we show that ee cells secrete Bursα in the presence of plentiful nutrients, while caloric deprivation reduces its systemic release and consequently results in hormone accumulation within ee cells. Interestingly, we observed that conditions leading to the latter scenario are accompanied by reduced *bursα* transcription. The reasons underlying the inverse correlation between midgut *bursα* mRNA and protein levels are unclear and may represent part of a negative feedback mechanism for ultimate control of further protein production. A similar phenomenon is described during the regulation of the secretion of other endocrine hormones, such as DILPs (Hasygar and Hietakangas, 2014; Koyama and Mirth, 2016).

Our results show that Bursα within ee cells is preferably regulated in response to dietary sugars. This is further supported by the function of Glut1 as at least one of the transmembrane sugar transporters connecting nutrient availability to Bursα signaling. Glut1 is the closest homolog of the mammalian regulator of ee incretin secretion SLC2A2, and it has been shown to positively regulate the secretion of peptide hormones in flies (Park et al., 2014). Whether Glut1 is a central sensor of dietary sugars and hormone secretion by ee cells remains to be addressed. However, it is likely that, in the face of challenges, such as starvation, multiple mechanisms of nutrient sensing and transport converge to allow a robust organismal adaptation to stressful environmental conditions.

Metabolic Functions of Bursα/DLgr2

Reduction of systemic Bursα/DLgr2 signaling induces a complex metabolic phenotype, characterized by lipodystrophy and hypoglycemia, which is accompanied by hyperphagia. These phenotypes are not due to poor nutrient absorption or uptake by tissues or impaired synthesis of energy stores but are rather a consequence of increased catabolism. This is supported by a higher rate of glucose-derived ^{13}C incorporation into TCA cycle intermediates, accompanied by increased mitochondrial respiration and body-heat production.

While our glucose tracing experiments help explain the hypoglycemic phenotype of Bursα/DLgr2-compromised animals even in the context of hyperphagia, they do not directly address the reduction in fat body TAG. The latter would require $^{13}C_6$-palmitate tracing for assessment of the rate of lipid oxidation and incorporation into the TCA cycle. This was precluded by overall poor uptake of $^{13}C_6$-palmitate into adult animals even after prolonged periods of feeding (data not shown). However, the depletion of fat body TAG stores in the presence of normal *de novo* lipid synthesis in Bursα/DLgr2-impaired animals strongly suggests that at least part of the increased rate of O_2 consumption in those animals results from increased lipid breakdown via mitochondrial fatty acid oxidation. Consistently, increased O_2 consumption rates and the thermogenic phenotype of Bursα/DLgr2-deficient animals are attenuated upon reduction of AKH/AKHR signaling (Figures S4E and S4F). Finally, the functional role of dHSL in the fat body further supports the regulation of lipid breakdown by AKH/AKHR signaling as at least one of the key aspects mediating the role of Bursα/DLgr2 signaling in adult metabolic homeostasis (Figure 6).

Local versus Systemic Functions of Intestinal Bursα

Our previous work revealed that ee Bursα is required to maintain homeostatic ISC quiescence in the adult *Drosophila* midgut; that is, in the midgut of unchallenged and well-fed animals (Scopelliti et al., 2014, 2016). Such a role of Bursα is mediated by local or short-range signaling through DLgr2 expressed within the midgut VM (Scopelliti et al., 2014). Here, we demonstrate a systemic role of Bursα that does not involve VM-derived DLgr2 but rather signals through its neuronal receptor. In that regard, the paracrine and endocrine functions of Bursα/DLgr2 are uncoupled. However, the regulation of ee-derived Bursα by nutrients is likely to affect local as well as systemic Bursα/DLgr2 signaling. Retention of Bursα within ee as observed in conditions of starvation may impair the hormone's signaling into the VM, which, in principle, would lead to ISC hyperproliferation (Scopelliti et al., 2014). In fact, under full nutrient conditions, genetic manipulations impairing systemic Bursα signaling, such as ee *Glut1* knockdown or *osbp* overexpression, lead to ISC hyperproliferation comparable with that observed upon *bursα* knockdown (Scopelliti et al., 2014) (Figure S6J). This represents an apparent conundrum, as ISC proliferation is not the expected scenario in the context of starvation (Choi et al., 2011; O'Brien et al., 2011). However, starvation completely overcomes ISC proliferation in Bursα-impaired midguts (Figure S6J). This is consistent with recent evidence showing that restrictive nutrient conditions, such as the absence of dietary methionine or its derivative S-adenosyl methionine, impair ISC proliferation in the adult fly midgut, even in the presence of activated mitogenic signaling pathways (Obata et al., 2018). Altogether, these data support a scenario in which starvation, while preventing systemic and local Bursα/DLgr2 signaling, would not result in induction of ISC proliferation as a side effect.

Implications of Our Work for Mammalian Systems

Drosophila DLgr2 is the ortholog of mammalian LGR4, -5, and -6 with closer homology to LGR4. While LGR5 and 6 are stem cell markers in several tissues, such as small intestine and skin, LGR4 depicts broader expression patterns and physiological functions (de Lau et al., 2011; Li et al., 2010; Wang et al., 2012b; Weng et al., 2008). LGR4, -5, and -6 are best known to enhance canonical Wnt signaling through binding to R-spondins (Carmon et al., 2011; de Lau et al., 2011). However, several lines of evidence support a more promiscuous binding affinity for LGR4, which can act as a canonical G-protein coupled receptor inducing iCa^{2+} and cyclic AMP signaling (Li et al., 2010; Wang et al., 2012a; Weng et al., 2008).

Interestingly, an activating variant of LGR4 (A750T) is linked to obesity in humans (Kettunen et al., 2009; Thorleifsson et al., 2009; Zou et al., 2017), while the non-sense mutation c.376C>T (p.R126X) is associated with reduced body weight (Styrkarsdottir et al., 2013). Recent reports show that LGR4 homozygous mutant ($LGR4^{m/m}$) mice display reduced adiposity and are resistant to diet- or leptin-induced obesity. These phenotypes appear to derive from increased energy expenditure through white-to-brown fat conversion (Wang et al., 2013) and are independent of Wnt signaling. The tissue and molecular mechanisms mediating this metabolic role of LGR4 remain unclear. Therefore, our paradigm may lead to a better understanding of LGR4's contribution to metabolic homeostasis and

disease. Importantly, our results highlight the intestine and ee cells in particular as central orchestrators of metabolic homeostasis and potential targets for the treatment of metabolic dysfunctions.

Limitations of Study

Bursicon is an insect-specific hormone. Therefore, direct mammalian translation of the signaling system presented here is unlikely. However, given the clear parallels between the metabolic functions of DLgr2 and LGR4, analysis of enteroendocrine cell-secreted factors in mammalian systems may reveal new and unexpected ligands for LGR4.

STAR★METHODS

Detailed methods are provided in the online version of this paper and include the following:

- KEY RESOURCES TABLE
- CONTACT FOR REAGENT AND RESOURCE SHARING
- EXPERIMENTAL MODEL AND SUBJECT DETAILS
  - Experimental Animals
  - Full Genotypes as They Appear in Each Figure Panel
- METHOD DETAILS
  - Starvation Sensitivity Assay
  - Immunofluorescence
  - Quantification of pH3^{+ve} Cells in the Posterior Midgut
  - Protein Extraction
  - Haemolymph Extraction
  - SDS-PAGE and Protein Transfer
  - Oxygen Consumption
  - Metabolomic Analysis by Liquid Chromatography-Mass Spectrometry (LC-MS)
  - RNA Extraction and qRT-PCR
  - Lipid Quantification
  - Glucose Quantification
  - Locomotor Assay
  - Chill Coma Recovery Assay
  - Glucose Absorption Assay
  - Food Intake Assay
  - Excretion Assay
- QUANTIFICATION AND STATISTICAL ANALYSIS

SUPPLEMENTAL INFORMATION

Supplemental Information includes six figures and one table and can be found with this article online at https://doi.org/10.1016/j.cmet.2018.09.021.

ACKNOWLEDGMENTS

We thank Irene Miguel-Aliaga, Benjamin White, John Ewer, Hans-Willi Honegger, Ronald Kühnlein, Jea H. Park, the Vienna *Drosophila* RNAi Center, and the Bloomington *Drosophila* Stock Center for providing *Drosophila* lines and reagents. We thank Owen Sansom, Ross L. Cagan, Eyal Gottlieb, and Saverio Tardito for insightful discussions throughout the course of this work. A.S., C.B., and Y.Y. are supported by Cancer Research UK core funding through the CRUK Beatson Institute (A17196). T.Z. and O.D.K.M are funded by CRUK Career Development Fellowship (to O.D.K.M.) C53309/A19702. B.K and D.J.M. are funded by ERC H2020 Marie Skłodowska Curie Actions Individual Fellowship 705190 "NuSiCC" and British Lung Foundation grant CSOBLF16-2. J.B.C. is a Sir Henry Dale Fellow jointly funded by the Wellcome Trust and the Royal Society (grant number 104103/Z/14/Z). In loving memory of Marcos Vidal.

AUTHOR CONTRIBUTIONS

A.S. and M.V. initiated the project. A.S. and C.B. designed and performed experiments and analyzed the data. Y.Y. provided technical support. T.Z. and O.D.K.M. performed and analyzed the $^{13}C_6$-D-glucose-tracing metabolomics experiments. B.K. and D.J.M. performed the mitochondrial O_2 consumption experiments. J.B.C. designed the experiments, analyzed the data, and directed the project. A.S., C.B., and J.B.C. wrote the manuscript.

DECLARATION OF INTERESTS

The authors declare no competing interests.

Received: December 19, 2017
Revised: August 10, 2018
Accepted: September 25, 2018
Published: October 18, 2018

REFERENCES

Baker, J.D., and Truman, J.W. (2002). Mutations in the *Drosophila* glycoprotein hormone receptor, rickets, eliminate neuropeptide-induced tanning and selectively block a stereotyped behavioral program. J. Exp. Biol. *205*, 2555–2565.

Balakireva, M., Gendre, N., Stocker, R.F., and Ferveur, J.F. (2000). The genetic variant Voila causes gustatory defects during *Drosophila* development. J. Neurosci. *20*, 3425–3433.

Beller, M., Bulankina, A.V., Hsiao, H.H., Urlaub, H., Jackle, H., and Kuhnlein, R.P. (2010). PERILIPIN-dependent control of lipid droplet structure and fat storage in *Drosophila*. Cell Metab. *12*, 521–532.

Bharucha, K.N., Tarr, P., and Zipursky, S.L. (2008). A glucagon-like endocrine pathway in *Drosophila* modulates both lipid and carbohydrate homeostasis. J. Exp. Biol. *211*, 3103–3110.

Bi, J., Wang, W., Liu, Z., Huang, X., Jiang, Q., Liu, G., Wang, Y., and Huang, X. (2014). Seipin promotes adipose tissue fat storage through the ER Ca(2)(+)-ATPase SERCA. Cell Metab. *19*, 861–871.

Birse, R.T., Soderberg, J.A., Luo, J., Winther, A.M., and Nassel, D.R. (2011). Regulation of insulin-producing cells in the adult *Drosophila* brain via the tachykinin peptide receptor DTKR. J. Exp. Biol. *214*, 4201–4208.

Britton, J.S., Lockwood, W.K., Li, L., Cohen, S.M., and Edgar, B.A. (2002). *Drosophila*'s insulin/PI3-kinase pathway coordinates cellular metabolism with nutritional conditions. Dev. Cell *2*, 239–249.

Brogiolo, W., Stocker, H., Ikeya, T., Rintelen, F., Fernandez, R., and Hafen, E. (2001). An evolutionarily conserved function of the *Drosophila* insulin receptor and insulin-like peptides in growth control. Curr. Biol. *11*, 213–221.

Buch, S., Melcher, C., Bauer, M., Katzenberger, J., and Pankratz, M.J. (2008). Opposing effects of dietary protein and sugar regulate a transcriptional target of *Drosophila* insulin-like peptide signaling. Cell Metab. *7*, 321–332.

Campbell, J.E., and Drucker, D.J. (2013). Pharmacology, physiology, and mechanisms of incretin hormone action. Cell Metab. *17*, 819–837.

Cani, P.D., Holst, J.J., Drucker, D.J., Delzenne, N.M., Thorens, B., Burcelin, R., and Knauf, C. (2007). GLUT2 and the incretin receptors are involved in glucose-induced incretin secretion. Mol. Cell. Endocrinol. *276*, 18–23.

Carmon, K.S., Gong, X., Lin, Q., Thomas, A., and Liu, Q. (2011). R-spondins function as ligands of the orphan receptors LGR4 and LGR5 to regulate Wnt/beta-catenin signaling. Proc. Natl. Acad. Sci. U S A *108*, 11452–11457.

Chen, J., Kim, S.M., and Kwon, J.Y. (2016). A systematic analysis of *Drosophila* regulatory peptide expression in enteroendocrine cells. Mol. Cells *39*, 358–366.

Choi, N.H., Lucchetta, E., and Ohlstein, B. (2011). Nonautonomous regulation of *Drosophila* midgut stem cell proliferation by the insulin-signaling pathway. Proc. Natl. Acad. Sci. U S A *108*, 18702–18707.

Colombani, J., Raisin, S., Pantalacci, S., Radimerski, T., Montagne, J., and Leopold, P. (2003). A nutrient sensor mechanism controls *Drosophila* growth. Cell *114*, 739–749.

de Lau, W., Barker, N., Low, T.Y., Koo, B.K., Li, V.S., Teunissen, H., Kujala, P., Haegebarth, A., Peters, P.J., van de Wetering, M., et al. (2011). Lgr5 homologues associate with Wnt receptors and mediate R-spondin signalling. Nature *476*, 293–297.

Dewey, E.M., McNabb, S.L., Ewer, J., Kuo, G.R., Takanishi, C.L., Truman, J.W., and Honegger, H.W. (2004). Identification of the gene encoding Bursicon, an insect neuropeptide responsible for cuticle sclerotization and wing spreading. Curr. Biol. *14*, 1208–1213.

Diao, F., and White, B.H. (2012). A novel approach for directing transgene expression in *Drosophila*: T2A-Gal4 in-frame fusion. Genetics *190*, 1139–1144.

Dutta, D., Dobson, A.J., Houtz, P.L., Glasser, C., Revah, J., Korzelius, J., Patel, P.H., Edgar, B.A., and Buchon, N. (2015). Regional cell-specific transcriptome mapping reveals regulatory complexity in the adult *Drosophila* midgut. Cell Rep. *12*, 346–358.

Engelstoft, M.S., Egerod, K.L., Holst, B., and Schwartz, T.W. (2008). A gut feeling for obesity: 7TM sensors on enteroendocrine cells. Cell Metab. *8*, 447–449.

Field, B.C., Chaudhri, O.B., and Bloom, S.R. (2010). Bowels control brain: gut hormones and obesity. Nat. Rev. Endocrinol. *6*, 444–453.

Galikova, M., Diesner, M., Klepsatel, P., Hehlert, P., Xu, Y., Bickmeyer, I., Predel, R., and Kuhnlein, R.P. (2015). Energy homeostasis control in *Drosophila* adipokinetic hormone mutants. Genetics *201*, 665–683.

Gautron, L., Elmquist, J.K., and Williams, K.W. (2015). Neural control of energy balance: translating circuits to therapies. Cell *161*, 133–145.

Geminard, C., Rulifson, E.J., and Leopold, P. (2009). Remote control of insulin secretion by fat cells in *Drosophila*. Cell Metab. *10*, 199–207.

Ghosh, A.C., and O'Connor, M.B. (2014). Systemic activin signaling independently regulates sugar homeostasis, cellular metabolism, and pH balance in *Drosophila melanogaster*. Proc. Natl. Acad. Sci. U S A *111*, 5729–5734.

Gribble, F.M., and Reimann, F. (2016). Enteroendocrine cells: chemosensors in the intestinal epithelium. Annu. Rev. Physiol. *78*, 277–299.

Gronke, S., Clarke, D.F., Broughton, S., Andrews, T.D., and Partridge, L. (2010). Molecular evolution and functional characterization of *Drosophila* insulin-like peptides. PLoS Genet. *6*, e1000857.

Gronke, S., Muller, G., Hirsch, J., Fellert, S., Andreou, A., Haase, T., Jackle, H., and Kuhnlein, R.P. (2007). Dual lipolytic control of body fat storage and mobilization in *Drosophila*. PLoS Biol. *5*, e137.

Gutierrez, E., Wiggins, D., Fielding, B., and Gould, A.P. (2007). Specialized hepatocyte-like cells regulate *Drosophila* lipid metabolism. Nature *445*, 275–280.

Haber, A.L., Biton, M., Rogel, N., Herbst, R.H., Shekhar, K., Smillie, C., Burgin, G., Delorey, T.M., Howitt, M.R., Katz, Y., et al. (2017). A single-cell survey of the small intestinal epithelium. Nature *551*, 333–339.

Hasygar, K., and Hietakangas, V. (2014). p53- and ERK7-dependent ribosome surveillance response regulates *Drosophila* insulin-like peptide secretion. PLoS Genet. *10*, e1004764.

Holst, L.S., Langin, D., Mulder, H., Laurell, H., Grober, J., Bergh, A., Mohrenweiser, H.W., Edgren, G., and Holm, C. (1996). Molecular cloning, genomic organization, and expression of a testicular isoform of hormone-sensitive lipase. Genomics *35*, 441–447.

Honegger, H.W., Estevez-Lao, T.Y., and Hillyer, J.F. (2011). Bursicon-expressing neurons undergo apoptosis after adult ecdysis in the mosquito *Anopheles gambiae*. J. Insect Physiol. *57*, 1017–1022.

Hulbert, A.J., Clancy, D.J., Mair, W., Braeckman, B.P., Gems, D., and Partridge, L. (2004). Metabolic rate is not reduced by dietary-restriction or by lowered insulin/IGF-1 signalling and is not correlated with individual lifespan in *Drosophila melanogaster*. Exp. Gerontol. *39*, 1137–1143.

Ikeya, T., Galic, M., Belawat, P., Nairz, K., and Hafen, E. (2002). Nutrient-dependent expression of insulin-like peptides from neuroendocrine cells in the CNS contributes to growth regulation in *Drosophila*. Curr. Biol. *12*, 1293–1300.

Jiang, H., Patel, P.H., Kohlmaier, A., Grenley, M.O., McEwen, D.G., and Edgar, B.A. (2009). Cytokine/Jak/Stat signaling mediates regeneration and homeostasis in the *Drosophila* midgut. Cell *137*, 1343–1355.

Junger, M.A., Rintelen, F., Stocker, H., Wasserman, J.D., Vegh, M., Radimerski, T., Greenberg, M.E., and Hafen, E. (2003). The *Drosophila* forkhead transcription factor FOXO mediates the reduction in cell number associated with reduced insulin signaling. J. Biol. *2*, 20.

Kettunen, J., Perola, M., Martin, N.G., Cornes, B.K., Wilson, S.G., Montgomery, G.W., Benyamin, B., Harris, J.R., Boomsma, D., Willemsen, G., et al.; GenomEUtwin-project (2009). Multicenter dizygotic twin cohort study confirms two linkage susceptibility loci for body mass index at 3q29 and 7q36 and identifies three further potential novel loci. Int. J. Obes. (Lond.) *33*, 1235–1242.

Kim, S.K., and Rulifson, E.J. (2004). Conserved mechanisms of glucose sensing and regulation by *Drosophila* corpora cardiaca cells. Nature *431*, 316–320.

Koyama, T., and Mirth, C.K. (2016). Correction: growth-blocking peptides as nutrition-sensitive signals for insulin secretion and body size regulation. PLoS Biol. *14*, e1002551.

Kuhnlein, R.P. (2011). The contribution of the *Drosophila* model to lipid droplet research. Prog. Lipid Res. *50*, 348–356.

Lahr, E.C., Dean, D., and Ewer, J. (2012). Genetic analysis of ecdysis behavior in *Drosophila* reveals partially overlapping functions of two unrelated neuropeptides. J. Neurosci. *32*, 6819–6829.

Lebrun, L.J., Lenaerts, K., Kiers, D., Pais de Barros, J.P., Le Guern, N., Plesnik, J., Thomas, C., Bourgeois, T., Dejong, C.H.C., Kox, M., et al. (2017). Enteroendocrine L cells sense LPS after gut barrier injury to enhance GLP-1 secretion. Cell Rep. *21*, 1160–1168.

Lee, G., and Park, J.H. (2004). Hemolymph sugar homeostasis and starvation-induced hyperactivity affected by genetic manipulations of the adipokinetic hormone-encoding gene in *Drosophila melanogaster*. Genetics *167*, 311–323.

Lemaitre, B., and Miguel-Aliaga, I. (2013). The digestive tract of *Drosophila melanogaster*. Annu. Rev. Genet. *47*, 377–404.

Li, X.Y., Lu, Y., Sun, H.Y., Wang, J.Q., Yang, J., Zhang, H.J., Fan, N.G., Xu, J., Jiang, J.J., Liu, R.Y., et al. (2010). G protein-coupled receptor 48 upregulates estrogen receptor alpha expression via cAMP/PKA signaling in the male reproductive tract. Development *137*, 151–157.

Luo, C.W., Dewey, E.M., Sudo, S., Ewer, J., Hsu, S.Y., Honegger, H.W., and Hsueh, A.J. (2005). Bursicon, the insect cuticle-hardening hormone, is a heterodimeric cystine knot protein that activates G protein-coupled receptor LGR2. Proc. Natl. Acad. Sci. U S A *102*, 2820–2825.

Ma, Z., Liu, Z., and Huang, X. (2012). Membrane phospholipid asymmetry counters the adverse effects of sterol overloading in the Golgi membrane of *Drosophila*. Genetics *190*, 1299–1308.

Maddocks, O.D.K., Athineos, D., Cheung, E.C., Lee, P., Zhang, T., van den Broek, N.J.F., Mackay, G.M., Labuschagne, C.F., Gay, D., Kruiswijk, F., et al. (2017). Modulating the therapeutic response of tumours to dietary serine and glycine starvation. Nature *544*, 372–376.

Masuyama, K., Zhang, Y., Rao, Y., and Wang, J.W. (2012). Mapping neural circuits with activity-dependent nuclear import of a transcription factor. J. Neurogenet. *26*, 89–102.

Mattila, J., and Hietakangas, V. (2017). Regulation of carbohydrate energy metabolism in *Drosophila melanogaster*. Genetics *207*, 1231–1253.

Mayer, E.A. (2011). Gut feelings: the emerging biology of gut-brain communication. Nat. Rev. Neurosci. *12*, 453–466.

Mendive, F.M., Van Loy, T., Claeysen, S., Poels, J., Williamson, M., Hauser, F., Grimmelikhuijzen, C.J., Vassart, G., and Vanden Broeck, J. (2005). *Drosophila* molting neurohormone Bursicon is a heterodimer and the natural agonist of the orphan receptor DLGR2. FEBS Lett. *579*, 2171–2176.

Miguel-Aliaga, I. (2012). Nerveless and gutsy: intestinal nutrient sensing from invertebrates to humans. Semin. Cell Dev. Biol. *23*, 614–620.

Moran-Ramos, S., Tovar, A.R., and Torres, N. (2012). Diet: friend or foe of enteroendocrine cells–how it interacts with enteroendocrine cells. Adv. Nutr. *3*, 8–20.

Moraru, A., Cakan-Akdogan, G., Strassburger, K., Males, M., Mueller, S., Jabs, M., Muelleder, M., Frejno, M., Braeckman, B.P., Ralser, M., and Teleman, A.A. (2017). THADA regulates the organismal balance between energy storage and heat production. Dev. Cell *41*, 450.

Musselman, L.P., Fink, J.L., Ramachandran, P.V., Patterson, B.W., Okunade, A.L., Maier, E., Brent, M.R., Turk, J., and Baranski, T.J. (2013). Role of fat body lipogenesis in protection against the effects of caloric overload in *Drosophila*. J. Biol. Chem. *288*, 8028–8042.

O'Brien, L.E., Soliman, S.S., Li, X., and Bilder, D. (2011). Altered modes of stem cell division drive adaptive intestinal growth. Cell *147*, 603–614.

Obata, F., Tsuda-Sakurai, K., Yamazaki, T., Nishio, R., Nishimura, K., Kimura, M., Funakoshi, M., and Miura, M. (2018). Nutritional control of stem cell division through S-adenosylmethionine in *Drosophila* intestine. Dev. Cell *44*, 741–751.e3.

Park, J.H., Chen, J., Jang, S., Ahn, T.J., Kang, K., Choi, M.S., and Kwon, J.Y. (2016). A subset of enteroendocrine cells is activated by amino acids in the *Drosophila* midgut. FEBS Lett. *590*, 493–500.

Park, S., Alfa, R.W., Topper, S.M., Kim, G.E., Kockel, L., and Kim, S.K. (2014). A genetic strategy to measure circulating *Drosophila* insulin reveals genes regulating insulin production and secretion. PLoS Genet. *10*, e1004555.

Peabody, N.C., Diao, F., Luan, H., Wang, H., Dewey, E.M., Honegger, H.W., and White, B.H. (2008). Bursicon functions within the *Drosophila* CNS to modulate wing expansion behavior, hormone secretion, and cell death. J. Neurosci. *28*, 14379–14391.

Puig, O., and Tjian, R. (2005). Transcriptional feedback control of insulin receptor by dFOXO/FOXO1. Genes Dev. *19*, 2435–2446.

Rulifson, E.J., Kim, S.K., and Nusse, R. (2002). Ablation of insulin-producing neurons in flies: growth and diabetic phenotypes. Science *296*, 1118–1120.

Saltiel, A.R., and Kahn, C.R. (2001). Insulin signalling and the regulation of glucose and lipid metabolism. Nature *414*, 799–806.

Scopelliti, A., Bauer, C., Cordero, J.B., and Vidal, M. (2016). Bursicon-alpha subunit modulates dLGR2 activity in the adult *Drosophila melanogaster* midgut independently to Bursicon-beta. Cell Cycle *15*, 1538–1544.

Scopelliti, A., Cordero, J.B., Diao, F., Strathdee, K., White, B.H., Sansom, O.J., and Vidal, M. (2014). Local control of intestinal stem cell homeostasis by enteroendocrine cells in the adult *Drosophila* midgut. Curr. Biol. *24*, 1199–1211.

Shim, J., Gururaja-Rao, S., and Banerjee, U. (2013). Nutritional regulation of stem and progenitor cells in *Drosophila*. Development *140*, 4647–4656.

Song, W., Cheng, D., Hong, S., Sappe, B., Hu, Y., Wei, N., Zhu, C., O'Connor, M.B., Pissios, P., and Perrimon, N. (2017). Midgut-derived activin regulates glucagon-like action in the fat body and glycemic control. Cell Metab. *25*, 386–399.

Song, W., Veenstra, J.A., and Perrimon, N. (2014). Control of lipid metabolism by tachykinin in *Drosophila*. Cell Rep. *9*, 40–47.

Steinert, R.E., and Beglinger, C. (2011). Nutrient sensing in the gut: interactions between chemosensory cells, visceral afferents and the secretion of satiation peptides. Physiol. Behav. *105*, 62–70.

Styrkarsdottir, U., Thorleifsson, G., Sulem, P., Gudbjartsson, D.F., Sigurdsson, A., Jonasdottir, A., Jonasdottir, A., Oddsson, A., Helgason, A., Magnusson, O.T., et al. (2013). Nonsense mutation in the LGR4 gene is associated with several human diseases and other traits. Nature *497*, 517–520.

Talay, M., Richman, E.B., Snell, N.J., Hartmann, G.G., Fisher, J.D., Sorkac, A., Santoyo, J.F., Chou-Freed, C., Nair, N., Johnson, M., et al. (2017). Transsynaptic mapping of second-order taste neurons in flies by trans-Tango. Neuron *96*, 783–795.e4.

Thorleifsson, G., Walters, G.B., Gudbjartsson, D.F., Steinthorsdottir, V., Sulem, P., Helgadottir, A., Styrkarsdottir, U., Gretarsdottir, S., Thorlacius, S., Jonsdottir, I., et al. (2009). Genome-wide association yields new sequence variants at seven loci that associate with measures of obesity. Nat. Genet. *41*, 18–24.

Wang, B., Komers, R., Carew, R., Winbanks, C.E., Xu, B., Herman-Edelstein, M., Koh, P., Thomas, M., Jandeleit-Dahm, K., Gregorevic, P., et al. (2012a). Suppression of microRNA-29 expression by TGF-beta1 promotes collagen expression and renal fibrosis. J. Am. Soc. Nephrol. *23*, 252–265.

Wang, J., Li, X., Ke, Y., Lu, Y., Wang, F., Fan, N., Sun, H., Zhang, H., Liu, R., Yang, J., et al. (2012b). GPR48 increases mineralocorticoid receptor gene expression. J. Am. Soc. Nephrol. *23*, 281–293.

Wang, J., Liu, R., Wang, F., Hong, J., Li, X., Chen, M., Ke, Y., Zhang, X., Ma, Q., Wang, R., et al. (2013). Ablation of LGR4 promotes energy expenditure by driving white-to-brown fat switch. Nat. Cell Biol. *15*, 1455–1463.

Wang, L., Karpac, J., and Jasper, H. (2014). Promoting longevity by maintaining metabolic and proliferative homeostasis. J. Exp. Biol. *217*, 109–118.

Weng, J., Luo, J., Cheng, X., Jin, C., Zhou, X., Qu, J., Tu, L., Ai, D., Li, D., Wang, J., et al. (2008). Deletion of G protein-coupled receptor 48 leads to ocular anterior segment dysgenesis (ASD) through down-regulation of Pitx2. Proc. Natl. Acad. Sci. U S A *105*, 6081–6086.

Worthington, J.J., Reimann, F., and Gribble, F.M. (2017). Enteroendocrine cells-sensory sentinels of the intestinal environment and orchestrators of mucosal immunity. Mucosal Immunol. *11*, 3–20.

Yan, K.S., Gevaert, O., Zheng, G.X.Y., Anchang, B., Probert, C.S., Larkin, K.A., Davies, P.S., Cheng, Z.F., Kaddis, J.S., Han, A., et al. (2017). Intestinal enteroendocrine lineage cells possess homeostatic and injury-inducible stem cell activity. Cell Stem Cell *21*, 78–90.e6.

Zhao, X., and Karpac, J. (2017). Muscle directs diurnal energy homeostasis through a myokine-dependent hormone module in *Drosophila*. Curr. Biol. *27*, 1941–1955.e6.

Zietek, T., and Daniel, H. (2015). Intestinal nutrient sensing and blood glucose control. Curr. Opin. Clin. Nutr. Metab. Care *18*, 381–388.

Zinke, I., Kirchner, C., Chao, L.C., Tetzlaff, M.T., and Pankratz, M.J. (1999). Suppression of food intake and growth by amino acids in *Drosophila*: the role of pumpless, a fat body expressed gene with homology to vertebrate glycine cleavage system. Development *126*, 5275–5284.

Zou, Y., Ning, T., Shi, J., Chen, M., Ding, L., Huang, Y., Kauderer, S., Xu, M., Cui, B., Bi, Y., et al. (2017). Association of a gain-of-function variant in LGR4 with central obesity. Obesity *25*, 252–260.

STAR★METHODS

KEY RESOURCES TABLE

REAGENT or RESOURCE	SOURCE	IDENTIFIER
Antibodies		
Anti-GFP (chicken)	Abcam	Cat# ab13970, RRID: AB_300798
Anti-Pros (mouse)	DSHB	Cat# Prospero (MR1A), RRID: AB_528440
Anti-Burs (rabbit)	(Peabody et al., 2008)	N/A
Anti-Burs (rabbit)	(Scopelliti et al., 2016)	N/A
Anti-AKH (rabbit)	(Lee and Park, 2004)	N/A
Anti-αTub (mouse)	DSHB	Cat# E7, RRID: AB_528499 (concentrate)
Anti-pH3S10 (rabbit)	Cell Signaling Technology	Cat# 9701, RRID: AB_331535
Anti-pH3S28 (rabbit)	Cell Signaling Technology	Cat# 9713S, RRID: AB_823532
Anti-Brp	DSHB	Cat# nc82, RRID: AB_2314866
anti-chicken-IgG-488	Invitrogen	Cat# A-11039, RRID: AB_142924
anti-mouse-IgG-488	Invitrogen	Cat# A-11029, RRID: AB_138404
anti-mouse-IgG-546	Invitrogen	Cat# A-11030, RRID: AB_2534089
anti-mouse-IgG-594	Invitrogen	Cat# A-11032, RRID: AB_141672
anti-rabbit-IgG-488	Invitrogen	Cat# A-11008, RRID: AB_143165
anti-rabbit-IgG-546	Thermo Fisher Scientific	Cat# A-11035, RRID: AB_2534093
anti-rabbit-IgG-594	Thermo Fisher Scientific	Cat# A-11037, RRID: AB_2534095
anti-rabbit-IgG-647	Thermo Fisher Scientific	Cat# A-21245; RRID: AB_2535813
IRDye 680RD- anti rabbit	LI-COR Biosciences	Cat# 926-68073, RRID: AB_10954442
IRDye 800RD- anti mouse	LI-COR Biosciences	Cat# 926-32212, RRID: AB_621847
Chemicals, Peptides, and Recombinant Proteins		
High Capacity cDNA Reverse Transcription Kit	Applied Biosystems	Cat# 4368813
PerfeCTa SYBR Green FastMix (Low ROX)	Quanta bio	Cat# 95074-012
LipidTOX	life technologies	Cat# H34476
2-NBDG	Invitrogen	Cat# N13195
Lipase for Triglyceride Quantification Kit	Abcam	Cat# ab89001
NuPAGE 10% Bis-Tris Protein Gels, 1.5 mm, 10-well	Invitrogen NuPAGE	Cat# NO0315BOX
Amersham Protran 0.1 NC nitrocellulose	GE Healthcare Life Sciences	Cat# GE10600000
Vectashield mounting media containing DAPI	Vector Laboratories	Cat# H-1200, RRID: AB_2336790
SeQuant ZIC-pHILIC column (4.6mm x 150mm, 5μm)	Merck	Cat# 150461
Excel SuperC18 column (3.0mm x 150mm, 3μm)	ACE Excel	Cat# EXL-1111-1503U
Critical Commercial Assays		
Free Fatty Acid Assay Kit - Quantification	Abcam	Cat# ab65341
Glucose Colorimetric Assay Kit	Cayman Chemical	Cat# 10010098
RNAeasy Mini Kit (50)	Qiagen	Cat# 74104
Mlitochondria Isolation Kit for Tissues	Sigma Aldrich	Cat# MITOISO1-1KT
Experimental Models: Organisms/Strains		
w^{1118}; +; +	N/A	N/A
+; +; $burs^{z5569}$	(Dewey et al., 2004)	N/A
w^{1118}; cn, bw, rk^{1}; +	Bloomington	Cat# 3589, RRID: BDSC_3589
Df(2) 110	(Lahr et al., 2012)	N/A
Df(2) Excel6035	Bloomington	Cat# 7518, RRID: BDSC_7518
Akh^{A}	(Galikova et al., 2015)	N/A
Akh^{SAP}	(Galikova et al., 2015)	N/A
UAS-*lgr2* IR	VDRC	Cat# FBst0458239, RRID: FlyBase_FBst0458239; 29931

(Continued on next page)

Continued

REAGENT or RESOURCE	SOURCE	IDENTIFIER
UAS-*bursα* IR	VDRC	Cat# FBst0479587, RRID: FlyBase_FBst0479587; 102204
UAS-*bursα* IR	VDRC	Cat# FBst0451049, RRID: FlyBase_FBst0451049; 13520
UAS-*glut1* IR	VDRC	Cat# FBst0450948, RRID: FlyBase_FBst0450948; 13326
UAS-*akh* IR	VDRC	Cat# FBst0450258, RRID: FlyBase_FBst0450258; 11352
UAS-*akh* IR	VDRC	Cat# FBst0475991, RRID: FlyBase_FBst0475991; 105063
UAS-*akhr* IR	VDRC	Cat# FBst0479003, RRID: FlyBase_FBst0479003; 109300
UAS-*plc21c* IR	VDRC	108395 kk
UAS-*ip3r* IR	Bloomington	Cat# 51686, RRID: BDSC_51686
UAS-*dhsl* IR	VDRC	Cat# FBst0473619, RRID: FlyBase_FBst0473619; 109336
how-Gal4	(Jiang et al., 2009)	N/A
Voila-Gal4	(Balakireva et al., 2000)	N/A
nSyb-Gal4	Irene Miguel-Aliaga	N/A
elav-Gal4	Bloomington	Cat# 8760, RRID: BDSC_8760
lgr2^{PAN}-Gal4	(Diao and White, 2012)	N/A
lgr2^{Tgem}-Gal4	Benjamin White	N/A
FB-Gal4	(Ghosh and O'Connor, 2014)	N/A
ppl-Gal4	(Zinke et al., 1999)	N/A
Lsp2-Gal4	Bloomington	Cat# 6357, RRID: BDSC_6357
DSRF-Gal4	Irene Miguel-Aliaga	N/A
AKH-Gal4	Bloomington	Cat# 25683, RRID: BDSC_25683
UAS-*mCD8.mRFP*	Bloomington	Cat# 27339; RRID: BDSC_27399
20XUAS-*shi*^{ts}*[DN]*	Bloomington	Cat# 66599; RRID: BDSC_66599
tubP-gal80^{ts}	Bloomington	Cat# 7018; RRID: BDSC_7019
tubP-gal80^{ts}	Bloomington	Cat# 7019, RRID: BDSC_7019
UAS-Dicer2	Bloomington	Cat# 24646, RRID: BDSC_24646
UAS-*burs77*	(Scopelliti et al., 2014)	N/A
UAS-*lgr2*^{KS}	(Scopelliti et al., 2014)	N/A
UAS-2xEGFP	Bloomington	Cat# 6874, RRID: BDSC_6874
UAS-Myr-GFP	M. Texada	N/A
UAS-CD8-GFP	Irene Miguel-Aliaga	N/A
UAS-nRS	Benjamin White	N/A
LexAop-CD8GFP; UAS-mLexA-VP16-NFAT, exAop-rCD2-GFP (CaLexA)	Bloomington	Cat# 66542, RRID: BDSC_66542
tGPH	Bloomington	Cat# 8163, RRID: BDSC_8163
UAS-myrGFP.QUAS-mtdTomato-3xHA; trans-Tango (Trans-Tango)	Bloomington	Cat# 77124; RRID: BDSC_77124
**Oligonucleotides**		
Primers for mRNA expression	This paper	See Table S1
**Software and Algorithms**		
GraphPad Prism7	GraphPad Software	RRID: SCR_002798
Fiji	Fiji	RRID: SCR_002285
Zen 2 lite	Zeiss	ZEN Digital Imaging for Light Microscopy, RRID: SCR_013672
QuickTime Pro	QuickTime	N/A

(*Continued on next page*)

Continued

REAGENT or RESOURCE	SOURCE	IDENTIFIER
7500 Real-Time PCR Software	Applied Biosystems	RRID: SCR_014596
OxygraphPlus software	Hansatech Instruments	N/A
ProteoWizard	http://proteowizard.sourceforge.net/	N/A
MZMine 2.10	http://mzmine.github.io/	N/A
Other		
LSM710 microscope	Zeiss	N/A
Zeiss LSM 800 with Airyscan	Zeiss	RRID: SCR_015963
ABI7500	Applied Biosystems	N/A
Safire^2 plate reader	TECAN	N/A
Sunrise plate reader	TECAN	N/A
Clark-type oxygen-sensitive electrode	Hansatech Instruments	http://www.hansatech-instruments.com/products/introduction-to-oxygen-measurements/complete-systems/2079-2/
XCell *SureLock* electrophoresis system	Invitrogen	N/A
ODYSSEY CLx	LI-COR	RRID: SCR_014579

CONTACT FOR REAGENT AND RESOURCE SHARING

Requests for further information, reagents and resources should be directed to and will be fulfilled by the Lead Contact, Julia B. Cordero (julia.cordero@glasgow.ac.uk).

EXPERIMENTAL MODEL AND SUBJECT DETAILS

Experimental Animals

Species Used

Drosophila melanogaster.

Animal Breeding and Maintenance

Flies were mated and kept on standard food in humidity-controlled incubators in a 12h light- 12h dark cycle. Experiments involving mutants were carried out at 25°C. Crosses for adult specific targeted knockdown were kept at 18°C. F1s were allowed to eclose for 2-3 days. Animals of the desired genotype were then collected and transferred to 29°C for transgene activation. Unless otherwise stated, animals were fed a standard diet containing 10g Agar, 15g Sucrose, 30g Glucose, 15g Maize meal, 10g wheat germ, 30g treacle and 10g Soya flour per litre of distilled water. All experiments were performed or started at Zeitgeber 22-24.

Sex

Only mated females were used throughout this study.

Full Genotypes as They Appear in Each Figure Panel

Figure 1

A, B	*w^{1118}; +; +*
C, D	*UAS-Dicer2/+; tub-gal80^{ts}/+; Voila-Gal4/+*
	UAS-Dicer2/+; tub-gal80^{ts}/UAS-glut1 IR; Voila-Gal4/+
E	*+; tub-gal80ts/UAS-burs77; Voila-Gal4/+*
F	*w^{1118}; +; +*
	+; +; burs^{z5569}
	w^{1118}; cn, bw, rk^{1}; +
	+; +; bursβ $^{Df(2)\ 110/\ Df(2)\ Excel6035}$
G	*UAS-Dicer2/+; tub-gal80^{ts}/+; Voila-Gal4/+*
	UAS-Dicer2/+; tub-gal80^{ts}/ UAS-bursα IR^{KK}; Voila-Gal4/UAS-bursα IR^{GD}
	UAS-Dicer2/+; tub-gal80^{ts}/UAS-glut1 IR; Voila-Gal4/+

Figure 2

A, B	*w^{1118}; +; +*
	+; +; $burs^{z5569}$
	w^{1118}; cn, bw, rk^{1}; +
	+; +; $burs\beta^{Df(2)\ 110/\ Df(2)\ Excel6035}$
C, D	*UAS-Dicer2/+; tub-$gal80^{ts}$/+; Voila-Gal4/+*
	UAS-Dicer2/+; tub-$gal80^{ts}$/ UAS-bursα IR^{KK}; Voila-Gal4/UAS-bursα IR^{GD}
	UAS-Dicer2/+; tub-$gal80^{ts}$/UAS-glut1 IR; Voila-Gal4/+
E, F, H, J	*UAS-Dicer2/+; tub-$gal80^{ts}$/+; Voila-Gal4/+*
	UAS-Dicer2/+; tub-$gal80^{ts}$/ UAS-bursα IR^{KK}; Voila-Gal4/UAS-bursα IR^{GD}
G	*w^{1118}; +; +*
	+; +; $burs^{z5569}$
I	*w^{1118}; +; +*
	+; +; $burs^{z5569}$
	w^{1118}; cn, bw, rk^{1}; +

Figure 3

A	*w^{1118}; +; +*
B	*w^{1118}; +; +*
	UAS-Dicer2/+; tub-$gal80^{ts}$/+; Voila-Gal4/+
	UAS-Dicer2/+; tub-$gal80^{ts}$/ UAS-bursα IR^{KK}; Voila-Gal4/UAS-bursα IR^{GD}
C	*+; UAS-CD8-GFP/+; $dlgr2^{PAN}$-Gal4/+*
D, E	*+; +; $dlgr2^{PAN}$-Gal4/+*
	+; +; UAS-shi^{ts}[DN]/+
	+; +; $dlgr2^{PAN}$-Gal4/UAS-shi^{ts}[DN]
F-K	*UAS-Dicer2/+; nSyb-Gal4/+; tub-$gal80^{ts}$/+*
	UAS-Dicer2/+; nSyb-Gal4/+; tub-$gal80^{ts}$/UAS- dlgr2 IR^{GD}

Figure 4

A, B	*+; nSyb-Gal4/+; Voila-Gal4/+*
	+; UAS-bursα IR^{KK}/+; UAS-dlgr2 IR^{GD}/+
	+; nSyb-Gal4/ UAS-bursα IR^{KK}; Voila-Gal4/+
	+; nSyb-Gal4/+; Voila-Gal4/ UAS-dlgr2 IR^{GD}
	+; nSyb-Gal4/ UAS-bursα IR^{KK}; Voila-Gal4/ UAS-dlgr2 IR^{GD}
C	w^{1118}; +; +
	w^{1118}; cn, bw, rk^{1}; +
	+; rk^{1}; UAS-$dlgr2^{KS}$/ +
	+; rk^{1}; elav-Gal4/ +
	+; rk^{1}; elav-Gal4/ UAS-$dlgr2^{KS}$
D	*w^{1118}; cn, bw, rk^{1}; +*
	+; rk^{1}; elav-Gal4/ UAS-$dlgr2^{KS}$
E	*+; rk^{1}; UAS-$dlgr2^{KS}$/ +*
	+; rk^{1}; elav-Gal4/ +
	+; rk^{1}; elav-Gal4/ UAS-$dlgr2^{KS}$

Figure 5

A	*UAS-Dicer2/+; tub-gal80^{ts}/+; Voila-Gal4/+*
	UAS-Dicer2/+; tub-gal80^{ts}/ UAS-bursα IR^{KK}; Voila-Gal4/UAS-bursα IR^{GD}
	UAS-Dicer2/+; nSyb-Gal4/+; tub-gal80^{ts}/+
	UAS-Dicer2/+; nSyb-Gal4/+; tub-gal80^{ts}/UAS- dlgr2 IR^{GD}
B	*+; dlgr2^{TGEM}-Gal4/+; UAS-Myr-GFP/+*
C	*QUAS-mtdTomato-3xHA, UAS-myr-GFP/+; dlgr2^{TGEM}-Gal4/ trans-Tango; +*
	QUAS-mtdTomato-3xHA, UAS-myr-GFP /+; +/trans-Tango; +
D	*+; ppl-Gal4/+; UAS-mLexA-VP16-NFAT, LexAop-rCD2-GFP/ UAS- dlgr2 IR^{GD}*
	+; ppl-Gal4/nSyb-Gal4; UAS-mLexA-VP16-NFAT, LexAop-rCD2GFP/ UAS- dlgr2 IR^{GD}
E	*w^{1118}; +; +*
	+; +; AKH$^{[A]}$/+
	+; rk^{1}; AKH$^{[A]}$/+
	+; +; AKH$^{[SAP]}$/+
	+; rk^{1}; AKH$^{[SAP]}$/+
	w1118; cn, bw, rk1; +
F, G	*UAS-Dicer2/+; nSyb-Gal4/+; tub-gal80^{ts}/+*
	UAS-Dicer2/+; nSyb-Gal4/+; tub-gal80^{ts}/UAS- dlgr2 IR^{GD}
	UAS-Dicer2/UAS-akh IR^{GD}; nSyb-Gal4/+; tub-gal80^{ts}/ +
	UAS-Dicer2/UAS-akh IR^{GD}; nSyb-Gal4/+; tub-gal80^{ts}/UAS- dlgr2 IR^{GD}
H	*UAS-Dicer2/+; nSyb-Gal4/+; tub-gal80^{ts}/UAS- dlgr2 IR^{GD}*
	UAS-Dicer2/UAS-akh IR^{GD}; nSyb-Gal4/+; tub-gal80^{ts}/UAS- dlgr2 IR^{GD}
I, J	*+; nSyb-Gal4/+; +*
	+; nSyb-Gal4/+; burs^{z5569}
	+; nSyb-Gal4/ UAS-akh IR KK; burs^{z5569}
K	*+; FB-Gal4/+; +*
	+; FB-Gal4/+; burs^{z5569}
	+; FB-Gal4/UAS-akhr IR ^{kk}; burs^{z5569}
	+; FB-Gal4/UAS-plc21c IR; burs^{z5569}
	+; FB-Gal4/UAS-IP3R IR; burs^{z5569}
L	*+; FB-Gal4/+; +*
	+; FB-Gal4/+; burs^{z5569}
	+; FB-Gal4/UAS-akhr IR ^{kk}; burs^{z5569}
	+; FB-Gal4/UAS-plc21c IR; burs^{z5569}
	+; FB-Gal4/UAS-IP3R IR; burs^{z5569}
	+; FB-Gal4/UAS-dhsl IR; burs^{z5569}

Figure S1

A, B	*w^{1118}; +; +*
C	*UAS-Dicer2/+; tub-gal80^{ts}/+; Voila-Gal4/+*
	UAS-Dicer2/+; tub-gal80^{ts}/UAS-glut1 IR; Voila-Gal4/+
D	*+; tub-gal80ts/UAS-burs77; Voila-Gal4/+*
E	*w^{1118}; +; +*
	w^{1118}/+; UAS-bursα IR^{KK}/+; UAS-bursα IR^{GD}/+
	w^{1118}/+; UAS-glut1 IR/+; +

CellPress

Figure S2

A, C	*w1118; +; +*
	+; +; burs^{z5569}
B, D-G, I-K	*UAS-Dicer2/+; tub-gal80^{ts}/+; Voila-Gal4/+*
	UAS-Dicer2/+; tub-gal80^{ts}/ UAS-bursα IR^{KK}; Voila-Gal4/UAS-bursα IR^{GD}
H, L	*w1118; +; +*
	+; +; burs^{z5569}
	w^{1118}; cn, bw, rk^{1}; +

Figure S3

A	*+; UAS-CD8-GFP/+; dlgr2^{PAN}-Gal4/+*
B-D	*UAS-Dicer2/+; tub-gal80^{ts} /+; how-Gal4/+*
	UAS-Dicer2/+; tub-gal80^{ts} /+; how-Gal4/UAS- dlgr2 IR^{GD}
E	*w1118; +; +*
	w^{1118}; cn, bw, rk^{1}; +
	+; rk^{1}; UAS-dlgr2^{KS}/ +
	+; rk^{1}; how-Gal4/ +
	+; rk^{1}; how-Gal4/ UAS-dlgr2^{KS}
F	*w1118; +; +*
G	*UAS-Dicer2/+; tub-gal80^{ts}/+; Voila-Gal4/+*
	UAS-Dicer2/+; tub-gal80^{ts}/ UAS-bursα IR^{KK}; Voila-Gal4/UAS-bursα IR^{GD}
H, I	*+; tub-gal80ts/+; Voila-Gal4/+*
	+; tub-gal80ts/+; Voila-Gal4/UAS-osbp
J	*+; UAS-nuclear Red stinger/+; dlgr2^{PAN}-Gal4/+*
K, L	*UAS-Dicer2/+; tub-gal80^{ts}/ +; Lsp2-Gal4/+*
	UAS-Dicer2/+; tub-gal80^{ts}/ +; Lsp2-Gal4/UAS-dlgr2 IR^{GD}
M, N	*UAS-Dicer2/+; tub-gal80^{ts}/ +; dsrf-Gal4/+*
	UAS-Dicer2/+; tub-gal80^{ts}/ +; dsrf-Gal4/UAS-dlgr2 IR^{GD}
O	*w1118; +; +*
	w^{1118}; cn, bw, rk^{1}; +
	+; +; UAS-dlgr2^{KS}/ +
	+; rk^{1}; UAS-dlgr2^{KS}/ +
	+; rk^{1}; Lsp2-Gal4/ +
	+; rk^{1}; Lsp2-Gal4/ UAS-dlgr2^{KS}
	+; rk^{1}; dsrf-Gal4/ +
	+; rk^{1}; dsrf-Gal4/ UAS-dlgr2^{KS}

Figure S4

A-D	*UAS-Dicer2/+; nSyb-Gal4/+; tub-gal80^{ts}/+*
	UAS-Dicer2/+; nSyb-Gal4/+; tub-gal80^{ts}/UAS- dlgr2 IR^{GD}
E, F	*UAS-Dicer2/+; nSyb-Gal4/+; tub-gal80^{ts}/+*
	UAS-Dicer2/+; nSyb-Gal4/+; tub-gal80^{ts}/UAS- dlgr2 IR^{GD}
	UAS-Dicer2/UAS-akh IR^{GD}; nSyb-Gal4/+; tub-gal80^{ts}/ +
	UAS-Dicer2/UAS-akh IR^{GD}; nSyb-Gal4/+; tub-gal80^{ts}/UAS- dlgr2 IR^{GD}

Figure S5

A, B	*UAS-Dicer2/+; tub-gal80^{ts}/+; Voila-Gal4/+*
	UAS-Dicer2/+; tub-gal80^{ts}/ UAS-bursα IR^{KK}; Voila-Gal4/UAS-bursα IR^{GD}
	UAS-Dicer2/+; nSyb-Gal4/+; tub-gal80^{ts}/+
	UAS-Dicer2/+; nSyb-Gal4/+; tub-gal80^{ts}/UAS- dlgr2 IR^{GD}
C	*UAS-Dicer2/+; nSyb-Gal4*/tGPH; *tub-gal80^{ts}*/+
	UAS-Dicer2/+; nSyb-Gal4/tGPH; tub-gal80^{ts}/UAS- dlgr2 IR^{GD}

Figure S6

A	*+; dlgr2^{TGEM}-Gal4/+; UAS-Myr-GFP/+*
B, C	*UAS-Dicer2/+; akh-Gal4/+; tub-gal80^{ts}/ +*
	UAS-Dicer2/+; akh-Gal4/+; tub-gal80^{ts}/UAS- dlgr2 IR^{GD}
D	*+; nSyb-Gal4/+; CD8-RFP/+*
E	*UAS-Dicer2/+; nSyb-Gal4/+; tub-gal80^{ts}/+*
	UAS-Dicer2/UAS-akh IR^{GD}; nSyb-Gal4/+; tub-gal80^{ts}/ +
F	*w^{1118}; +; +*
	w^{1118}/+; +; UAS- dlgr2 IR^{GD}/+
	w^{1118}/UAS-akh IR^{GD}; +; +
	w^{1118}/UAS-akh IR^{GD}; +; UAS- dlgr2 IR^{GD}/+
G	+*; FB-Gal4/+;* +
	+; FB-Gal4/UAS-akhr IR ^{kk}; +
	+; FB-Gal4/UAS-plc21c IR; +
	+; FB-Gal4/UAS-IP3R IR; +
	+; FB-Gal4/UAS-dhsl IR; +
H	*w^{1118}; +; +*
	w^{1118}/+; UAS-akhr IR ^{kk}/+; +
	w^{1118}/+; UAS-plc21c IR/+; +
	w^{1118}/+; UAS-IP3R IR/+; +
	w^{1118}/+; UAS-dhsl IR/+; +
I	*w^{1118}; +; +*
	+; +; burs^{z5569}
	+; UAS-akhr IR ^{kk}/+; burs^{z5569}
	+; UAS-plc21c IR/+; burs^{z5569}
	+; UAS-IP3R IR/+; burs^{z5569}
	+; UAS-dhsl IR/+; burs^{z556}
J	*UAS-Dicer2/+; tub-gal80^{ts}/+; Voila-Gal4/+*
	UAS-Dicer2/+; tub-gal80^{ts}/ UAS-bursα IR^{KK}; Voila-Gal4/UAS-bursα IR^{GD}
	UAS-Dicer2/+; tub-gal80^{ts}/UAS-glut1 IR; Voila-Gal4/+
	+; tub-gal80ts/+; Voila-Gal4/+
	+; tub-gal80ts/+; Voila-Gal4/UAS-osbp

METHOD DETAILS

Starvation Sensitivity Assay

Adult flies of the desired genotype were collected and aged for 7 days at 25°C, or for 10 days at 29°C and transferred into 1% agar (in dH_2O) containing vials. Dead animals were counted multiple times a day. Number of animals used are indicated in the figures. Log-rank (Mantel-Cox) test was used to assess statistical significance using Graph Pad Prism 7.

Immunofluorescence

Tissues were dissected and fixed in 4% para-formaldehyde (Polysciences) at room temperature for at least 30 min. After fixation, tissues were transferred first to fresh PBS for 5 min, then into PBS + 0.2% TritonX-100 (PBST) for 20 min, followed by overnight incubation at 4°C with primary antibodies in PBST + 2% Bovine Serum Albumin (BSA). Samples were then washed in PBST for 1h and incubated with secondary antibodies in PBST for 2h at room temperature, washed in PBST for 1h and mounted onto polylysine glass slides (Thermo Fisher) with 13mm x 0.12mm spacers (Electron Microscopy Science) and Vectashield mounting media containing DAPI (Vector Laboratories, Inc).

Midguts stained for Bursα were dehydrated in a series of ethanol washes ranging from 10% to 90% on ice after fixation in 4% para-formaldehyde. Samples were kept in 90% ethanol over night at -20°C followed by serial re-hydration and subjected to the staining protocol described above.

LipidTOX staining was performed using PBS containing Saponin after fixation, instead of PBST. Cuticles were stained with LipidTOX (1/500 in PBS + 0.005% Saponin) for 2h at room temperature followed by 3x15-min washes in PBS + Saponin and mounted as above, without spacers.

Most confocal images were acquired using a Zeiss 710 LSM confocal microscope. *Trans*-Tango experiments were imaged with a Zeiss LSM 800 with Airyscan confocal microscope to achieve higher resolution of neuronal populations.

Antibody concentrations used are as follows: anti-GFP (1:100), anti-pros (1:20), anti-Burs (Peabody et al., 2008) (1:200), anti-AKH (Lee and Park, 2004) (1:250), anti-pH3S10 (1:100), anti-pH3S28 (1:100), anti-Brp (1:20). Secondary antibodies were used as follows: anti-IgG-488 (1:200), anti-IgG-546 (1:100), anti-IgG-594 (1:100), anti-IgG-647 (1:100).

Quantification of pH3^{+ve} Cells in the Posterior Midgut

Antibodies against phosphorylated Histone 3 were used to assess Intestinal Stem Cell (ISC) proliferation in the posterior midgut. A minimum of 9 midguts were quantified per condition and genotype.

Protein Extraction

Proteins were extracted by homogenization of whole flies in cold RIPA buffer for 30 min with regular agitation. After centrifugation at 4°C for 10 min at a speed of 13,000 g, supernatants were collected and stored at -80°C.

Haemolymph Extraction

Haemolymph was extracted by decapitating flies and transferring them upside down into a 10 μl filter pipette tip inserted into a 20 μl pipette tip end cut in a 45° angle and placed in a 2 ml Eppendorf tube on ice. Decapitated flies were centrifuged at 10,000 g for 15 min at 4°C and the haemolymph collected and stored at -80°C. Protein concentration was quantified using Bradford assay.

SDS-PAGE and Protein Transfer

Reducing agent and loading buffer were added to protein extracts followed by heat treatment for 10 min at 95°C. Samples were centrifuged at 4°C for 10 min at 13,000 g and loaded onto a 10% Bis-Tris pre-cast gel. Protein were separated at 100 V for 45 min in 1x NuPAGE MES running buffer using the Invitrogen XCell *SureLock* electrophoresis system. 4 μl of PageRuler prestained marker was used to estimate protein size. Gels were transferred onto PVDF membrane (Amersham) and blocked with 5% BSA in TBST for 1 h at room temperature. Membranes were incubated with primary antibody in TBST containing 5 % BSA over night at 4°C. Antibody concentrations used were as follows: anti-Burs (Scopelliti et al., 2016) (1:500) and anti- αTub (1:1,000). Membranes were washed 3 times for 15 min in TBST and incubated with fluorescent IRDye (680RD and 800CW) secondary antibody (1:10,000) for 2 h at room temperature in 5% BSA/TBST . Membranes were washed and blots were visualised using the LiCor ODYSSEY Clx. Fluorescent intensity was measured using the Fiji software.

Oxygen Consumption

Mitochondrial respiration was monitored using an oxygen electrode and analysed with the OxygraphPlus software. Briefly, mitochondria from 4 females were isolated using a Mitochondria Isolation Kit and following the manufacturer's instructions. Mitochondral extracts were resuspended in 300 μl of buffer containing 10 mM HEPES, pH 7.5; 250 mM sucrose; 1 mM ATP; 80 μM ADP; 5 mM sodium succinate; 2 mM K_2HPO_4 and 1 mM DTT, and transferred into the Oxygraph chamber. The chamber was closed and basal respiration was measured for approximately 10 min. Values obtained were normalized by total protein. Data shown represent at least 3 independent experiments.

Metabolomic Analysis by Liquid Chromatography- Mass Spectrometry (LC-MS)

Flies of the desired genotype were fed for 6h with 20mg/ml $^{13}C_6$-D-Glucose or $^{12}C_6$-D-Glucose (background control) diluted in H_2O containing Allura red (1mg/ml). The 6h feeding time point was selected as the most optimal one following a prior time-course experiment and assessment of $^{13}C_6$-D-Glucose incorporation (data not shown). Allura red was used to visually monitor food content inside the animals. Animals with apparent uniform amount of Allura red in their abdomen were selected for our metabolomic studies to prevent biases in the results introduced by differences in feeding. Accurate values of Allura red content in each animal were later obtained through our mass spectrometry analysis and were used to normalize the values of $^{13}C_6$-labelled metabolites. 3 whole flies in quadruplicates for each genotype were lysed on ice with a motorised pestel in 250 μl LC-MS lysis buffer containing acetonitrile

(30% v/v), methanol (50% v/v) and MilliQ filtered water (20% v/v). Samples were cleared by centrifugation and the supernatant was transferred to LC-MS vials. LC-MS measurements and related data analysis were performed as described previously (Maddocks et al., 2017), using a ZIC-pHILIC analytical column. In short, LC-MS raw data was converted into mzML files using ProteoWizard. MZMine 2.10 was used for peak extraction and sample alignment. Data is shown as arbitrary units (AU) defined as metabolite peak area / Allura red peak area.

RNA Extraction and qRT-PCR

Total RNA was extracted using either Trizol or Qiagen RNAeasy kit, following the manufacturer's instructions. RNA extraction were performed using a minimum of 3 whole animals, 40 heads or 80 cuticles. RNA was quantified using a NanoDrop 2000c Spectrophotometer.

cDNA was synthesised using the High-Capacity cDNA reverse transcription kit in technical triplicates. Quanta SYBR green Master Mix (Low ROX, Fermentas) was used following manufacturer's instructions. Data were extracted and analysed using the Applied Biosystems 7500 software. Results represent a minimum of three biological replicates ±SEM. Expression of target genes was measured and normalised to *rpl32*, *sdha* or *actin5c.* Primers used for RT-qPCRs are shown as part of the Supplementary data.

Lipid Quantification

5 female flies were collected for each biological replicate. Biological triplicates were used for each genotype. Whole flies were lysed in 1% Triton X-100/Chlorophorm on ice for 30 minutes. Extracts were cleared by centrifugation for 15 minutes at 14,000g.

A Free Fatty Acid Kit was used to assess Free fatty acids (FFA) directly from lipid extracts following the manufacturer's instructions. Total lipid content was assessed following lipase treatment of extracts, FFA values were subtracted from total lipids to obtain triacylglycerides (TAG) content values.

Glucose Quantification

Haemolymph was extracted as described above and glucose levels within were quantified in biological triplicates using the Glucose Colorimetric Assay Kit according to manufacturer's instructions.

Locomotor Assay

A single female fly was transferred into a food-containing 6 cm tissue culture dish and movement of 2 flies in 2 separete dishes was recorded for 500 sec. Distance between the 6 cm dish and camera was kept constant. Videos were converted into image sequence (2 frames per second) using QuickTime Pro and locomotor activity was measured from at least 4 individual flies using the manual tracking plugin in Fiji.

Chill Coma Recovery Assay

Flies were aged for 3 days at 29°C. Up to 10 females were placed in a fresh vial and subjected to an ice-water bath for 10 min to induce chill coma. Recovery time at room temperature, measured by the fly's ability to stand, was recorded. Log-rank (Mantel-Cox) test was used to analyse statistical significance. Number of animals used per experiment are stated in the figures.

Glucose Absorption Assay

2-NBDG (2mg/ml) was diluted in a 5% sucrose solution containing Allura red (1mg/ml) to monitor feeding and applied to Whatman paper circles to feed flies overnight. The next day, flies were transferred onto vials containing normal fly food and aged for another 1.5-2 days. Flies still displaying red food traces were discarded. Biological triplicates or quadruplicates of 5 female flies each fed on 2-NBDG diet and one replicate of 5 control diet fed females were collected. Flies were lysed in PBST. Lysates were centrifuged and supernatant collected into a new Eppendorf tube. Fluorescent intensity was measured using the TECAN Safire^2 plate reader. Fluorescent intensity of control diet fed animals (auto-fluorescence) was subtracted from measurements obtained from 2-NBDG fed animals.

Food Intake Assay

Flies were fed with Allura red containing fly food for 2h. 25 flies were lysed in PBST, centrifuged and absorbance of Allura red in the supernatant was measured using the TECAN Sunrise plate reader. Experiments were done at least in biological triplicates.

Excretion Assay

To measure lipids and glucose within the excrement from flies, 1.5 ml Eppendorf tubes were de-capped and the lids filled with standard fly food containing a 5% Brilliant Blue FCF to allow normalisation by amount of excrement. 5 female flies were pre-fed with food containing Brilliant Blue FCF, before put into an Eppendorf tube, which was closed with a blue food-containing lid and kept at 29°C overnight. The next day, flies and food-containing lid were discarded. 100 μl of PBST was added and tube closed with a fresh empty lid, vortexed and absorbance of Brilliant Blue FCF was measured as a read-out for amount of excrement in at least biological triplicates using the TECAN Sunrise plate reader. Furthermore, lipids and glucose were measured as described above.

QUANTIFICATION AND STATISTICAL ANALYSIS

Statistical parameters and n values are indicated in figure legends. Briefly, we used Graph Pad Prism 7 software for data quantification and the generation of graphs. We used t-test to compare data within two groups or One-way ANOVA followed by Turkey's multiple comparisons test for comparisons between 3 or more groups. Error bars represent mean ± SEM. Survival curves were analysed using curve comparison and Log-Rank (Mantel-Cox) test.

Cell Metabolism, Volume *29*

Supplemental Information

A Neuronal Relay Mediates a Nutrient Responsive Gut/Fat Body Axis Regulating Energy Homeostasis in Adult *Drosophila*

Alessandro Scopelliti, Christin Bauer, Yachuan Yu, Tong Zhang, Björn Kruspig, Daniel J. Murphy, Marcos Vidal, Oliver D.K. Maddocks, and Julia B. Cordero

Supplementary Figure Legends

Figure S1. Related to Figure 1: Bursα is not detectable in adult CNS. (A) Bursα immunofluorescence (green/grey) in the CNS of dark pupae and 10-14d old adult fed and 24h starved wild type flies. Nc82 staining labels the neuropil (red). Scalebars = 200 μm. (B) Relative *bursα* mRNA levels in midguts only and gut-less wild type flies (10-14d old) fully fed or following 24h starvation. (C) Relative *Glut1* mRNA levels in midguts of flies following 14d of transgene activation. Data represents the average of at least 3 biological replicates. Statistical analysis was done by unpaired t-test. Bars represent mean ± SEM. (D) Immunostaining of adult posterior midguts from fed and starved flies bearing ee-specific overexpression of *bursα* for 10-14d. Bursα (red/white). Unless otherwise noted DAPI (blue) was used to stain all cell nuclei. (E) Relative TAG levels in 14d old animals of indicated genotypes. 5 females were collected for each biological replicate. Data for each genotype represents the mean of 3 biological replicates ± SEM. Statistical analysis was done by one-way ANOVA followed by Turkey's multiple comparisons test.

Figure S2. Related to Figure 2: Disruption of Bursα/DLgr2 signalling does not affect food intake, absorption and excretion or locomotor activity. (A, B) Food intake assay between Zeitgeber (ZT) 22-24 in 3d old adults (A) and adults following 14d transgene activation (B) by measuring Allura Red absorbance in whole fly extracts. 25 animals were analysed per genotype per biological replicate. Data represents the average of 3 biological replicates ± SEM. Statistical analysis was done through unpaired t-test. (C, D) Fluorescent intensity of 2-NBDG accumulation in whole fly extracts from animals of ages and genotypes as in A, B. 5 animals were analysed

per genotype per biological replicate. Data represents the average of a minimum of 3 biological replicates ± SEM. Statistical analysis was done through unpaired t-test. (E-G) Glucose (E), TAG (F) and FFA levels (G) levels in excrements of adult animals following 14d of transgene activation. 5 animals were analysed per genotype per biological replicate. Data represents the average of at least 3 biological replicates ± SEM. Statistical analysis was done through unpaired t-test. (H) Relative circulating glucose levels in 3d old animals. Significances are shown compared to their own control. Data for each genotype represents the mean of 3 biological replicates ± SEM. Statistical analysis was done through unpaired t-test. (I, J) Relative circulating TAG (I) and FFA (J) in flies following 14d of transgene activation. 5 females were collected for each biological replicate. Data for each genotype represents the mean of 3 biological replicates ± SEM. Statistical analysis was done by unpaired t-test. (K, L) Results from video-tracking analysis to assess locomotor activity of animals following 14d transgene activation (K) and 3d old whole mutant animals (L). We assessed the locomotion of a 4 or 5 individual flies per genotype. Each experimental genotype was compared to its own control. Data for each genotype represents mean values ± SEM. Statistical analysis was done through unpaired t-test (K) and one-way ANOVA followed by Turkey's multiple comparisons test.

Figure S3. Related to Figure 3: Bursα signalling via VM, fat body or tracheal DLgr2 is dispensable for metabolic regulation. (A) Immunofluorescent staining of adult posterior midguts (R4-R5 transition) expressing *CD8-GFP* (green) under the control of a *dlgr2*-Gal4 driver. (B, I, K, M) Starvation survival analysis in animals started 10d after transgene activation. Total number of animals assessed (n) is indicated within the graphs. Log-rank (Mantel-Cox) test was used to analyse statistical

significance. (C) Relative circulating glucose levels in animals following 14d of transgene activation. Data for each genotype represents the mean of 3 biological replicates ± SEM. Statistical analysis was done through unpaired t-test. (D, E, H, L, N, O) Relative TAG levels from adult animals following 14d of transgene activation (D, H, L, N) or from 7d old whole mutant animals with or without concomitant expression of the indicated transgenes (E, O). 5 females were collected for each biological replicate. Data for each genotype represents the mean of 3 biological replicates ± SEM. Statistical analysis was done by unpaired t-test (D, H, L, N) and one-way ANOVA followed by Turkey's multiple comparisons test (E, O). (F, G) Quantifications from Western Blots of haemolymph Bursα. Data represents the mean from 3 independent experiments of the type shown in Fig. 3A, B ± SEM. Statistical analysis was done through unpaired t-test. (J) Immunofluorescent staining in fat bodies from adults expressing nuclear *Red stinger* (red) under the control of a *dlgr2*-Gal4 driver.

Figure S4. Related to Figures 3 and 4: Loss of lipid storage upon neuronal knockdown of *dlgr2* is independent from food intake, absorption and excretion. (A) Food intake assessment within ZT 22-24 in flies of the indicated genotypes analysed by measuring Allura Red absorbance in whole fly extracts. 25 animals were analysed per genotype per biological replicate. Data represents the average of 3 biological replicates ± SEM. Statistical analysis was done through unpaired t-test. (B, C) Relative glucose (B) and FFA levels (C) in the excrement in flies of indicated genotypes following 14d of transgene activation. 5 animals were analysed per genotype per biological replicate. Data represents the average of 3 biological replicates ± SEM. Statistical analysis was done through unpaired t-test. (D) Intestinal glucose absorption in flies of genotypes as in (A-C) measured by analysing 2-NBDG

fluorescent intensity in whole fly extracts. 5 animals were analysed per genotype per biological replicate. Data represents the average of at least 3 biological replicates ± SEM. Statistical analysis was done through unpaired t-test. (E) Chill Coma Recovery Time was recorded in animals following 3d adult specific transgene activation. Total number of animals assessed (n) is indicated within the graphs. Log-rank (Mantel-Cox) test was used to analyse statistical significance. (F) Relative oxygen consumption of whole fly mitochondrial extracts from animals following 3d adult specific transgene activation. 4 whole animals where used per biological replicate. Data for each genotype represents the mean of 4 biological replicates ± SEM. Statistical analysis was done through paired t-test.

Figure S5. Related to Figures 3 and 4: Insulin signalling is not affected by Bursα/ neuronal DLgr2. (A, B) qRT-PCRs for *inr* and *thor* relative to *sdha* in cuticles (A) and for *dilp2*, *3* and *5,* relative to *actin5c* in heads (B). Significances are shown relative to their individual control. Data represents the average of at least 3 biological replicates. Statistical analysis was done by unpaired t-test. Bars represent mean ± SEM. (C) Immunofluorescence staining in fat bodies of adults expressing the Insulin sensor tGPH (green). All experiments were carried out 14d following activation of the indicated transgenes.

Figure S6. Related to Figure 5: Indirect regulation of AKH/AKHR signalling by DLgr2. (A) Immunofluorescence staining of adult Corpora Cardiaca neurons expressing *Myr-GFP* under the control of a *dlgr2*-Gal4 driver (green) and co-stained with anti-AKH antibody (red). DAPI (blue). Scalebars= 100 μm (left) and 20 μm (right). (B) Starvation survival test in animals of indicated genotypes following transgene

activation for 10d. Total number of animals assessed (n) is indicated within the graphs. Log-rank (Mantel-Cox) test was used to analyse statistical significance. (C) Relative TAG levels in whole flies of the indicated genotypes following 14d transgene activation (C). 5 females were collected for each biological replicate. Data for each genotype represents the mean of 3 biological replicates ± SEM. Statistical analysis was done by unpaired t-test. (D) Immunofluorescent staining in Corpora Cardiaca neurons expressing *CD8-RFP* under the *nSyb*-Gal4 driver (red) and co-stained with anti-AKH antibody (green). DAPI (blue). (E) *akh* transcript levels relative to *rpl32* in whole animals following 14d transgene activation. Data represents the average of 3 biological replicates. Statistical analysis was done by unpaired t-test. Bars represent mean ± SEM. (F-I) Relative TAG levels in 14d old animals of the indicated genotypes (UAS-RNAi-only controls for Fig. 5F) (F) or in 7d old animals of indicated genotypes (UAS-RNAi-only controls for Fig. 5M) (G-I). 5 females were collected for each biological replicate. Data for each genotype represents the mean of 3 biological replicates ± SEM. Statistical analysis was done by one-way ANOVA followed by Turkey's multiple comparisons test. (J) pH3 staining to visualize ISC proliferation in posterior midguts of animals of indicated genotypes following 14d of transgene activation. Animals were either fully fed (black) or fed followed by 24h starvation prior to pH3 assessment (red). A minimum of 9 midguts were analysed per genotype per condition. Each dot in the graph (n) represents an individual posterior midgut. n values from left to right = 16 (+, Fed and Starved); 13 (*Glut1 IR*, Fed); 14 (*Glut1 IR*, Starved); 9 (*bursα IR*, Fed); 21 (*bursα IR*, Starved); 10 (+, Fed); 20 (+, Starved); 10 (*osbp*, Fed); 11 (*osbp*, Starved). Statistical analysis was done by unpaired t-test. Bars represent mean ± SEM.

Table S1: Primer sequences used in this study. Related to STAR Methods.

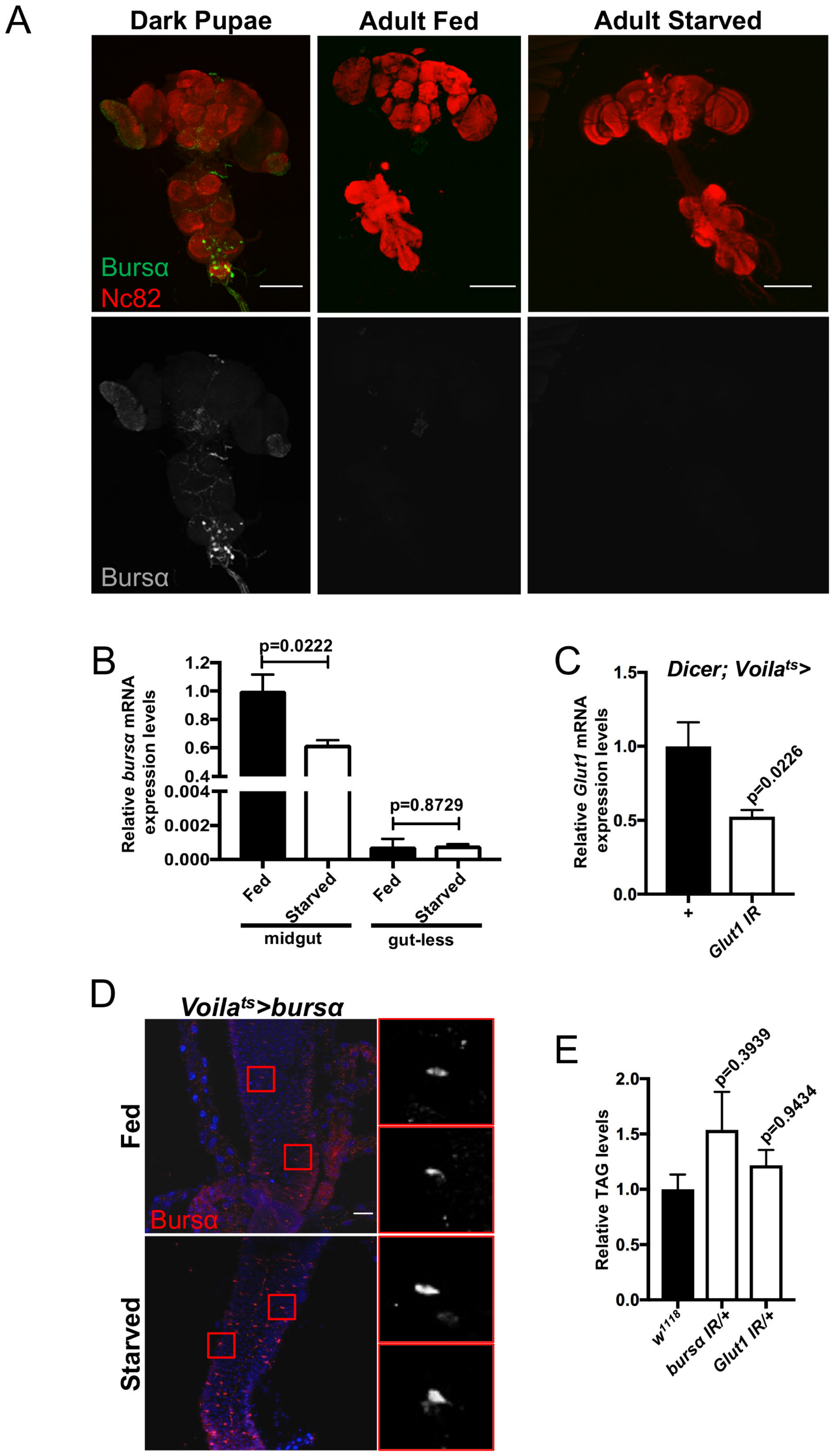
A
Dark Pupae
Adult Fed
Adult Starved
Bursα
Nc82
Bursα
B
p=0.0222
p=0.8729
Relative bursα mRNA expression levels
1.2
1.0
0.8
0.6
0.4
0.004
0.002
0.000
Fed
Starved
Fed
Starved
midgut
gut-less
C
Dicer; Voila^ts>
Relative Glut1 mRNA expression levels
1.5
1.0
0.5
0.0
p=0.0226
+
Glut1 IR
D
Voila^ts>bursα
Fed
Starved
Bursα
E
Relative TAG levels
2.0
1.5
1.0
0.5
0.0
p=0.3939
p=0.9434
w^1118
bursα IR/+
Glut1 IR/+

Figure S1

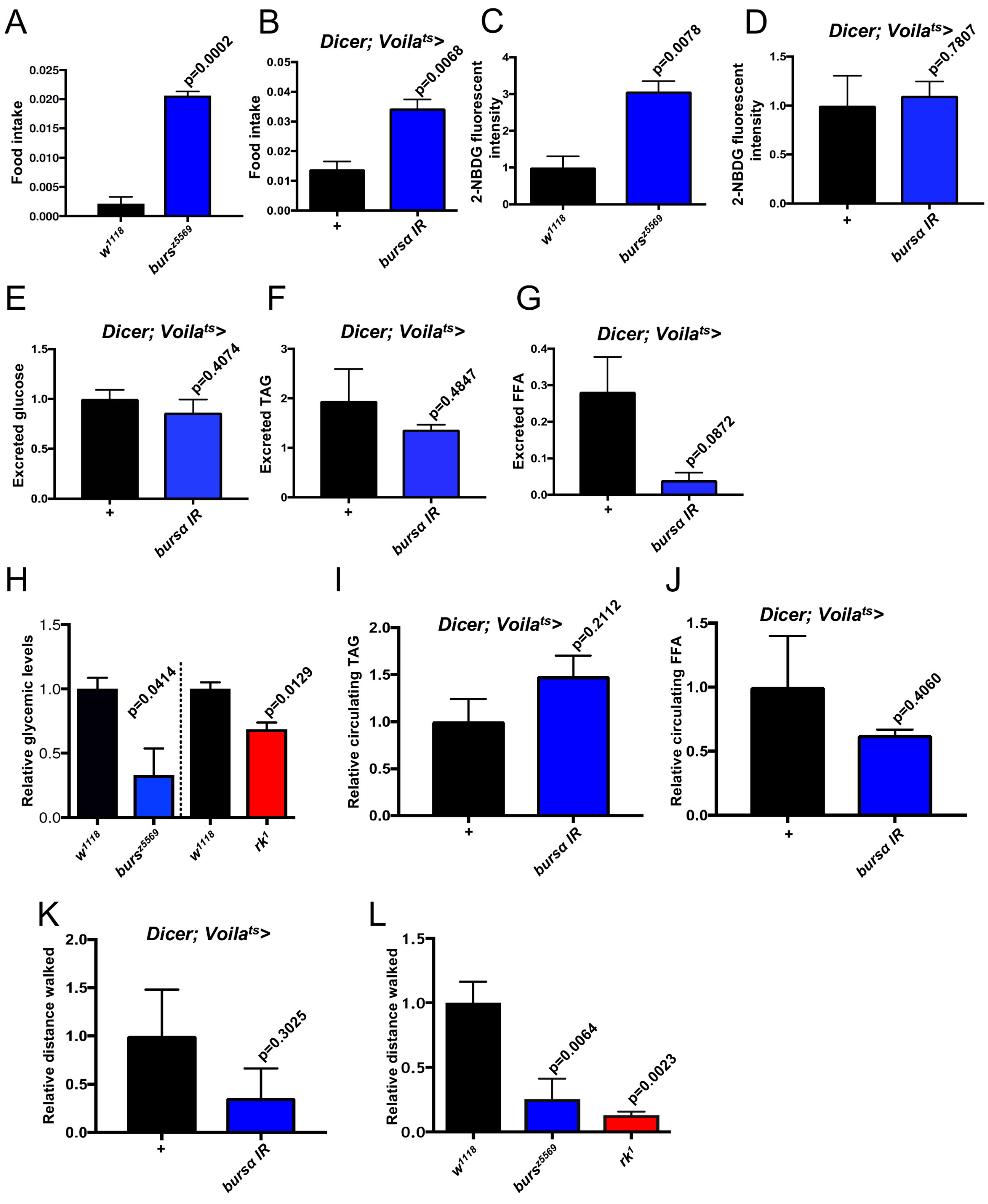

A
Food intake
p=0.0002
w1118
bursz5569
B
Dicer; Voilats>
Food intake
p=0.0068
+
bursα IR
C
2-NBDG fluorescent intensity
p=0.0078
w1118
bursz5569
D
Dicer; Voilats>
2-NBDG fluorescent intensity
p=0.7807
+
bursα IR
E
Dicer; Voilats>
Excreted glucose
p=0.4074
+
bursα IR
F
Dicer; Voilats>
Excreted TAG
p=0.4847
+
bursα IR
G
Dicer; Voilats>
Excreted FFA
p=0.0872
+
bursα IR
H
Relative glycemic levels
p=0.0414
p=0.0129
w1118
bursz5569
w1118
rk1
I
Dicer; Voilats>
Relative circulating TAG
p=0.2112
+
bursα IR
J
Dicer; Voilats>
Relative circulating FFA
p=0.4060
+
bursα IR
K
Dicer; Voilats>
Relative distance walked
p=0.3025
+
bursα IR
L
Relative distance walked
p=0.0064
p=0.0023
w1118
bursz5569
rk1

Figure S2

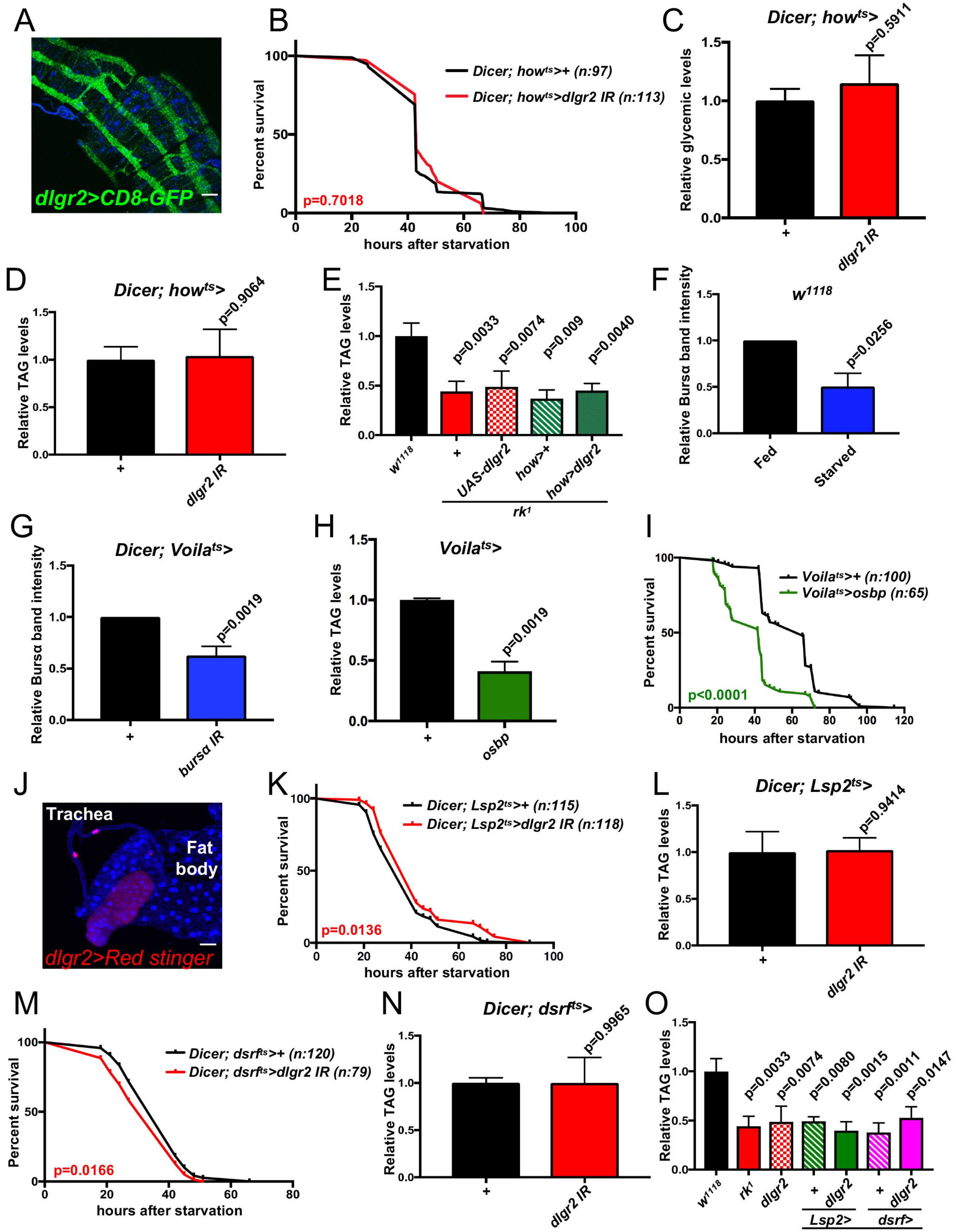
A
dlgr2>CD8-GFP
B
Percent survival
hours after starvation
Dicer; howts>+ (n:97)
Dicer; howts>dlgr2 IR (n:113)
p=0.7018
C
Dicer; howts>
Relative glycemic levels
p=0.5911
+
dlgr2 IR
D
Dicer; howts>
Relative TAG levels
p=0.9064
+
dlgr2 IR
E
Relative TAG levels
p=0.0033
p=0.0074
p=0.009
p=0.0040
w1118
+
UAS-dlgr2
how>+
how>dlgr2
rk1
F
w1118
Relative Bursα band intensity
p=0.0256
Fed
Starved
G
Dicer; Voilats>
Relative Bursα band intensity
p=0.0019
+
bursα IR
H
Voilats>
Relative TAG levels
p=0.0019
+
osbp
I
Percent survival
hours after starvation
Voilats>+ (n:100)
Voilats>osbp (n:65)
p<0.0001
J
Trachea
Fat body
dlgr2>Red stinger
K
Percent survival
hours after starvation
Dicer; Lsp2ts>+ (n:115)
Dicer; Lsp2ts>dlgr2 IR (n:118)
p=0.0136
L
Dicer; Lsp2ts>
Relative TAG levels
p=0.9414
+
dlgr2 IR
M
Percent survival
hours after starvation
Dicer; dsrfts>+ (n:120)
Dicer; dsrfts>dlgr2 IR (n:79)
p=0.0166
N
Dicer; dsrfts>
Relative TAG levels
p=0.9965
+
dlgr2 IR
O
Relative TAG levels
p=0.0033
p=0.0074
p=0.0080
p=0.0015
p=0.0011
p=0.0147
w1118
rk1
dlgr2
+
dlgr2
+
dlgr2
Lsp2>
dsrf>
rk1

Figure S3

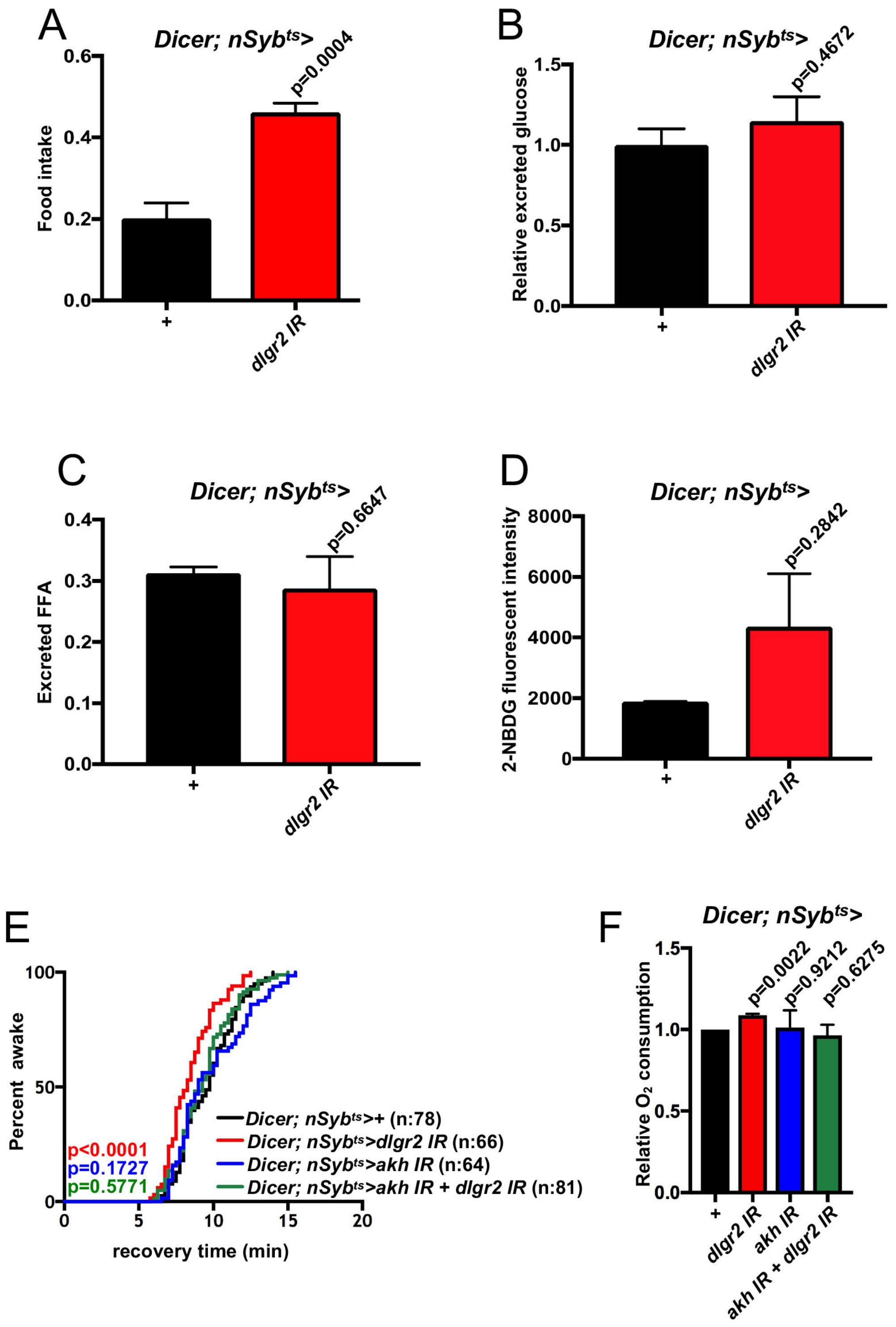
A
Dicer; nSyb^ts>
p=0.0004
Food intake
0.6
0.4
0.2
0.0
+
dlgr2 IR
B
Dicer; nSyb^ts>
p=0.4672
Relative excreted glucose
1.5
1.0
0.5
0.0
+
dlgr2 IR
C
Dicer; nSyb^ts>
p=0.6647
Excreted FFA
0.4
0.3
0.2
0.1
0.0
+
dlgr2 IR
D
Dicer; nSyb^ts>
p=0.2842
2-NBDG fluorescent intensity
8000
6000
4000
2000
0
+
dlgr2 IR
E
Percent awake
100
50
0
p<0.0001
p=0.1727
p=0.5771
Dicer; nSyb^ts>+ (n:78)
Dicer; nSyb^ts>dlgr2 IR (n:66)
Dicer; nSyb^ts>akh IR (n:64)
Dicer; nSyb^ts>akh IR + dlgr2 IR (n:81)
0
5
10
15
20
recovery time (min)
F
Dicer; nSyb^ts>
Relative O2 consumption
1.5
1.0
0.5
0.0
p=0.0022
p=0.9212
p=0.6275
+
dlgr2 IR
akh IR
akh IR + dlgr2 IR

Figure S4

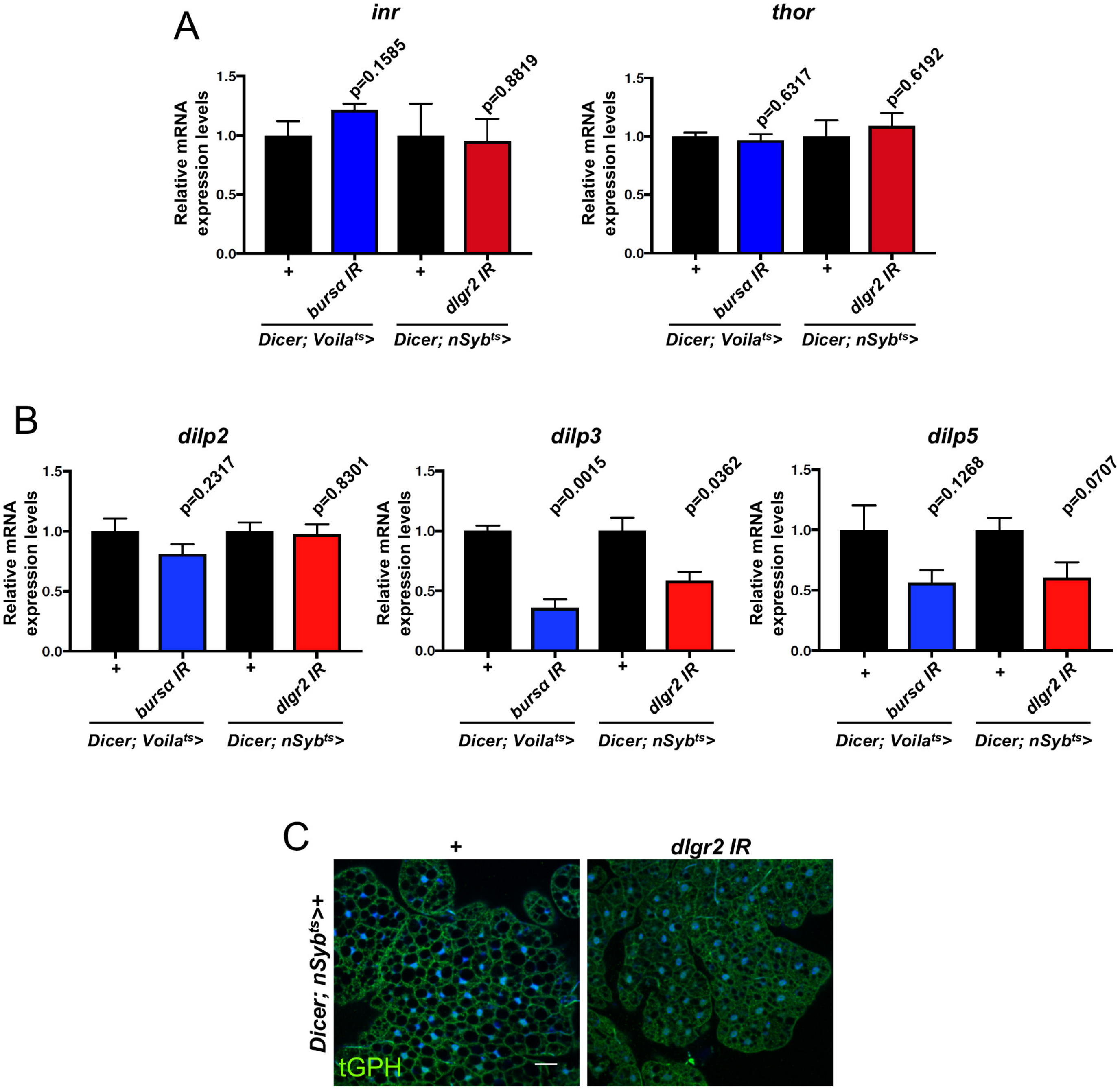
A
inr
thor
Relative mRNA expression levels
p=0.1585
p=0.8819
p=0.6317
p=0.6192
+
bursα IR
dlgr2 IR
Dicer; Voila^ts>
Dicer; nSyb^ts>
B
dilp2
dilp3
dilp5
p=0.2317
p=0.8301
p=0.0015
p=0.0362
p=0.1268
p=0.0707
C
dlgr2 IR
Dicer; nSyb^ts>+
tGPH

Figure S5

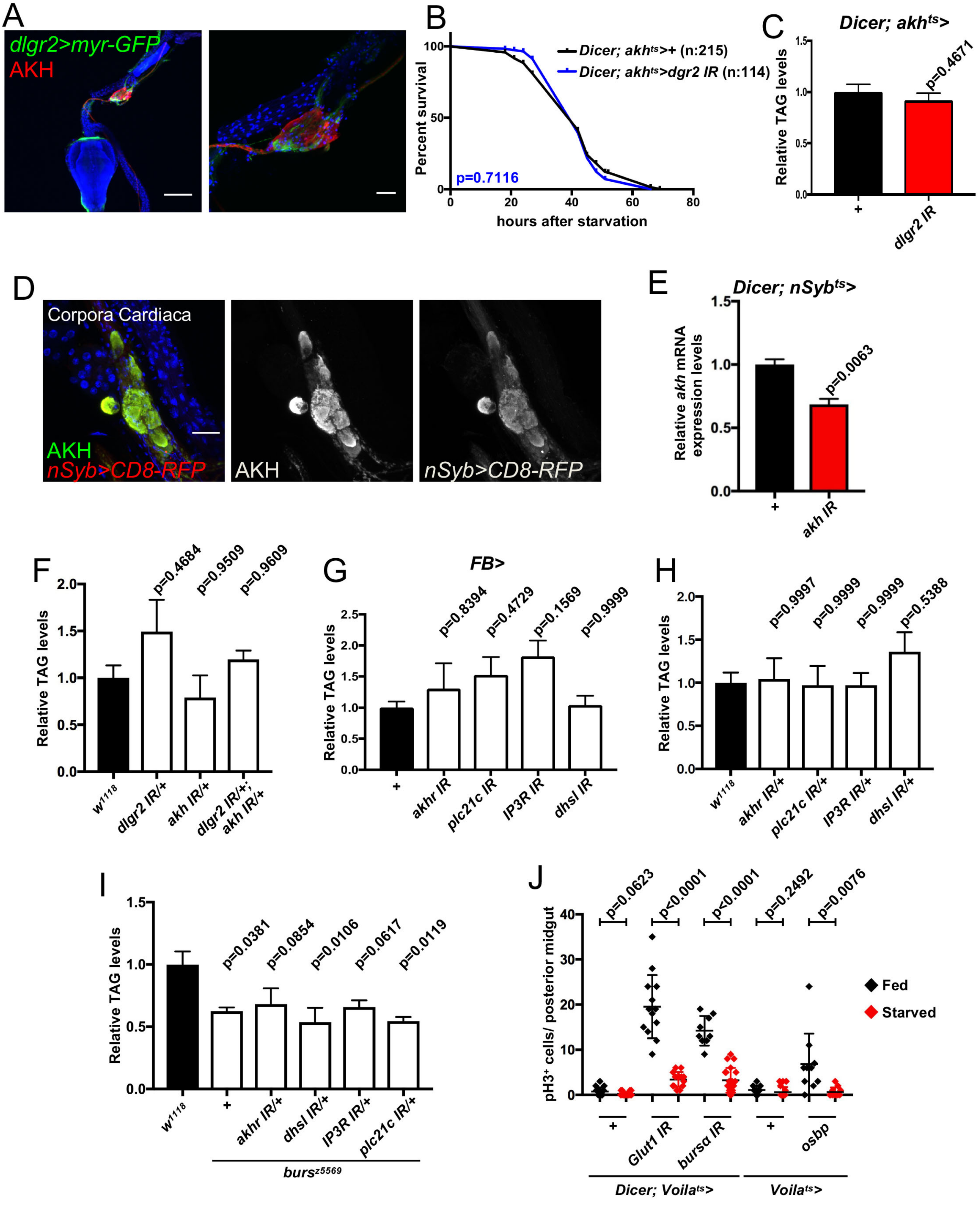
A
dlgr2>myr-GFP
AKH
B
Dicer; akhts>+ (n:215)
Dicer; akhts>dgr2 IR (n:114)
Percent survival
p=0.7116
hours after starvation
C
Dicer; akhts>
Relative TAG levels
p=0.4671
+
dlgr2 IR
D
Corpora Cardiaca
AKH
nSyb>CD8-RFP
AKH
nSyb>CD8-RFP
E
Dicer; nSybts>
Relative akh mRNA expression levels
p=0.0063
+
akh IR
F
Relative TAG levels
p=0.4684
p=0.9509
p=0.9609
w1118
dlgr2 IR/+
akh IR/+
dlgr2 IR/+; akh IR/+
G
FB>
Relative TAG levels
p=0.8394
p=0.4729
p=0.1569
p=0.9999
+
akhr IR
plc21c IR
IP3R IR
dhsl IR
H
Relative TAG levels
p=0.9997
p=0.9999
p=0.9999
p=0.5388
w1118
akhr IR/+
plc21c IR/+
IP3R IR/+
dhsl IR/+
I
Relative TAG levels
p=0.0381
p=0.0854
p=0.0106
p=0.0617
p=0.0119
w1118
+
akhr IR/+
dhsl IR/+
IP3R IR/+
plc21c IR/+
bursz5569
J
pH3+ cells/ posterior midgut
p=0.0623
p<0.0001
p<0.0001
p=0.2492
p=0.0076
Fed
Starved
+
Glut1 IR
bursα IR
+
osbp
Dicer; Voilats>
Voilats>

Figure S6

Target	Primer name	Sequence 5' → 3'
rpl32	rpl32 F1	AGGCCCAAGATCGTGAAGAA
	rpl32 R1	TGTTGCACCAGGAACTTCTTGAA
actin5c	actin5c F1	GAGCGCGGTTACTCTTTCAC
	actin5c R1	CCATCTCCTGCTCAAAGTCG
sdha	sdha F1	AATGCCCAGATGACTATTGTGAG
	sdha R1	GCTTGCTGAAATCGTATTCATCC
bursα	burs F1	CATCCATGTGCTCCAGTATCC
	burs R1	GGCTTCACTTTGGGACAGAA
dilp2	dilp2 F1	CCTGCAGTTTGTCCAGGAGT
	dilp2 R1	AGCCAGGGAATTGAGTACACC
dilp3	dilp3 F1	GTATGGCTTCAACGCAATGAC
	dilp3 R1	GAGCATCTGAACCCAACTATCAC
dilp5	dilp5 F1	CGTGATCCCAGTTCTCCTGT
	dilp5 R1	ACCCTCAGCATGTCCATCAA
thor	thor F1	CCAGGAAGGTTGTCATCTCG
	thor R1	TGAAAGCCCGCTCGTAGATA
inr	inr F1	GGTGCTGGCATCATAGGTCT
	inr R1	CCTGCCTCTGAGTGATAGAAGG
akh	akh F1	GAAGTGGACCAGCATAGAACTC
	akh R1	GCGAGAAGGTCAATTGACAC

Table S1: Primer sequences used in this study. Related to STAR Methods.
